# MN15: A Kohn–Sham global-hybrid exchange–correlation density functional with broad accuracy for multi-reference and single-reference systems and noncovalent interactions†
†Electronic supplementary information (ESI) available: Mean unsigned errors of Database 2015B for 84 functionals and geometries of databases ABDE13, S6x6, SBG31, and EE69. See DOI: 10.1039/c6sc00705h


**DOI:** 10.1039/c6sc00705h

**Published:** 2016-04-06

**Authors:** Haoyu S. Yu, Xiao He, Shaohong L. Li, Donald G. Truhlar

**Affiliations:** a Department of Chemistry , Chemical Theory Center , Inorganometallic Catalyst Design Center , Minnesota Supercomputing Institute , University of Minnesota , Minneapolis , Minnesota 55455-0431 , USA . Email: truhlar@umn.edu; b State Key Laboratory of Precision Spectroscopy and Department of Physics , East China Normal University , Shanghai , 200062 , China; c NYU-ECNU Center for Computational Chemistry at NYU Shanghai , Shanghai , 200062 , China

## Abstract

Kohn–Sham density functionals are widely used; however, no currently available exchange–correlation functional can predict all chemical properties with chemical accuracy. Here we report a new functional, called MN15, that has broader accuracy than any previously available one. The properties considered in the parameterization include bond energies, atomization energies, ionization potentials, electron affinities, proton affinities, reaction barrier heights, noncovalent interactions, hydrocarbon thermochemistry, isomerization energies, electronic excitation energies, absolute atomic energies, and molecular structures. When compared with 82 other density functionals that have been defined in the literature, MN15 gives the second smallest mean unsigned error (MUE) for 54 data on inherently multiconfigurational systems, the smallest MUE for 313 single-reference chemical data, and the smallest MUE on 87 noncovalent data, with MUEs for these three categories of 4.75, 1.85, and 0.25 kcal mol^–1^, respectively, as compared to the average MUEs of the other 82 functionals of 14.0, 4.63, and 1.98 kcal mol^–1^. The MUE for 17 absolute atomic energies is 7.4 kcal mol^–1^ as compared to an average MUE of the other 82 functionals of 34.6 kcal mol^–1^. We further tested MN15 for 10 transition-metal coordination energies, the entire S66x8 database of noncovalent interactions, 21 transition-metal reaction barrier heights, 69 electronic excitation energies of organic molecules, 31 semiconductor band gaps, seven transition-metal dimer bond lengths, and 193 bond lengths of 47 organic molecules. The MN15 functional not only performs very well for our training set, which has 481 pieces of data, but also performs very well for our test set, which has 823 data that are not in our training set. The test set includes both ground-state properties and molecular excitation energies. For the latter MN15 achieves simultaneous accuracy for both valence and Rydberg electronic excitations when used with linear-response time-dependent density functional theory, with an MUE of less than 0.3 eV for both types of excitations.

## Abstract 2

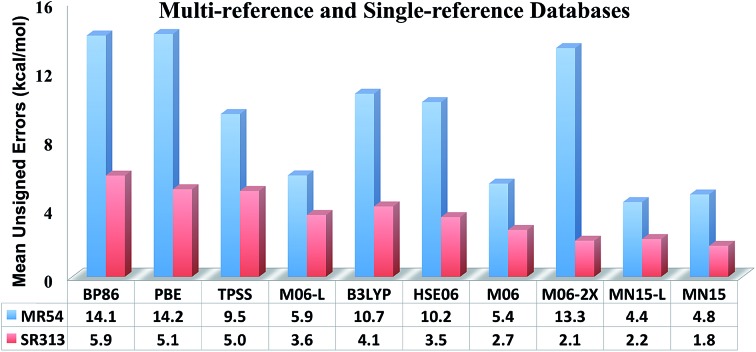
We report a global-hybrid approximation, MN15, to the exchange–correlation functional of Kohn–Sham theory with broadly accurate performance for both multi-reference and single-reference systems.

## 1. Introduction

In 1927–30, Thomas, Fermi, and Dirac proposed treating the energies of systems of many electrons by a statistical model of the electron density *ρ*.^1^–^3^ However, Teller proved that no such model could predict the existence of stable molecules.^4^ In 1951 Slater^5^ proposed a set of self-consistent-field (SCF) equations involving effective local potentials defined in terms of *ρ* by the free-electron gas model, and these equations do predict molecular binding. In 1964, Hohenberg and Kohn proved that the ground-state energy of a system can be written as a universal functional *F* of *ρ*,^6^ and in 1965 Kohn and Sham^7^ represented the density as the one-electron density computed from the square of a Slater determinant and provided an SCF scheme for using the Hohenberg–Kohn theorem to calculate the energies of atoms, molecules, and solids. These equations resemble Slater's equations but the new derivation shows that SCF equations with local potentials are not just an approximation scheme but can be exact if Slater's approximation to exchange is replaced by a local potential derived from the so-called exact exchange–correlation functional (XCF), which is a functional of *ρ*. Unfortunately, this exact XCF is unknown and is essentially unknowable.^8^ Progress in density functional theory is dominated by attempts to obtain better approximations to this functional; although somewhat unsystematic, this progress has revolutionized the practice of modern chemistry.

We will discuss Kohn–Sham theory in the spin-unrestricted form in which the XCF is generalized to depend not just on total density but on the spin-up and spin-down densities *ρ*_α_ and *ρ*_β_ (where α and β denote spin-up and spin-down electrons).^9^,^10^ The original approximations to the XCF involved separate approximations to exchange energy density and correlation energy density, at given points in space, that are explicit functionals of the local electron spin densities *ρ*_α_ and *ρ*_β_. These are called local-spin-density approximations (LSDAs).^11^–^13^ Later work added dependence of exchange and correlation energy densities on the magnitudes *s*_α_ and *s*_β_ of the reduced gradients of *ρ*_α_ and *ρ*_β_, leading to generalized gradient approximations (GGAs)^14^–^16^ and on the local kinetic energy densities *τ*_α_ and *τ*_β_ of spin-up and spin-down electrons,^17^ leading to meta-GGAs. The partition of the electronic energy into exchange and correlation in exchange–correlation functionals is different from the partition usually used in wave function theory; and in Kohn–Sham theory one can also approximate exchange and correlation together, without separating them, leading to nonseparable gradient approximations (NGAs)^18^ and meta-NGAs.^19^ Note that the local kinetic energies are local functionals of the occupied Kohn–Sham orbitals.^20^ It can easily be shown^21^ though that the unknown exact functional is not a local functional of the orbitals or the densities; therefore the search for improved approximations must also consider nonlocal energy densities such as some percentage of the Hartree–Fock energy, leading to what are called hybrid functionals,^22^ and/or nonlocal approximations to electron correlation, leading to so-called van der Waals functionals^23^ or doubly hybrid functionals.^24^,^25^ There are various kinds of hybrid functionals, but we especially distinguish global hybrids, where the percentage *X* of Hartree–Fock exchange is a constant, and range-separated (RS) hybrids, where it is a nonconstant function of interelectronic distance.

Many research groups have proposed approximations to the XCF containing some or all of the elements reviewed in the previous paragraph, and we will give references to many of these below. Here we mention though that the new functional presented here builds on previous work in our group, especially the GAM^26^ functional, which is an NGA, the M06-L^27^ functional, which is a meta-GGA, the MN12-L^28^ and MN15-L^29^ functionals, which are meta-NGAs, the PW6B95,^30^ M06,^31^ M06-2X,^31^ and M08-HX^32^ functionals, which are global-hybrid meta-GGAs, and the MN12-SX^33^ functional, which is a range-separated-hybrid meta-NGA. Even though these and many functionals from other groups (many of which are listed in Section 5 and discussed in Section 6) have had considerable success, there are still some important and challenging areas where exchange–correlation functionals can be improved, and we particularly single out the difficulty of finding a single functional that is accurate both for inherently multi-configurational systems, such as many systems containing transition metals, and for barrier heights. (We will follow the common convention of labeling inherently multi-configurational systems as “multireference” systems; such systems include those with near-degeneracy correlation, also called static correlation. Systems that are qualitatively well described by a single configuration state function will be called “single-reference” systems.)

In the present article, building on the work summarized above, we present a global-hybrid meta-NGA, called MN15, that is able to achieve better simultaneous accuracy for these two properties (energies of multireference systems and barrier heights) than any previous functional and also has especially good performance for noncovalent interactions and excitation energies. Such a functional should be useful for a wide variety of application areas, including catalysis, the quest for new energy, nanotechnology, functional materials, synthesis, biochemistry, the drive toward a cleaner environment, and understanding chemical dynamics.

The article proper is kept brief for a general readership. Detailed results for specialists are presented in ESI.†

## 2. Databases

**Fig. 1:**
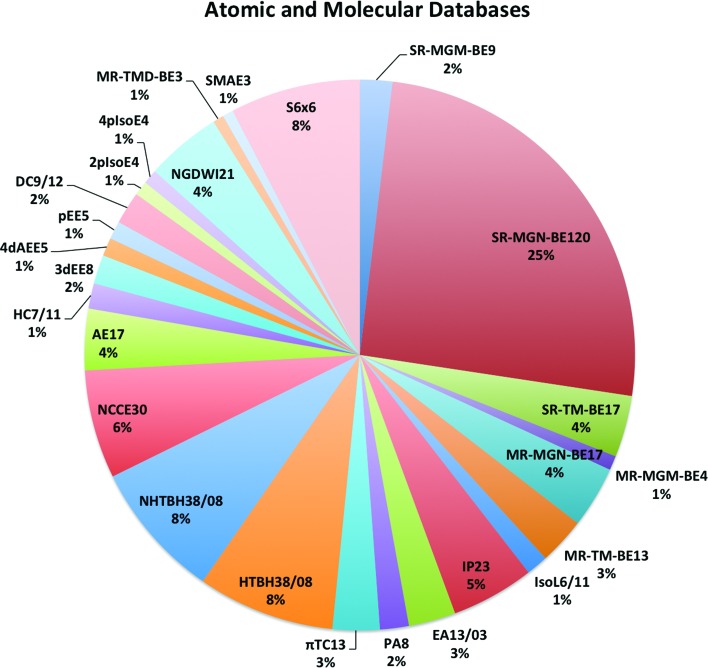
The percentage of all atomic and molecular databases (AME471), the number after a name means the number of data in this database, for example, SR-MGM-BE9 mean 9 pieces of bonding energy data in this database. The explanation of the these names are shown in Table 1.

The largest database we consider in the present paper is called Database 2015B, which has 481 data and is a combination of databases AME471 and MS10. Database AME471 contains 471 atomic and molecular energies. It is obtained by extending a previous database, AME422,^29^ by the addition of 49 new data, of which 36 are noncovalent interactions (subdatabase S6x6, which means six dimers at six intermonomer distances taken from the S66x8 database^34^) and 13 alkyl bond dissociation energies from a recent paper of our group.^35^ The distribution of data in AME471 across its various subdatabases is illustrated in Fig. 1, which shows that it is a very diverse database. Database 2015B also includes database MS10, which has ten molecular structure data, as used in a previous work.^29^ All the data in Database 2015B are shown in Table 1 with their acronyms, brief descriptions, inverse weights for training (which are explained in Section 4), and references.

**Table 1:** Databases included in Database 2015B^*a*^

*n*	Combined databases	Primary subsets	Secondary subsets	Description	*I*_*n*_ ^*b*^	Ref(s).
1–26	AME471			Atomic and molecular energies		
1–4	MGBE150			Main-group bond energies		
1		SR-MGM-BE9		Single-reference main-group metal bond energy	0.91	
			SRM2	Single-reference main-group bond energies		93
			SRMGD5	Single-reference main-group diatomic bond energies		93, 124
			3dSRBE2	3d single-reference metal–ligand bond energies		125
2		SR-MGN-BE120		Single-reference main-group non-metal bond energies		
			SR-MGN-BE107	Single-reference main-group non-metal bond energies	0.10	93
			ABDE13	Alkyl bond dissociation energies	2.00	35
3		MR-MGM-BE4		Multi-reference main-group metal bond energies	0.66	124
4		MR-MGN-BE17		Multi-reference main-group non-metal bond energies	1.00	93
5–7	TMBE33			Transition-metal bond energies		
5		SR-TM-BE17		Single-reference TM^*c*^ bond energies	1.18	
			3dSRBE4	3d single-reference metal–ligand bond energies		125
			SRMBE10	Single-reference metal bond energies		93
			PdBE2	Palladium complex bond energies		126
			FeCl	FeCl bond energy		127
6		MR-TM-BE13		Multi-reference TM bond energies	0.72	
			3dMRBE6	3d multi-reference metal–ligand bond energies		125
			MRBE3	Multi-reference bond energies		93
			Remaining	Bond energies of remaining molecules: CuH, VO, CuCl, NiCl		127
7		MR-TMD-BE3		Multi-reference TM dimer bond energies (Cr_2_ and V_2_)	1.61	93
				Multi-reference TM dimer bond energy (Fe_2_)	1.25	128
8–9	BH76			Reaction barrier heights		
8		HTBH38/08		Hydrogen transfer barrier heights	0.14	93
9		NHTBH38/08		Non-hydrogen transfer barrier heights	0.13	93
10–12	NC87			Noncovalent interactions		
10		NCCE23		Noncovalent complexation energies (23 data without charge transfer)	0.10	93, 129–132
		CT7		Seven charge transfer data	0.03	93
11		S6x6		Six dimers at six intermonomeric distances	0.013	34
12		NGDWI21		Noble gas dimer weak interaction	0.003	93, 133
13–15	EE18			Excitation energies		
13		3dEE8		3d TM atomic excitation energies and first excitation energy of Fe_2_	0.94	128, 134, 135
14		4dAEE5		4d TM atomic excitation energies	2.99	136
15		pEE5		p-block excitation energies	0.74	137
16–18	IsoE14			Isomerization energies		
16		4pIsoE4		4p isomerization energies	0.64	138
17		2pIsoE4		2p isomerization energies	3.12	138
18		IsoL6/11		Isomerization energies of large molecules	2.00	93
19–20	HCTC20			Hydrocarbon thermochemistry		
19		πTC13		Thermochemistry of π systems	3.90	93
20		HC7/11		Hydrocarbon chemistry	1.44	93
21		EA13/03		Electron affinities	0.54	93
22		PA8		Proton affinities	0.45	93
23		IP23		Ionization potentials	2.73	93, 134
24		AE17		Atomic energies	2.38	93
25		SMAE3		Sulfur molecules atomization energies	2.00	139–141
26		DC9/12		Difficult cases	10.00	93
27–29	MS10			Molecular structures		
27		DGL6		Diatomic geometries of light-atom molecules	0.009	93
28–29		DGH4				
28			DGH3	Diatomic geometries of heavy-atom molecules: ZnS, HBr, NaBr	0.008	142
29			DGH1	Diatomic geometry of Ag_2_	0.178	94

^*a*^All the databases in this table are used for both training and testing. In databases named X*n*, there are *n* data; in those named X*n*/*yy*, there are *n* data, and *yy* denotes the year of an update.

^*b*^Inverse weights with units of kcal mol^–1^ per bond for databases 1–7, kcal mol^–1^ for databases 8–26, and Å for databases 27–29.

^*c*^TM denotes transition metal.

Database 2015B was used for both training and testing of the MN15 functional, and we calculated this full database for 82 other functionals for comparison. In Table 2, we list additional databases that we used only for testing the performance of the new MN15 functional and for more limited comparisons to other functionals. These data are not used for parameterization.

**Table 2:** Databases for testing only^*a*^

*n*	Database	Description	Reference(s)
1	SBG31^*b*^	Semiconductor band gaps (31 data)	93
2	WCCR10	Ligand dissociation energies of large cationic TM complexes (10 data)	92
3	S492^*c*^	Interaction energies relevant to bimolecular structures (492 data)	34
4	TMBH21	TM reaction barrier heights (21 data)	89–91
5	EE69	Excitation energies of 30 valence and 39 Rydberg states of 11 organic molecules (69 data)	40
6	TMDBL7	Bond lengths of homonuclear TM dimers (7 data)	94
7	SE47	Semi-experimental structures of 47 organic molecules (193 data)^*c*^	95

^*a*^There are 823 data in this table; none of them were used for training.

^*b*^SBG31 is a solid-state database computed with periodic boundary conditions; all other databases in this article are atomic and/or molecular databases.

^*c*^The S492 database consists of the S66x8 database from the literature, minus the 36 data used in database S6x6.

One of the databases in Table 2, namely EE69,^36^ was used to test the performance of functionals on electronic excitation energies. This database contains the experimental vertical excitation energies of 30 valence and 39 Rydberg excitations of 11 organic molecules, namely acetaldehyde, acetone, ethylene, formaldehyde, isobutene, pyrazine, pyridazine, pyridine, pyrimidine, *s*-tetrazine, and *s-trans*-butadiene.

## 3. Computational details

All the calculations are performed by a locally modified version^37^ of the *Gaussian 09* program.^38^ Grids, basis sets, and geometries are given in the ESI.†

Vertical electronic excitation energies of the molecules in the EE69 database were calculated by linear-response time-dependent density functional theory (TDDFT).^39^ Twenty excitation energies were computed for each molecule. To compare the computed values with experiment, we made the assignments based on state symmetry as used and discussed in previous work.^40^–^42^

## 4. Design and parameterization of the MN15 functional

The LSDA exchange–correlation energy is a starting point for the MN15 functional. It can be written as:1
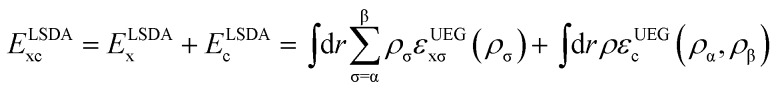
where *E*LSDAx is the LSDA exchange energy, *E*LSDAc is the LSDA correlation energy, *ε*UEGxσ is the exchange energy per electron with spin σ, and *ε*UEGc is the correlation energy per electron for which we use the parameterization of Perdew and Wang.^13^ The MN15 functional is2
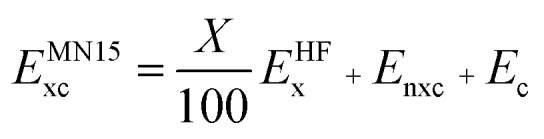
where *E*HFx is the nonlocal Hartree–Fock exchange energy computed from the Kohn–Sham orbitals, *X* is the percentage of Hartree–Fock exchange, *E*_nxc_ is the nonseparable local exchange–correlation energy, and *E*_c_ is the additional correlation energy. The local terms are given by3
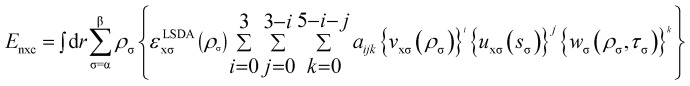
where the variables *v*_xσ_, *u*_xσ_, and *w*_σ_ are the same functions as we used in the MN15-L functional,^29^ and4
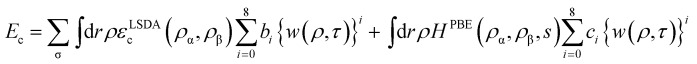
where *w* is a function defined in a previous paper,^32^ and *H*^PBE^ is the PBE gradient correction^43^ for the correlation energy. Eqn (3) is the same form as used in the MN12-L functional,^28^ and eqn (4) is the same as introduced^32^ for M08-HX except that the upper limits of sums have been reduced to 8. We optimized the value of *X* along with the parameters *a*_*ijk*_, *b*_*i*_, and *c*_*i*_ in eqn (3) and (4) by minimizing5
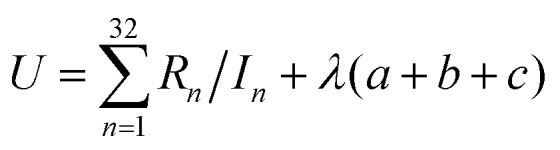
where *R*_*n*_ and *I*_*n*_ are respectively the root mean squared error and inverse weight of database or subset *n* in Table 1. Each term in the sum of eqn (5) corresponds to one of the 32 rows of the table that has an entry in the inverse weight column. The product of *λ* and (*a* + *b* + *c*) is a smoothness restraint, which has the same form as used previously.^26^,^29^ In particular, we have6
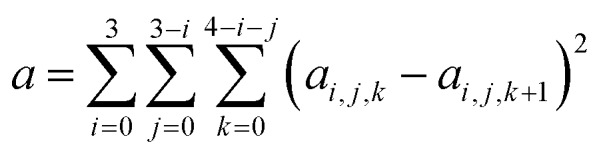

7
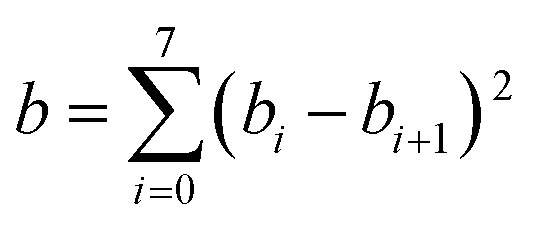

8
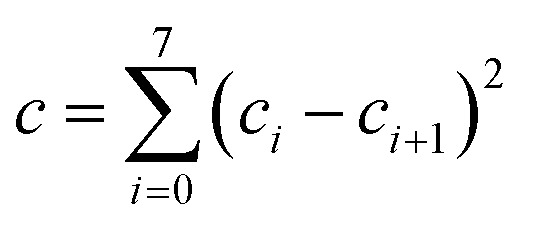



We choose the same value, namely 0.01, of *λ* as we used in the MN15-L parametrization.^29^ All the optimized parameters are shown in Table 3.

**Table 3:** Optimized parameters of the MN15 functional

Exchange				Correlation		*X* ^*a*^
*a*_000_	0.073852235	*a*_102_	6.89391326	*b*_0_	1.093250748	44
*a*_001_	–0.839976156	*a*_103_	2.489813993	*b*_1_	–0.269735037	
*a*_002_	–3.082660125	*a*_104_	1.454724691	*b*_2_	6.368997613	
*a*_003_	–1.02881285	*a*_110_	–5.054324071	*b*_3_	–0.245337101	
*a*_004_	–0.811697255	*a*_111_	2.35273334	*b*_4_	–1.587103441	
*a*_005_	–0.063404387	*a*_112_	1.299104132	*b*_5_	0.124698862	
*a*_010_	2.54805518	*a*_113_	1.203168217	*b*_6_	1.605819855	
*a*_011_	–5.031578906	*a*_120_	0.121595877	*b*_7_	0.466206031	
*a*_012_	0.31702159	*a*_121_	8.048348238	*b*_8_	3.484978654	
*a*_013_	2.981868205	*a*_122_	21.91203659			
*a*_014_	–0.749503735	*a*_200_	–1.852335832	*c*_0_	1.427424993	
*a*_020_	0.231825661	*a*_201_	–3.4722735	*c*_1_	–3.57883682	
*a*_021_	1.261961411	*a*_202_	–1.564591493	*c*_2_	7.398727547	
*a*_022_	1.665920815	*a*_203_	–2.29578769	*c*_3_	3.927810559	
*a*_023_	7.483304941	*a*_210_	3.666482991	*c*_4_	2.789804639	
*a*_030_	–2.544245723	*a*_211_	10.87074639	*c*_5_	4.988320462	
*a*_031_	1.384720031	*a*_212_	9.696691388	*c*_6_	3.079464318	
*a*_032_	6.902569885	*a*_300_	0.630701064	*c*_7_	3.521636859	
*a*_100_	1.657399451	*a*_301_	–0.505825216	*c*_8_	4.769671992	
*a*_101_	2.98526709	*a*_302_	–3.562354535			

^*a*^ *X* denotes the percentage of Hartree–Fock exchange.

The final functional is not overly sensitive to the relative number of data in each subdatabase because the inverse weights are changed manually (by trial and error) to try to obtain balanced performance across the full collection of data.

## 5. Density functionals for comparison

In order to evaluate the performance of the new MN15 functional, we compare the results obtained with the MN15 functional to those obtained with 82 previously developed density functional approximations for the entire Database 2015B. The results for 48 of the previous functionals are compared to the results for the new MN15 functional in the article proper, and the results for the remaining 34 functionals are in ESI.† The 49 functionals considered in the article proper are sorted into types as follows:

• LSDA: GKSVWN5.^7^,^44^,^45^

• GGA: SOGGA,^46^ PBEsol,^47^ SOGGA11,^48^ BP86,^49^,^50^ BLYP,^49^,^51^ PW91,^52^ BPW91,^49^,^52^ PBE,^43^ mPWPW,^53^ revPBE,^54^ RPBE,^55^ HCTH407,^56^ OLYP,^51^,^57^ and OreLYP.^51^,^57^,^58^

• NGA: N12^18^ and GAM.^26^

• meta-GGA: VSXC,^59^ τ-HCTC,^60^ TPSS,^61^ M06-L,^27^ revTPSS,^62^ M11-L,^63^ and MGGA_MS2.^64^

• meta-NGA: MN12-L^19^ and MN15-L.^29^

• Global-hybrid GGA: B3LYP,^49^,^51^,^65^ PBE0,^66^ B98,^67^ B97-1,^68^ O3LYP,^69^ B97-3,^70^ and SOGGA11-X.^71^

• Range-separated hybrid GGA: CAM-B3LYP,^72^ LC-ωPBE,^73^–^76^ HSE06,^77^,^78^ ωB97,^79^ and ωB97X.^79^

• Range-separated hybrid GGA plus molecular mechanics (also called empirical dispersion correction): ωB97X-D.^80^

• Global-hybrid meta-GGA: TPSSh,^81^ τ-HCTHhyb,^82^ BB1K,^14^,^17^,^83^ BMK,^84^ PW6B95,^30^ M06,^31^ M06-2X,^31^ and M08-HX.^32^

• Global-hybrid meta-NGA: MN15.

• Range-separated hybrid meta-GGA: M11.^85^

For each of the functionals in the above list, Table 4 shows the percentage of nonlocal Hartree–Fock exchange, the year in which the functional was published, and the original reference or references.

**Table 4:** Exchange–correlation functionals tested against Database 2015B in the article proper

Category	Type	*X* ^*a*^	Year	Method	Ref.
Local	LSDA	0	1980	GKSVWN5^*b*^	7, 44, 45
	GGA – exchange correct to 2nd order	0	2008	SOGGA	46
		0	2008	PBEsol	47
		0	2011	SOGGA11	48
	GGA – other	0	1988	BP86	14, 50
		0	1988	BLYP	14, 51
		0	1991	PW91^*c*^	52
		0	1991	BPW91	14, 52
		0	1996	PBE	43
		0	1997	mPWPW	53
		0	1997	revPBE	54
		0	1999	RPBE	55
		0	2000	HCTH407	56
		0	2001	OLYP	51, 57
		0	2009	OreLYP	51, 57, 58
	NGA	0	2012	N12	18
		0	2015	GAM	26
	meta-GGA	0	1998	VSXC	59
		0	2002	τ-HCTH	60
		0	2003	TPSS	61
		0	2006	M06-L	27
		0	2009	revTPSS	62
		0	2011	M11-L	63
		0	2013	MGGA_MS2	64
	meta-NGA	0	2012	MN12-L	19
		0	2015	MN15-L	29
Nonlocal	Global-hybrid GGA	20	1994	B3LYP	49, 51, 65
		25	1996	PBE0	66
		21.98	1998	B98	67
		21	1998	B97-1	68
		11.61	2001	O3LYP	69
		26.93	2005	B97-3	70
		35.42	2011	SOGGA11-X	71
	Range-separated hybrid GGA	19–65	2004	CAM-B3LYP	72
		0–100	2006	LC-ωPBE	73–76
		0–25	2006	HSE06	77, 78
		0–100	2008	ωB97	79
		15.77–100	2008	ωB97X	79
	Range-separated hybrid GGA + MM^*d*^	22.2–100	2008	ωB97X-D	80
	Global-hybrid meta-GGA	10	2002	TPSSh	81
		15	2002	τ-HCTHhyb	82
		42	2004	BB1K	14, 17, 83
		42	2004	BMK	84
		28	2005	PW6B95	30
		27	2006	M06	31
		54	2006	M06-2X	31
		52.23	2008	M08-HX	32
	Global-hybrid meta-NGA	44	2015	MN15	Present
	Range-separated hybrid meta-GGA	42.8–100	2011	M11	85

^*a*^ *X* is the percentage of nonlocal Hartree–Fock exchange. When a range is given, the first value is for small interelectronic distances, and the second value is for large interelectronic distances. Details of the functional form that joins these regions of interelectronic separation are given in the references.

^*b*^GVWN5 denotes the Gáspár approximation for exchange and the VWN5 fit to the correlation energy; this is an example of the local spin density approximation (LSDA), and it has the keyword SVWN5 in the *Gaussian 09* program. Note that Kohn–Sham exchange is the same as Gáspár exchange, but Slater exchange (not tested here) is greater by a factor of 1.5.

^*c*^PW91 formally satisfies the gradient expansion for exchange to second order but only at such small values of the gradient that for practical purposes it should be grouped with functionals that do not satisfy the gradient expansion to second order.

^*d*^MM denotes molecular mechanics (also called empirical dispersion correction), which in this case corresponds to atom–atom pairwise damped dispersion terms added post-SCF to the calculated energy.

We do not consider the more expensive doubly hybrid functionals (functionals with nonlocal correlation) in this article, except briefly in Sections 6.2 and 6.4.

## 6. Performance of the MN15 functional

### 6.1. Performance for database 2015B

In order to compare the functionals for different categories of data, we used the following combinations of the subdatabases that are explained more fully in Table 1:

 

• MGBE150: 150 main-group bond energies, in particular SR-MGM-BE9, SR-MGN-BE107, MR-MGM-BE4, MR-MGN-BE17, and ABDE13.

• TMBE33: 33 transition-metal bond energies, in particular SR-TM-BE17, MR-TM-BE13, and MR-TMD-BE3.

• BH76: 76 reaction barrier heights, in particular HTBH38 and NHTBH38.

• EE18: 18 excitation energies, in particular 3dEE8, 4dAEE5, and pEE5.

• IsoE14: 14 isomerization energies, in particular IsoL6, 4pIsoE4, and 2pIsoE4.

• HCTC20: 20 hydrocarbon thermochemical data, in particular πTC13 and HC7.

• MS10: 10 molecular structures, in particular DGL6 and DGH4.

• AME454xAE: 454 atomic and molecular energies, in particular AME471 without AE17 (which denotes 17 absolute energies from H to Cl).

• NC87: 87 noncovalent interactions, in particular the combination of NCCE30, NGDWI21, and S6x6.

 

Another way to classify some of the data is into single-reference and multi-reference systems (these terms are explained in the introduction). Database 2015B has 481 data: 10 structural data (MS10) and 471 energetic data (AME471). If we exclude 87 noncovalent interactions (NC87) and 17 absolute energies (AE17) from AME471, we have 367 energetic data that can be classified as either single-reference data or multi-reference data. We classified these based on the generalized B1 diagnostic,^86^–^88^ which separates these data into 54 multi-reference data (subdatabase MR54) and 313 single-reference data (subdatabase SR313). The numbers of multi-reference systems and single-reference systems in each database are shown in Table 5. Table 5 shows that the MR54 subset contains 13 transition-metal data from MR-TM-BE13 but also a significant amount of other data, including main-group bond energies, reaction barrier heights (which do not contain transition-metals), hydrocarbon systems, *etc.* Therefore, the MR54 subset includes a variety of kinds of systems.

**Table 5:** Multi-reference systems and single-reference systems in Database 2015B^*a*^

Name	Number of multi-reference systems	Number of single-reference systems
SR-MGM-BE9	0	9
SR-MGN-BE120	0	120
MR-MGM-BE4	4	0
MR-MGN-BE17	17	0
SR-TM-BE17	0	17
MR-TM-BE13	13	0
MR-TMD-BE3	3	0
HTBH38/08	1	37
NHTBH38/08	3	35
NCCE30	0	0
S6x6	0	0
NGDWI21	0	0
3dEE8	0	8
4dAEE5	0	5
pEE5	0	5
4pIsoE4	0	4
2pIsoE4	0	4
IsoL6/11	0	6
πTC13	0	13
HC7/11	3	4
EA13/03	0	13
PA8	0	8
IP23	0	23
AE17	0	0
SMAE3	2	1
DC9/12	8	1
Total	54	313

^*a*^The 26 energetic databases from Table 1 are shown here with the number of multi-reference systems and single-reference systems defined by generalized B1 diagnostics.^86^–^88^

The performance of the new MN15 functional and the 48 other functionals in Table 4 has been evaluated for the entire Database 2015B. The results of these tests are given in Tables 6–9 for the energetic part of Database 2015B and in Table 10 for the structural portion of Database 2015B. In addition we compared MN15 against results in the literature for the following additional test sets: the noncovalent interactions of the S66 and S66x8 databases,^34^ the excitation energies of selected organic molecules in the EE69 database,^40^ transition-metal reaction barrier heights in the TMBH21 database,^89^–^91^ transition-metal coordination energies in the WCCR10 database,^92^ semiconductor band gaps in the SBG31 database,^93^ transition-metal dimer bond lengths in the TMBDL7 database,^94^ and geometrical parameters of 47 selected organic molecules.^95^ These evaluations and comparisons are discussed in the following subsections.

**Table 6:** MUE (kcal mol^–1^) for the AME471 database and its subdatabases: LSDA and other gradient approximations

Type	LSDA	GGA														NGA	
Functional	GKSVWN5	SOGGA	PBEsol	SOGGA11	BP86	BLYP	PW91	BPW91	PBE	mPWPW	revPBE	RPBE	HCTH407	OLYP	OreLYP	N12	**GAM**
MGBE150^*a*^	18.36	8.63	8.81	4.01	5.55	4.34	5.04	4.23	4.96	4.47	4.25	4.53	3.80	3.75	3.73	3.42	**2.98**
TMBE33^*b*^	25.20	15.00	14.50	14.00	9.05	9.85	10.26	9.19	9.33	8.82	7.64	7.34	13.31	8.42	6.48	9.36	**5.85**
BH76^*c*^	14.99	11.28	11.28	5.45	8.94	8.03	9.20	7.32	8.87	8.23	6.70	6.63	5.89	5.44	5.93	6.90	**5.25**
NC87^*d*^	2.48	1.61	1.63	2.29	2.43	2.78	1.49	3.07	1.61	2.15	3.04	2.83	2.41	4.32	4.53	2.30	**1.08**
EE18^*e*^	10.81	8.08	8.81	9.63	7.32	8.31	7.41	9.31	7.06	7.80	7.15	6.74	9.80	7.56	8.18	16.22	**6.91**
IsoE14^*f*^	2.34	1.88	1.80	2.17	2.71	4.30	2.38	2.69	2.32	2.55	2.85	2.96	3.55	3.22	3.15	2.21	**3.29**
HCTC20^*g*^	10.63	8.90	7.39	7.01	7.29	13.53	5.32	8.37	5.02	6.99	9.43	9.92	10.59	11.32	10.44	7.09	**7.77**
AME454xAE^*h*^	13.59	7.90	7.88	5.39	6.13	5.92	5.70	5.47	5.49	5.43	5.26	5.33	5.57	5.25	5.18	5.05	**4.14**
AME471^*h*^	28.30	17.83	16.47	5.56	6.52	6.02	5.66	5.70	7.00	5.68	5.47	5.48	5.98	5.43	5.08	5.38	**4.36**
MR54	35.04	22.32	21.92	13.51	14.06	14.08	14.25	12.66	14.15	13.32	11.44	11.58	14.01	11.03	10.22	9.35	**9.45**
SR313	13.03	7.22	7.25	4.96	5.86	5.46	5.46	5.03	5.14	5.05	4.88	5.01	5.06	4.59	4.56	5.20	**4.11**

^*a*^The MGBE150 database consists of SR-MGM-BE9, SR-MGN-BE107, MR-MGM-BE4, MR-MGN-BE17, and ABDE13.

^*b*^The TMBE33 database consists of SR-TM-BE17, MR-TM-BE13, and MR-TMD-BE3.

^*c*^The BH76 database consists of HTBH38/08 and NHTBH38/08.

^*d*^The NC87 database consists of NGDWI21, S6x6, NCCE23 and CT7.

^*e*^The EE18 database consists of 3dEE8, 4dAEE5, and pEE5.

^*f*^The IsoE14 consists of 2pIsoE4, 4pIsoE4, and IsoL6/11.

^*g*^The HCTC20 subdatabase consists of HC7/11 and πTC13.

^*h*^The AME471 database consists all the 27 subdatabases (from 1 to 27 in Table 1) and the AME454xAE consists all the subdatabases except AE17.

**Table 7:** MUE (kcal mol^–1^) for the AME471 database and its subdatabases: meta-GGAs, MN12-L, MN15-L, and global-hybrid GGAs

Type	meta-GGA							meta-NGA		Hybrid						
Functional	VSXC	τ-HCTH	TPSS	M06-L	revTPSS	M11-L	MGGA_MS2	MN12-L	**MN15-L**	B3LYP	PBE0	B98	B97-1	O3LYP	B97-3	SOGGA11-X
MGBE150^*a*^	3.02	3.40	3.49	2.63	3.26	2.81	3.93	2.35	**1.84**	3.58	2.82	2.71	2.09	3.51	2.50	2.62
TMBE33^*b*^	7.77	7.83	7.11	5.29	7.36	7.02	7.92	11.20	**5.16**	8.83	10.18	6.92	5.04	10.49	9.85	15.26
BH76^*c*^	4.91	6.39	8.31	3.99	8.02	2.15	6.22	1.78	**1.66**	4.39	3.83	3.74	3.89	3.85	1.83	1.48
NC87^*d*^	4.23	2.22	1.92	0.57	1.83	0.97	1.11	0.74	**0.98**	2.05	1.33	1.51	1.32	3.61	2.10	1.50
EE18^*e*^	7.63	14.02	6.96	8.37	6.71	14.44	10.77	18.65	**3.71**	6.47	6.85	7.16	7.31	6.04	6.20	5.14
IsoE14^*f*^	4.27	3.52	3.32	2.91	3.35	3.06	2.75	2.17	**2.19**	3.67	1.87	2.55	2.33	2.98	2.56	2.22
HCTC20^*g*^	10.56	10.71	8.95	5.52	7.35	4.19	10.38	4.36	**4.54**	9.80	7.26	7.60	6.76	9.58	7.44	6.50
AME454xAE^*h*^	4.71	5.00	4.88	3.28	4.70	3.45	4.96	3.52	**2.19**	4.45	3.72	3.50	3.07	4.73	3.44	3.58
AME471^*h*^	6.34	5.44	5.35	3.42	5.39	4.11	5.36	3.75	**2.36**	4.95	4.98	3.55	3.15	4.76	3.57	3.63
MR54	8.66	9.37	9.48	5.91	9.39	6.74	10.76	8.93	**4.35**	10.67	10.37	8.26	6.09	10.19	9.92	12.84
SR313	4.23	5.10	4.96	3.61	4.74	3.71	5.07	3.49	**2.15**	4.09	3.28	3.28	3.08	4.13	2.70	2.56

^*a*^The MGBE150 database consists of SR-MGM-BE9, SR-MGN-BE107, MR-MGM-BE4, MR-MGN-BE17, and ABDE13.

^*b*^The TMBE33 database consists of SR-TM-BE17, MR-TM-BE13, and MR-TMD-BE3.

^*c*^The BH76 database consists of HTBH38/08 and NHTBH38/08.

^*d*^The NC87 database consists of NGDWI21, S6x6, NCCE23 and CT7.

^*e*^The EE18 database consists of 3dEE8, 4dAEE5, and pEE5.

^*f*^The IsoE14 consists of 2pIsoE4, 4pIsoE4, and IsoL6/11.

^*g*^The HCTC20 subdatabase consists of HC7/11 and πTC13.

^*h*^The AME471 database consists all the 27 subdatabases (from 1 to 27 in Table 1) and the AME454xAE consists all the subdatabases except AE17.

**Table 8:** MUE (kcal mol^–1^) for the AME471 database and its subdatabases: range-separated-hybrid GGAs, global-hybrid meta-GGAs, MN15

Type	RSH GGA					RSH GGA + MM	GH mGGA								RSH mGGA	**GH mNGA**
Functional	CAM-B3LYP	LC-ωPBE	HSE06	ωB97	ωB97X	ωB97X-D	TPSSh	τ-HCTHhyb	BB1K	BMK	PW6B95	M06	M06-2X	M08-HX	M11	**MN15**
MGBE150^*a*^	3.09	3.47	2.97	2.46	2.42	2.23	3.76	2.37	3.63	2.06	2.65	1.90	1.87	2.74	2.36	**1.36**
TMBE33^*b*^	10.75	12.43	9.92	10.31	10.57	8.70	6.78	5.62	16.20	13.02	9.50	7.10	19.11	17.59	14.36	**5.54**
BH76^*c*^	2.90	1.77	3.98	2.15	2.45	3.05	6.39	4.88	1.30	1.21	2.98	2.16	1.18	0.97	1.29	**1.36**
NC87^*d*^	1.35	1.56	1.31	0.55	0.64	0.30	1.92	1.61	1.56	1.68	1.03	0.67	0.35	0.37	0.39	**0.25**
EE18^*e*^	6.20	8.46	8.09	11.51	8.94	8.30	6.46	9.00	6.58	6.74	5.34	8.45	7.86	5.78	8.99	**6.28**
IsoE14^*f*^	2.89	1.55	1.99	1.42	1.79	1.75	3.02	2.50	1.68	1.54	2.01	1.66	1.94	1.26	1.74	**1.41**
HCTC20^*g*^	4.57	8.96	6.60	6.58	5.21	5.68	7.65	7.25	7.23	5.09	5.24	3.83	1.72	2.93	2.77	**3.59**
AME454xAE^*h*^	3.59	4.02	3.85	3.39	3.20	2.99	4.52	3.57	4.08	3.07	3.21	2.59	3.11	3.25	3.20	**1.88**
AME471^*h*^	3.85	4.79	4.89	3.49	3.29	3.09	4.91	3.66	4.49	3.57	6.65	2.66	3.08	3.28	3.41	**2.08**
MR54	9.64	11.81	10.18	10.26	9.72	8.89	9.41	6.38	15.20	10.03	8.95	5.43	13.33	13.62	10.35	**4.75**
SR313	3.20	3.39	3.54	3.05	2.84	2.77	4.43	3.68	2.85	2.30	2.86	2.71	2.13	2.29	2.80	**1.85**

^*a*^The MGBE150 database consists of SR-MGM-BE9, SR-MGN-BE107, MR-MGM-BE4, MR-MGN-BE17, and ABDE13.

^*b*^The TMBE33 database consists of SR-TM-BE17, MR-TM-BE13, and MR-TMD-BE3.

^*c*^The BH76 database consists of HTBH38/08 and NHTBH38/08.

^*d*^The NC87 database consists of NGDWI21, S6x6, NCCE23 and CT7.

^*e*^The EE18 database consists of 3dEE8, 4dAEE5, and pEE5.

^*f*^The IsoE14 consists of 2pIsoE4, 4pIsoE4, and IsoL6/11.

^*g*^The HCTC20 subdatabase consists of HC7/11 and πTC13.

^*h*^The AME471 database consists all the 27 subdatabases (from 1 to 27 in Table 1) and the AME454xAE consists all the subdatabases except AE17.

**Table 9:** MUE (kcal mol^–1^) of the ten best-performing functionals (out of 83 tested) for the CT7 charge transfer database^*a*^

Name	SOGGA11-X	MPWB1K	MN15-L	MN15	MGGA_MS2	PWB6K	ωB97X-D	M11	M06-HF	M06-2X
CT7	0.21	0.23	0.25	0.25	0.26	0.26	0.28	0.30	0.35	0.37

^*a*^The seven intermolecular charge transfer systems included in the database are C_2_H_4_···F_2_, NH_3_···F_2_, C_2_H_2_···ClF, HCN···ClF, NH_3_···Cl_2_, H_2_O···ClF, and NH_3_···ClF.

**Table 10:** MUE (Å) for the molecular structure 10 Database and its subdatabases

Functional	Type	DGL6	DGH4	MS10^*a*^
**MN15**	**GH mNGA**	**0.005**	**0.008**	**0.006**
N12	NGA	0.008	0.007	0.008
MN15-L	mNGA	0.004	0.014	0.008
PBE0	GH GGA	0.003	0.014	0.008
HSE06	RSH GGA	0.003	0.015	0.008
PBEsol	GGA	0.010	0.007	0.009
CAM-B3LYP	RSH GGA	0.008	0.010	0.009
TPSSh	GH mGGA	0.006	0.013	0.009
PW6B95	GH mGGA	0.004	0.016	0.009
SOGGA	GGA	0.009	0.013	0.010
revTPSS	mGGA	0.011	0.009	0.010
τ-HCTHhyb	GH mGGA	0.006	0.017	0.010
BB1K	GH mGGA	0.009	0.011	0.010
τ-HCTH	mGGA	0.006	0.019	0.011
M06-L	mGGA	0.006	0.018	0.011
M11	RS-hybrid-meta	0.007	0.017	0.011
TPSS	mGGA	0.010	0.015	0.012
MGGA_MS2	mGGA	0.005	0.022	0.012
MN12-L	mGGA	0.005	0.022	0.012
LC-ωPBE	RSH GGA	0.013	0.011	0.012
ωB97X	RSH GGA	0.008	0.017	0.012
ωB97X-D	RSH GGA-D	0.005	0.023	0.012
VSXC	mGGA	0.006	0.022	0.013
M06	GH mGGA	0.006	0.023	0.013
O3LYP	GH GGA	0.004	0.030	0.014
SOGGA11-X	GH GGA	0.004	0.029	0.014
ωB97	RSH GGA	0.011	0.018	0.014
PW91	GGA	0.012	0.019	0.015
HCTH407	GGA	0.004	0.033	0.015
B98	GH GGA	0.007	0.026	0.015
B97-1	GH GGA	0.006	0.028	0.015
BMK	GH mGGA	0.007	0.027	0.015
PBE	GGA	0.013	0.020	0.016
mPWPW	GGA	0.012	0.021	0.016
B3LYP	GH GGA	0.009	0.027	0.016
B97-3	GH GGA	0.004	0.034	0.016
BPW91	GGA	0.013	0.022	0.017
BP86	GGA	0.015	0.021	0.018
GAM	NGA	0.007	0.034	0.018
GKSVWN5	LSDA	0.011	0.031	0.019
OLYP	GGA	0.009	0.036	0.020
OreLYP	GGA	0.011	0.034	0.020
M11-L	mGGA	0.012	0.033	0.021
M06-2X	GH mGGA	0.004	0.049	0.022
M08-HX	GH mGGA	0.005	0.047	0.022
revPBE	GGA	0.015	0.034	0.023
RPBE	GGA	0.016	0.038	0.025
SOGGA11	GGA	0.008	0.053	0.026
BLYP	GGA	0.019	0.037	0.026

^*a*^The MS10 database consists of DGL6 and DGH4 subdatabases. The functionals are listed in the order of increasing value in the last column.

**Fig. 2:**
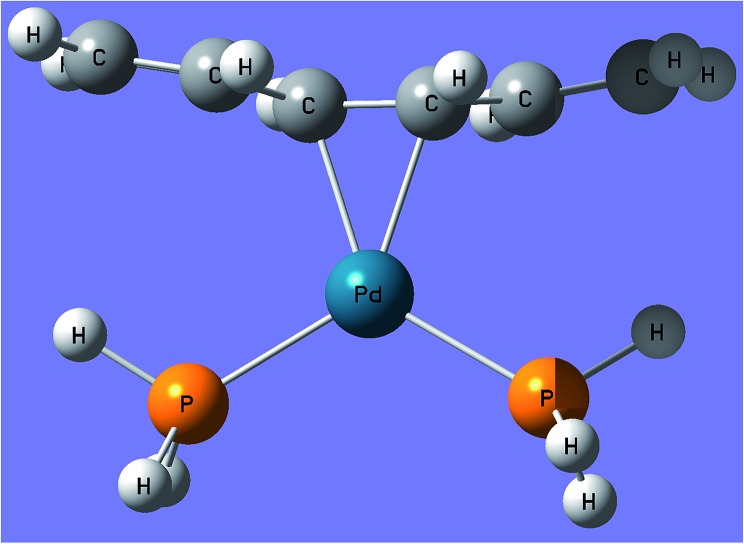
The structure of Pd(PH_3_)_2_C_6_H_8_ in subdatabase PdBE2.

All energetic quantities in this paper are calculated from Born–Oppenheimer energies without including vibrational energies. Therefore when the reference data are from experiment, we removed zero-point vibrational energy and thermal vibration-rotational energies, if present in the original data. For example, bond energies are equilibrium dissociation energies (*D*_e_), not ground-state dissociation energies (*D*_0_). Most of the bond energies in the databases are average bond energies calculated as atomization energy per bond. Even when all bonds are not atomized, we calculate bond energies on a per bond basis. For example one of the bond-breaking processes in subdatabase PdBE2 is the dissociation of Pd(PH_3_)_2_C_6_H_8_ into Pd(PH_3_)_2_ and C_6_H_8_. As shown in Fig. 2, this involves breaking two bonds, so the energy of dissociation is divided by 2. Further details of the molecules in the databases, the bonds broken, and the removal of vibrational energy from experiment are given in ref. 26 and in the references in Tables 1 and 3.

In evaluating performance, we always use the unweighted mean unsigned error (MUE) over the data in a database, subdatabase, or specified collection of subdatabases. Thus, even though the MN15 functional was optimized using root-mean-square errors and weights, performance is consistently measured with unweighted MUEs.

We show the performance of the MN15 functional for AME471 and the 11 combined energetic subdatabases delineated above in Tables 6–8. Table 6 has the performance of a local spin density approximation (LSDA) and 16 gradient approximations (GAs); Table 7 has the performance of nine meta gradient approximations (meta-GAs) and seven global-hybrid gradient approximations (hybrid GAs); and Table 8 has the performance of six range-separated hybrid gradient approximations (RS-hybrid GAs), nine global-hybrid meta gradient approximations (hybrid meta-GAs), and one range-separated-hybrid meta gradient approximation (RS-hybrid meta-GA).

Tables 6–8 show that the MN15 functional gives the best result for main-group bond energies (MGBE150) with an MUE of 1.36 kcal mol^–1^, followed by MN15-L, M06-2X, and M06 with MUEs of 1.84, 1.87, and 1.90 kcal mol^–1^ respectively. The MN15 functional gives the fourth best result for transition-metal bond energies (TMBE33) with an MUE of 5.54 kcal mol^–1^, with B97–1, MN15-L, and M06-L being the top three with MUEs of 5.04, 5.16, and 5.29 kcal mol^–1^, respectively. The other functional that gives an MUE smaller than 6.0 kcal mol^–1^ for this subset of the data is τ-HCTHhyb, with an MUE of 5.62 kcal mol^–1^.

We found that it is hard to get simultaneously good accuracy for certain pairs of databases, even when both are present in the training set. For example, a functional that gives good results for transition-metal bond energies does not usually work very well for reaction barrier heights, and *vice versa*. Incorporating Hartree–Fock exchange usually improves the performance on hydrocarbons, weak interactions, and barrier heights, but it usually worsens the results for multi-reference systems. Therefore, the mathematical form of the functional and a carefully selected set of diverse databases are very important for the design of a universal and highly transferable functional such as MN15. For the reaction barrier heights database (BH76), the top six best functionals are M08-HX, M06-2X, BMK, BB1K, M11, and MN15, which all have an MUE smaller than 1.50 kcal mol^–1^.

The MN15 functional gives the second best results for the isomerization energies database (IsoE14), with an MUE of 1.41 kcal mol^–1^, following only M08-HX with an MUE of 1.26 kcal mol^–1^. The MN15 functional gives the fourth best results for the hydrocarbon thermochemistry database (HCTC20), with an MUE of 3.59 kcal mol^–1^. The top three functionals for HCTC20 are M06-2X with an MUE of 1.72 kcal mol^–1^, M11 with an MUE of 2.77 kcal mol^–1^, and M08-HX with an MUE of 2.93 kcal mol^–1^.

The MN15 functional gives the best results for the noncovalent interactions (NC87), with an MUE of 0.25 kcal mol^–1^, followed by ωB97X-D, M06-2X, M08-HX, M11, ωB97, M06-L, ωB97X, and M06, which have MUEs of 0.30, 0.35, 0.37, 0.39, 0.55, 0.57, 0.64, and 0.67 kcal mol^–1^, respectively.

For the excitation energies database (EE18), the top eight functionals are MN15-L, SOGGA11-X, PW6B95, M08-HX, O3LYP, B97-3, CAM-B3LYP and MN15, which are the only functionals that have MUEs for EE18 smaller than 6.30 kcal mol^–1^. It is especially noteworthy that MN15-L gives an MUE of only 3.71 kcal mol^–1^ and the other five give MUEs in the range 5.00–6.30 kcal mol^–1^.

The MN15 functional is the only one that gives MUEs no larger than 2.10 kcal mol^–1^ for both AME471 with 471 energetic data and AME454xAE with 454 energetic data excluding absolute energies (AE17). For the SR313 database of 313 single-reference data, the top five functionals are MN15, M06-2X, MN15-L, M08-HX, and BMK with MUEs of 1.85 kcal mol^–1^, 2.13 kcal mol^–1^, 2.15 kcal mol^–1^, 2.29 kcal mol^–1^, and 2.30 kcal mol^–1^ respectively. For the MR54 database of 54 multi-reference data, MN15-L and MN15 are the only two functionals that give an MUE smaller than 5.00 kcal mol^–1^, and M06 and M06-L are the only two functionals with MUEs in the range of 5.00 kcal mol^–1^ and 6.00 kcal mol^–1^.

In order to show the performance for intermolecular charge transfer, we found the ten functionals (out of 83 tested) that perform best for the CT7 charge transfer database, and we list their MUEs for this database in Table 9. The MN15-L and MN15 functionals are essentially tied for third best with an MUE of 0.25 kcal mol^–1^. The MN15-L and MGGA_MS2 functionals are the only two local functionals that gives an MUE smaller than 0.30 kcal mol^–1^ for this database. The MUEs of the other 73 functionals against CT7 database are shown in the ESI.†

Table 10 shows the performance of 49 functionals including MN15 for the molecular structure database MS10 and its subdatabases DGL6 and DGH4. The MN15 functional is the tenth best for DGL6 with an MUE of 0.005 Å and the third best for DGH4 with an MUE of 0.008 Å. Because it is rare for a functional to do well on both databases, MN15 is the best overall when these databases are combined into MS10. We conclude that the MN15 functional gives good results not only for energies but also for geometries.

Some functionals that are good for one property (such as main-group bond energies, transition-metal bond energies, noncovalent interactions, *etc.*), as seen for certain databases in Tables 6–10, fail badly for other properties. Parameterizing a functional that is good for several properties is the challenge of designing and parameterizing a universal functional. Average errors over diverse databases such as ME471 are one way to gauge the universality of a functional, but such mean errors are sensitive to the number of data of each type and can underweight performance in categories with intrinsically smaller errors (such as noncovalent interactions) unless those categories have a compensatingly large number of data, but the amount of compensation is arbitrary.

A better measure, at least for relative success compared to other functionals, is to look at the ranking of the mean unsigned error in each category. To illustrate the high universality of the MN15 functional, we consider Table 11, which lists the rankings for 28 subdatabases of 12 selected functionals among the set of 83 functionals that were tested for the entire Database 2015B. The table also shows the average ranking, which is a way to summarize combined performance on diverse databases that does not suffer from the two deficiencies mentioned at the start of this paragraph, and it also shows the lowest ranking. The MN15 functional gives the best average ranking, which is 9, with MN15-L being second with an average ranking of 17. The average ranking of the other functionals is in the range of 22–52. Furthermore, every other functional in the list has at least one ranking of 62 or lower, whereas MN15 ranks lower than 29th in none of the 28 categories. MN15-L ranks lower than 29th in 4 categories, M06, M06-2X, and ωB97X-D rank lower than 29th in 9–11 categories, M06-L ranks lower than 29th in 14 categories, and the other functionals in the table rank lower than 29th in 19–23 categories.

**Table 11:** The rankings (out of 83 functionals) of 12 selected functionals for 28 atomic and molecular databases

Name	BP86	PBE	B3LYP	TPSS	HSE06	M06-L	τ-HCTHhyb	ωB97X-D	M06-2X	M06	MN15-L	MN15
SR-MGM-BE9	24	18	58	16	39	35	10	12	3	37	19	**29**
SR-MGN-BE107	75	69	48	46	33	29	20	10	2	8	12	**1**
SR-TM-BE17	52	49	20	22	11	37	38	3	58	18	2	**6**
MR-MGM-BE4	48	46	23	13	33	7	4	49	57	3	2	**1**
MR-MGN-BE17	73	75	30	13	34	3	15	44	37	10	1	**2**
MR-TM-BE13	61	64	15	43	25	20	2	8	69	5	11	**7**
IsoL6/11	51	44	57	75	10	61	32	5	20	11	12	**28**
IP23	75	63	55	38	32	29	27	7	15	52	3	**2**
EA13/03	72	27	30	31	45	67	6	9	19	8	22	**2**
PA8	21	17	2	66	8	44	47	61	35	40	55	**9**
πTC13	33	27	37	67	44	49	61	45	1	16	20	**9**
HTBH38/08	76	77	39	70	40	37	47	25	5	21	8	**3**
NHTBH38/08	72	70	43	75	37	39	42	38	3	21	15	**12**
NCCE30	65	57	46	56	33	19	39	7	2	11	22	**9**
AE17	57	73	60	59	70	23	17	16	1	5	22	**19**
ABDE13	40	35	45	55	33	36	31	10	9	19	30	**15**
HC7/11	48	11	68	49	37	8	35	19	1	6	12	**7**
3dEE8	47	32	20	44	54	27	66	18	17	41	2	**1**
4dAEE5	24	13	36	28	25	51	63	58	70	62	1	**3**
pEE5	19	31	11	6	56	66	29	69	40	50	42	**28**
DC9/12	62	60	46	54	33	40	36	20	8	3	9	**1**
2pIsoE4	47	36	74	58	31	43	48	18	16	13	20	**1**
4pIsoE4	50	27	75	37	38	51	48	29	40	23	68	**19**
S6x6	39	23	34	33	19	6	29	1	4	9	13	**2**
NGDWI21	80	16	66	42	18	26	44	39	24	52	3	**2**
MR-TMD-BE3	17	31	50	12	59	1	13	56	75	46	23	**26**
SMAE3	67	65	62	45	49	21	15	24	35	1	6	**4**
MS10	54	46	50	29	9	2	18	33	66	35	7	**1**
Lowest	80	77	75	75	70	67	66	69	75	62	68	**29**
Average	52	43	43	42	34	31	32	26	26	22	17	**9**

### 6.2. Performance for S66 and S66x8 databases

The S66x8 and S66 databases^34^ were developed primarily for the validation of new quantum chemical methods. The S66 database contains accurate interaction energies for 66 noncovalently bound complexes at the equilibrium van der Waals geometry of the complex, and it is divided into three sub-databases: complexes that are damped-dispersion-dominated (DD23), hydrogen-bonding complexes (HB23), and complexes dominated by a mix of damped dispersion and electrostatics (Mix20). DD23 contains stacked and unstacked aromatic complexes and complexes containing aliphatic molecules; HB23 contains both singly H-bonded and doubly H-bonded complexes. Examples of mixed systems are benzene–water and pentane–acetic acid. The S66x8 database contains not only S66 as a subset but also interaction energies of the 66 complexes at seven other intersubsystem distances, ranging from 0.9 times the van der Waals distance to 2.0 times the van der Waals distance. The average interaction energy in S66 is 5.46 kcal mol^–1^, and the average interaction energy in S66x8 is 4.15 kcal mol^–1^.

Goerigk *et al.*^96^ choose to benchmark several methods against the S66 and S66x8 databases, choosing methods based mainly on their good performance relative to other functionals of the same class in previous tests, but also including three functionals based on their widespread use or general interest. In Tables 12 and 13 we compare the performance of MN15 to the performance of the functionals they chose and also to ωB97X-D, which is not in their paper but is added to the comparison here. The functionals they chose include functionals with nonlocal correlation terms, which are more expensive but often favored for treating noncovalent complexes because dispersion interactions in the long-range region where the subsystems have no overlap can only be treated by nonlocal correlation.

**Table 12:** The performance (kcal mol^–1^) of selected density functionals for the S60 and S492 databases and subdatabases^*a*^

DFT	DD21^*b*^	HB20^*c*^	Mix19^*d*^	S60	S492
Without nonlocal correlation or molecular mechanics (also called empirical dispersion correction)					
**MN15**	**0.59**	**0.39**	**0.31**	**0.43**	**0.32**
M06-2X	0.33	0.27	0.23	0.26	0.34
M05-2X	1.04	0.09	0.42	0.56	0.46
M06-L	0.68	0.26	0.74	0.54	0.48
PW6B95	2.43	0.59	1.41	1.57	1.06
MPW1B95	2.82	0.70	1.64	1.83	1.24
PBE	3.73	0.05	2.00	2.05	1.41
LC-ωPBE	3.95	0.71	2.18	2.43	1.69
TPSS	4.95	0.46	2.83	2.95	1.98
B3LYP	5.30	0.50	3.08	3.15	2.25
BLYP	6.26	1.14	3.82	3.99	2.80
revPBE	6.42	2.02	3.90	4.42	2.91

^*a*^Results for the full databases (DA23, HB23, Mix20, S66, and S66x8) are in the next table. This table includes only functionals without nonlocal correlation and without empirical molecular mechanics correction terms.

^*b*^DD21 is the dispersion-dominated subdatabase.

^*c*^HB20 is the hydrogen bonding subdatabase.

^*d*^Mix19 is the mixed subdatabase.

**Table 13:** The performance (kcal mol^–1^) of selected density functionals for the S66 and S66x8 databases and subdatabases

DFT	DD23^*a*^	HB23^*b*^	Mix20^*c*^	S66	S66x8
Without nonlocal correlation or molecular mechanics (also called empirical dispersion correction)					
**MN15**	**0.57**	**0.36**	**0.32**	**0.42**	**0.32**
M06-2X	0.35	0.24	0.25	0.28	0.34
M05-2X	1.03	0.27	0.43	0.58	0.49
M06-L	0.69	0.39	0.73	0.60	0.51
PW6B95	2.39	0.99	1.40	1.60	1.13
MPW1B95	2.76	1.13	1.62	1.85	1.31
PBE	3.63	0.74	1.94	2.11	1.51
LC-ωPBE	3.85	1.35	2.14	2.46	1.78
TPSS	4.83	1.38	2.77	3.00	2.11
B3LYP	5.17	1.47	3.00	3.22	2.37
oTPSS	5.99	2.17	3.55	3.92	2.72
BLYP	6.15	2.22	3.77	4.06	2.97
revPBE	6.35	3.03	3.91	4.45	3.13

With nonlocal correlation					
B2GP-PLYP	1.88	0.32	1.01	1.07	0.81
PWPB95	1.81	0.95	1.09	1.29	0.92
B2-PLYP	2.66	0.61	1.51	1.60	1.19

With molecular mechanics (also called empirical dispersion correction) added					
PW6B95-D3(BJ)	0.16	0.23	0.15	0.18	0.21
ωB97X-D	0.47	0.28	0.22	0.32	0.23

With nonlocal correlation and molecular mechanics (also called empirical dispersion correction)					
DSD-BLYP-D3	0.23	0.36	0.14	0.21	0.16

^*a*^DD23 is the dispersion-dominated subdatabase.

^*b*^HB23 is the hydrogen bonding subdatabase.

^*c*^Mix20 is the mixed subdatabase.

S66x8 has 528 data and S66 has 66 data; of these we used only 36 (which is 7%) of the data of S66x8 for training MN15, and we used only six of the data in S66 for training. These subsets are called S6x6 (six systems at 0.90, 0.95, 1.00, 1.05, 1.10, and 1.25 times the equilibrium distances) and S6 (six systems at the equilibrium distances) respectively. The remaining data after excluding the 36 data from the 528 data in S66x8 form a test that was called S492 in Table 2. The remaining data after excluding the 6 data from the 66 data in S66 will be called test set S60. Similarly the data remaining after excluding training data from DD23, HB23, and Mix20 constitute test sets DD21, HB20, and Mix19. Table 12 shows comparisons for the test-only sets and Table 13 shows comparison for the full data sets.

Before considering Tables 12 and 13 in more detail, we provide background contextual comments on dispersion. Dispersion interactions were originally modeled by London, and his formulas apply only at long range where the subsystems have no charge cloud penetration.^97^ In the strict meaning of the term, “dispersion force” applies only in such regions. As subsystems begin to overlap there is no unique way to partition the interaction energy into dispersion and the remainder, although there are reasonable ways to do so in an approximate sense.^98^ However, when doing this, the dispersion component of the interaction energy no longer satisfies the inverse power laws of long-range dispersion; rather it is damped by overlap. This overlap also leads to Pauli repulsion, and when the intersubsystem distance is reduced to the van der Waals distance, since that represents equilibrium geometry, the repulsive force exactly cancels the attractive force (and therefore is not negligible). Functionals with local correlation, such as MN15 and the other functionals to which we compare in the other sections of this article, cannot treat the inverse power dependence of long-range dispersion, but in principle they can treat attractive noncovalent interactions at the van der Waals geometry, as tested by S66 and its subdatabases, and in principle they can even provide reasonable results at larger distances in the range tested by S66x8. Table 12 tests how well this is done by various functionals.

In Table 12 we show the performance of density functionals without nonlocal correlation or molecular mechanics (also called empirical dispersion correction) against data that are only used for testing. In Table 13 we show the results for the whole S66x8 database including the six molecular systems we included in training. We have classified functionals in Table 13 based on whether or not they contain nonlocal correlation or damped-dispersion molecular mechanics terms or both. Note that Table 13 includes results for the oTPSS^99^ functional which is not among 83 functionals in Table 4 and in the ESI†. Although the table includes all the functionals without molecular mechanics that were selected by the authors of ref. 96, it includes only two of their examples including molecular mechanics (the best for S66 with local correlation and one with nonlocal correlation that was specifically designed for use with molecular mechanics terms), since molecular mechanics corrections are not the focus of our article. For greater completeness we also include our results for ωB97X-D. There are four doubly hybrid functionals included in Table 13, in particular B2-LYP,^100^ B2GP-PLYP,^101^ DSD-BLYP-D3,^96^,^102^ and PWPB95.^103^

Table 12 shows that the MN15 functional gives the best results of any functional in the table for the S492 database, even though none of this data was used for training and even though the other functionals in the table were selected^96^ mainly for their good performance on this kind of problem. The MN15 functional gives the second best results for the dispersion-dominated (DD23), mixed (Mix19) and S60 databases, with M06-2X being the best. The MN15 functional gives the fourth best results for hydrogen bond (HB20) database with M06-2X, M05-2X and M06-L being the first, second, and third. The MN15 functional performs nearly the same in both Tables 12 and 13.

It is especially remarkable that MN15 does better than all three functionals with nonlocal correlation but without molecular mechanics dispersion in Table 13. Functionals with empirical molecular mechanics terms are the best for noncovalent interactions, but they are sometimes poorly balanced when one consider properties more general than noncovalent interactions. For example, Table 11 shows that the ωB97X-D functional is below average for multi-reference main-group metal bond energies, for multireference main-group non-metal bond energies, for proton affinities, for thermochemistry of π systems, for 4d atomic excitation energies, for p-block excitation energies, and for multireference transition-metal-dimer bond energies.

### 6.3. Performance for excitation energy database

Because of its modest computational cost and often-useful accuracy, TDDFT is now the workhorse for calculating energies and properties of electronically excited states of large molecules. However, one of the most vexing problems of TDDFT with functionals in the literature is the difficulty of achieving high accuracy for valence and Rydberg excitations with the same functional.^40^ Local functionals are well-known to underestimate Rydberg excitations severely,^104^ causing serious problems in applications to spectroscopy where Rydberg states are intermixed with valence states and in applications to photochemistry where states with Rydberg character play an important role in many photodynamical processes; for example, the repulsive *n*σ* state important for the photodissociation of thioanisole has Rydberg character.^105^ Even when Rydberg states are high in energy and unimportant, underestimation of their energy by TDDFT brings them down to the energy range of valence states and damages the calculated spectrum. The problem is fundamental and has to do with the self-interaction error and the consequent incorrect asymptotic behavior of the exchange–correlation potential.^41^,^42^,^104^,^106^,^107^

Methods have been proposed to solve the problem within the conventional Kohn–Sham framework. Hybrid functionals alleviate this problem by incorporating a fraction of the Hartree–Fock exchange, whose derived exchange potential has the correct asymptotic behavior. Although a hybrid functional generally still does not have the correct asymptotic exchange potential unless it has 100% Hartree–Fock exchange, it improves the potential in the middle range of interelectronic separation, which is most important for Rydberg excitations.^42^ However, a high percentage of Hartree–Fock exchange, while helpful for Rydberg excitations, often compromises the accuracy for valence states. A good overall design and balance in the functional, instead of simply raising the fraction of Hartree–Fock exchange, is therefore required to obtain simultaneous accuracy for valence and Rydberg excitations. Range-separated hybrid functionals^73^ are another popular way to achieve a better balance of accuracy for valence and Rydberg excitations. A local exchange enhancement scheme has also been proposed for this purpose.^42^

The general trends discussed in the preceding paragraphs can be seen in Table 14, where we compare the performance of MN15 with 60 other methods reported in ref. 36 and 40 for the calculation of valence and Rydberg excitation energies of selected organic molecules (EE69). Many of the functionals in Table 14 are already defined and referenced in Table 5; references^108^–^121^ for the others are given in the table. The table shows that MN15 gives the lowest mean unsigned error averaged over all states and is the only functional that gives an error smaller than 0.3 eV for both valence and Rydberg states. The functionals that do nearly as well, with an error smaller than 0.4 eV for both types of excitations, include M06-2X, BMK, and M05-2X, which are hybrid functionals with a high percentage (42–56%) of Hartree–Fock exchange, and ωB97X-D and CAM-B3LYP, which are range-separated hybrid functionals with respectively 100 and 65% Hartree–Fock exchange at large interelectronic separation.

**Table 14:** The mean unsigned errors (MUE, in eV) of 60 selected methods for the vertical excitation energies of 30 valence, 39 Rydberg, and all 69 transitions

Name	*X* ^*a*^	Valence	Rydberg	All states	Ref. for data	Ref. for method
**MN15**	44	**0.29**	**0.24**	**0.26**	**Present**	**Present**
EOM-CCSD	WFT	0.47	0.11	0.27	36	108
M06-2X	54	0.36	0.26	0.30	40	31
ωB97X-D	22.2–100	0.32	0.28	0.30	40	80
MPWKCIS1K	41	0.40	0.27	0.32	40	109
PWB6K	46	0.43	0.24	0.32	40	110
CAM-B3LYP	19–65	0.31	0.35	0.33	36	72
MPW1K	42.8	0.45	0.23	0.33	40	111
MPWB1K	44	0.40	0.28	0.33	40	112
ωB97X	15.77–100	0.40	0.28	0.33	40	79
BMK	42	0.33	0.39	0.36	36	84
M05-2X	52	0.37	0.35	0.36	36	113
LC-ωPBE	0–100	0.41	0.32	0.36	36	73–76
B3P86	20	0.19	0.53	0.38	36	49, 50, 65
SOGGA11-X	40.15	0.46	0.34	0.39	40	71
BH&H	50	0.49	0.33	0.40	36	36
ωB97	0–100	0.45	0.39	0.41	40	79
M08-SO	56.79	0.35	0.49	0.43	40	114
BH&HLYP	50	0.56	0.36	0.44	36	36
LC-BLYP	0–100	0.49	0.41	0.45	36	49, 51, 73
M08-HX	52.23	0.38	0.51	0.46	40	32
M11	42.8–100	0.37	0.54	0.47	40	85
CIS(D)	WFT	0.50	0.49	0.49	36	115
M06-HF	100	0.56	0.44	0.49	40	116
PBE0	25	0.22	0.80	0.55	36	66
HSE	25–0	0.21	0.82	0.56	36	117
B3P86(VWN5)	20	0.19	0.87	0.57	36	45, 49, 50, 65
N12-SX	25–0	0.26	0.85	0.59	40	33
M05	26	0.24	0.90	0.62	36	118
LC-HCTH/93	0–100	0.53	0.70	0.63	40	56, 73
B3LYP	20	0.20	1.03	0.67	36	49, 51, 65
τ-HCTHhyb	15	0.18	1.04	0.67	36	82
LC-HCTH/147	0–100	0.53	0.85	0.71	40	56, 73
LC-HCTH/407	0–100	0.53	0.85	0.71	40	56, 73
LC-B97-D	0–100	0.53	0.84	0.71	40	73, 121
M06-L	0	0.28	1.08	0.73	40	27
TPSSh	10	0.18	1.27	0.80	36	81
LC-τ-HCTH	0–100	0.54	1.00	0.80	40	73, 82
LSDA	0	0.45	1.20	0.88	36	7, 44, 45
HCTH/147	0	0.38	1.26	0.88	40	56
M06	27	0.30	1.33	0.88	40	31
O3LYP	11.61	0.20	1.47	0.92	36	69
B97-D	0	0.39	1.35	0.93	40	121
VSXC	0	0.24	1.54	0.97	36	59
HCTH/93	0	0.38	1.45	0.99	40	56
HCTH/407	0	0.34	1.51	1.00	36	56
τ-HCTH	0	0.32	1.53	1.00	36	82
TDHF	WFT	1.19	0.88	1.01	36	119
LC-M06-L	0–100	0.57	1.35	1.01	40	27, 73
TPSS	0	0.26	1.63	1.03	36	61
CIS	WFT	1.29	0.91	1.07	36	120
BP86	0	0.38	1.62	1.08	36	49, 50
BP86(VWN5)	0	0.38	1.62	1.08	36	45, 49, 50
PBE	0	0.40	1.70	1.13	36	43
BLYP	0	0.40	1.88	1.23	36	49, 51
MN12-L	0	0.49	1.80	1.23	40	19
M11-L	0	0.35	1.93	1.24	40	63
MN12-SX	25–0	0.38	1.90	1.24	40	33
N12	0	0.44	1.88	1.25	40	18
OLYP	0	0.36	1.97	1.27	36	51, 57
SOGGA11	0	0.62	2.40	1.62	40	48

^*a*^WFT indicates wave function theory; other rows are density functional theory, and *X* is the percentage of Hartree–Fock exchange. When a range of *X* is indicated, the first value corresponds to small interelectronic separations, and the second to large interelectronic separations.

We emphasize that the accuracy for electronic excitations is not an isolated issue but an integral part of the overall performance of a functional. Consistently good performance in both the ground state and excited states is crucial for the application of density functional methods to dynamical problems such as photochemistry. We conclude that MN15 is well suited for photochemical studies.

We also emphasize that in our training set, we only have excitation energies for atoms and for the first excitation energy of the Fe_2_ molecule. No TDDFT calculations were used in training (the few excitation energies in the training set were calculated as the difference in SCF energies, which is possible when the excited state has a different symmetry than the ground state).

### 6.4. Performance for transition-metal barrier height database

**Fig. 3:**
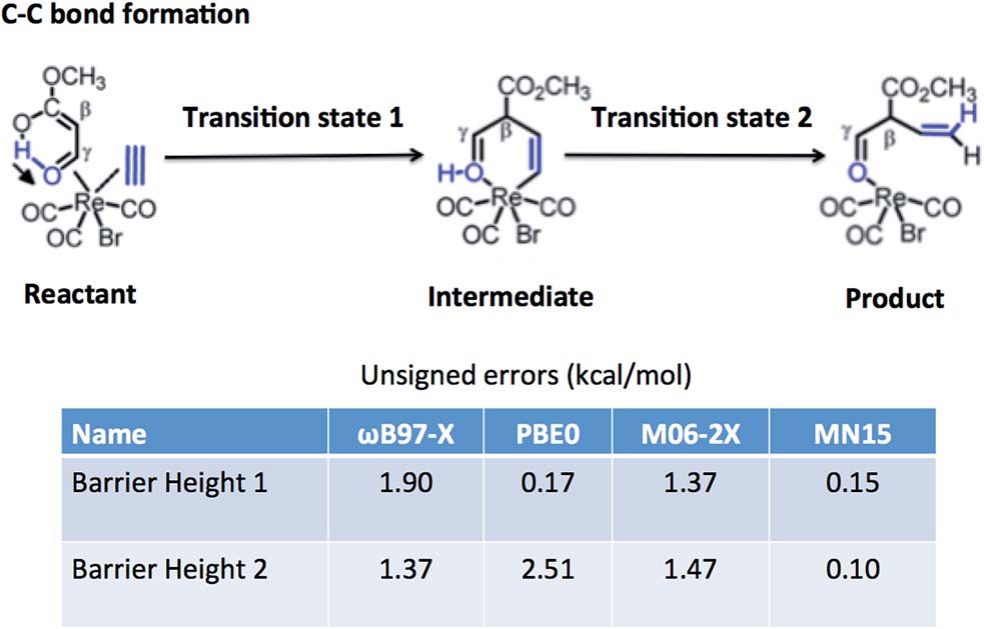
Reaction barrier heights of rhenium catalyzed reaction.

Table 15 tests the performance of the MN15 functional for the transition-metal reaction barrier heights published by Chen *et al.*^89^–^91^ The transition metals involved are Mo, W, Zr, and Re. This is a good test for MN15 because the training set involved no W or Re data, only one Zr datum (the Zr_2_ bond energy in SR-TM-BE17), and only three data for Mo (all for monatomic species: Mo and Mo^+^ in IP23 and the quartet-to-sextet transition energy of Mo in 4dAEE5). The results for MN15, MN15-L, and GAM were calculated by the present authors, and the other results in the table are the calculations of Chen *et al.* The functionals they selected for testing include two doubly hybrid functionals (both of which were also included in Section 6.2), B2GP-PLYP, which gives the best results, and B2-PLYP, which gives the fourth best results. Among the 20 functionals without nonlocal correlation in Table 15, the MN15 functional gives the second best results, behind M06. In Fig. 3, we show one typical example from this test set, which is a carbon–carbon formation process catalyzed by rhenium. The MN15 functional is the only one that gives good results for both barrier height 1 and barrier height 2. We conclude that the MN15 functional provides good results for transition-metal reaction barrier heights, even though there were no transition-metal barrier heights in the training set.

**Table 15:** The mean unsigned errors (MUE in kcal mol^–1^) of transition-metal reaction barrier heights^*a*^

Reactions^*a*^	MUE(Mo)	MUE(W)	MUE(Zr)	MUE(Re)	AMUE^*b*^
B2GP-PLYP	0.80	0.99	2.62	0.80	1.20
M06	1.12	1.19	0.75	1.87	1.25
**MN15**	**2.30**	**2.82**	**1.51**	**0.85**	**1.95**
B2-PLYP	1.43	1.32	3.77	2.03	1.99
TPSSh	2.12	1.77	3.36	1.08	2.01
**MN15-L**	**1.47**	**2.60**	**1.19**	**2.67**	**2.03**
PBE0	2.68	2.66	2.66	1.07	2.29
M06-2X	3.56	2.68	0.23	2.75	2.48
M06-L	2.57	2.32	1.22	4.12	2.61
TPSS	3.70	2.60	3.12	1.56	2.77
B3LYP	1.69	2.31	7.58	1.42	2.92
CAM-B3LYP	3.46	2.28	5.78	1.78	3.16
RPBE	2.55	2.37	6.42	2.44	3.21
B1LYP	2.37	2.77	7.09	1.66	3.21
GAM	2.71	2.43	4.37	4.15	3.29
PBE	3.79	3.74	2.63	2.98	3.36
ωB97X	5.40	3.05	3.22	1.53	3.39
BP86	4.35	2.90	4.21	2.62	3.50
BLYP	3.02	3.29	7.09	2.14	3.66
BMK	4.13	3.98	5.16	1.72	3.71
OLYP	3.76	3.12	13.29	2.51	5.09
LC-ωPBE	6.24	4.34	NA	1.72	NA

^*a*^There are respectively 6, 6, 4, and 5 reactions in the database for Mo, W, Zr, and Re reaction barrier heights.

^*b*^The average mean unsigned error for all 21 barrier heights.

### 6.5. Performance for transition-metal coordination database

Table 16 shows the performance of 11 density functionals for the transition-metal coordination database (WCCR10).^92^ The results for MN15, MN15-L, and GAM were calculated by the present authors, and the other results in the table are calculations from ref. 92. The molecules used for training are relatively small compared to all the molecules in the WCCR10 database. We find that the MN15 functional gives the best results for the WCCR10 database with an MUE of 5.04 kcal mol^–1^. The MN15 functional gives the fourth best results for TMBE33 in Tables 6–8, with an MUE of 5.54 kcal mol^–1^, which is close to the MUE that MN15 gives for the WCCR10 database. Therefore, the good performance of the MN15 functional for transition-metal bond energies in TMBE33 does transfer well to other databases.

**Table 16:** Mean unsigned errors (kcal mol^–1^) for the WCCR10 database

Functional^*a*^	Type	WCCR10
**MN15**	Hybrid meta-NGA	**5.04**
**MN15-L**	meta-NGA	**5.46**
PBE0	Hybrid GGA	6.40
GAM	NGA	6.60
PBE	GGA	7.58
TPSSh	Hybrid meta-GGA	7.62
TPSS	GGA	7.84
B97-D-D2	GGA + MM^*b*^	8.59
B3LYP	Hybrid GGA	9.30
BP86	GGA	9.42
BP86-D3	GGA + MM^*b*^	10.62

^*a*^The MN15, MN15-L, and GAM results are from the present calculations, but all other results in this table are from ref. 92.

^*b*^MM denotes molecular (also called empirical dispersion correction), which in this case corresponds to atom–atom pairwise damped dispersion terms added post-SCF to the calculated energy.

### 6.6. Performance for semiconductor band gap database

Table 17 shows the performance of 28 density functionals for the semiconductor band gap database (SBG31).^93^ The functionals included in the table are the same as those in Table S17† of our previous paper^29^ plus MN15. The band gaps in this table are calculated, as is widely done,^122^ as the orbital energy of the lowest unoccupied crystal orbital minus the orbital energy of the highest occupied crystal orbital. When calculated this way, the MN15 functional gives the eleventh best results for the SBG31 database, with an MUE of 0.92 eV.

**Table 17:** Mean unsigned errors for the SBG31 database in eV

Functional	Type	SBG31
HSE06	RS-hybrid GGA	0.26
M11-L	meta-GGA	0.54
MGGA_MS2	meta-GGA	0.66
M06-L	meta-GGA	0.73
**MN15-L**	**meta-NGA**	**0.80**
MN12-L	meta-NGA	0.84
TPSS	meta-GGA	0.85
SOGGA11	GGA	0.89
HCTH407	GGA	0.89
OLYP	GGA	0.90
**MN15**	**Hybrid meta-NGA**	**0.92**
OreLYP	GGA	0.92
τ-HCTH	meta-GGA	0.92
VSXC	meta-GGA	0.97
PBE	GGA	0.98
N12	NGA	0.99
GAM	NGA	0.99
revTPSS	meta-GGA	1.00
RPBE	GGA	1.07
revPBE	GGA	1.08
BPW91	GGA	1.10
mPWPW	GGA	1.11
PW91	GGA	1.11
BP86	GGA	1.12
PBEsol	GGA	1.14
SOGGA	GGA	1.14
BLYP	GGA	1.14
GKSVWN5	LSDA	1.14

This kind of a test of an XCF suffers from the fact that the orbital energies are not physically observable quantities such as the quasiparticle energies appropriate for interpreting optical excitation, photoemission, and inverse photoemission experiments. A better way to calculate the observable band gap for a single-reference system is to use the Green's function–screened potential (GW) method to calculate true quasiparticle energies from the Kohn–Sham single-particle orbitals and orbital energies.^123^ It would be interesting to carry out such a calculation with the MN15 potential, but it is beyond the scope of the present paper.

### 6.7. Performance for transition-metal bond length database

Table 18 shows the performance of 14 density functionals for the homonuclear transition-metal dimer bond length database (TMBDL7).^94^ The functionals included in the table are the same as those in Table S17† of our previous paper^29^ plus MN15. The MN15 functional gives the seventh best results for the TMBDL7 database with an MUE of 0.045 Å. Interestingly, local functionals often give more accurate bond lengths and vibrational frequencies than nonlocal ones, and this trend is seen for the bond lengths in Table 18. Of the six nonlocal functionals in Table 15 (HSE06, MN15, B3PW91, B3LYP, B97-1, and ωB97X-D), MN15 gives the second best MUE, and its MUE of 0.045 Å may be compared to an average MUE for the five other nonlocal functionals of 0.063 Å.

**Table 18:** Homonuclear transition-metal dimers: equilibrium bond lengths (Å) and mean unsigned errors as compared to experiment

	Cu_2_	Au_2_	Ni_2_	Pd_2_	Pt_2_	Ir_2_	Os_2_	MUE(1)^*b*^	MUE(2)^*b*^
N12	2.224	2.543	2.110	2.501	2.366	2.262	2.282	0.026	0.028
MGGA_MS2	2.210	2.527	2.080	2.493	2.359	2.254	2.275	0.028	0.028
**MN15-L**	**2.274**	**2.540**	**2.138**	**2.520**	**2.346**	**2.232**	**2.257**	**0.028**	**0.030**
M06-L	2.214	2.555	2.101	2.500	2.380	2.274	2.294	0.033	0.034
LSDA	2.215	2.495	2.118	2.373	2.353	2.271	2.354	0.038	0.043
HSE06	2.258	2.258	2.551	2.078	2.514	2.358	2.250	0.041	0.043
**MN15**	**2.277**	**2.501**	**2.045**	**2.468**	**2.301**	**2.182**	**2.204**	**0.041**	**0.045**
PBE	2.278	2.552	2.135	2.397	2.391	2.302	2.384	0.062	0.047
mPWPW	2.293	2.549	2.088	2.359	2.369	2.282	2.369	0.068	0.050
GAM	2.306	2.609	2.189	2.536	2.408	2.283	2.292	0.059	0.058
B3PW91	2.288	2.552	2.095	2.367	2.375	2.287	2.373	0.068	0.056
B3LYP	2.292	2.577	2.099	2.411	2.392	2.301	2.387	0.071	0.059
B97-1	2.278	2.566	2.391	2.617	2.368	2.259	2.279	0.082	0.073
ωB97X-D	2.214	2.555	2.101	2.500	2.380	2.274	2.294	0.083	0.085
Exp.^*a*^	2.219	2.472	2.155	2.480	2.333	2.270	2.280	0.000	0.000

^*a*^The experimental values are from ref. 94.

^*b*^The bond lengths in the table and MUE(1) are calculated with the LANL2DZ basis set for comparison with previous work, and MUE(2) is averaged over this basis set and also over the higher-quality def2-TZVP basis set.

### 6.8. Performance for organic molecule geometry database

Table 19 shows the performance of 13 density functionals for a recently published structural database called SE47, which denotes semi-experimental structures of 47 organic molecules.^95^ The functionals included in this table are those selected as the best ranking functionals in Table 10. The SE47 database consists of 193 bond lengths of the 47 organic molecules. In this case MN15 gives the best bond lengths with an MUE of only 0.0033 Å. In Table 19 the nonlocal functionals (hybrid functionals) do better, on average, than the local ones. Since we only have ten molecular structure data (MS10) in our training set, the good performance of the MN15 functional against these 193 bond length shows that our functional is highly transferable to quantities not in our training set.

**Table 19:** Mean unsigned errors for the SE47 database in Å

Functionals	Types	MUE of SE47^*a*^
**MN15**	**Hybrid meta-NGA**	**0.0033**
B3LYP	Hybrid GGA	0.0037
M06-2X	Hybrid meta-GGA	0.0040
ωB97X-D	RS-hybrid GGA + MM^*b*^	0.0043
M06-L	meta-GGA	0.0044
GAM	NGA	0.0044
HSE06	RS-hybrid GGA	0.0046
τ-HCTHhyb	Hybrid meta-GGA	0.0046
M06	Hybrid meta-GGA	0.0063
TPSS	meta-GGA	0.0078
MN15-L	meta-NGA	0.0098
PBE	GGA	0.0103
BP86	GGA	0.0112

^*a*^The SE47 is a new geometry database published by M. Piccardo *et al.*^95^ There are 193 bond length from 47 organic molecules being calculated by 13 functionals above. The original database SE47 includes both bond lengths and bond angles, however, in the present paper we only compare the bond lengths.

^*b*^MM denotes molecular mechanics (also called empirical dispersion correction), which in this case corresponds to atom–atom pairwise damped dispersion terms added post-SCF to the calculated energy.

## 7. Concluding remarks

The new functional presented in this article is the culmination of a large body of work. Our group tested the performance of a variety of functionals on various kinds of properties one at a time and used the knowledge so gained to improve our databases. Some of our small databases were built by selecting representative data from larger databases by statistical processes. Our strategy for developing a better density functional has two components; one is the development of a good database for training, and the other is the choice of functional form. In the current round of functional optimization, we first worked out the best gradient approximation, which is the GAM functional.^26^ Next we made the MN15-L functional^29^ by adding kinetic energy density to the ingredients in the GAM functional. As we can see from Table 7, averaged over all 471 energetic data in Database 2015B, MN15-L is the best functional among all the local approximations, and it even does better than all of the hybrid functionals in Tables 7 and 8 except for the MN15 functional presented here. The MN15 functional adds a portion of Hartree–Fock exchange to the ingredients of the MN15-L functional, and improves the mean unsigned error on the 471 data from 2.36 kcal mol^–1^ to 2.08 kcal ml^–1^. The MN15 functional gives best results for single-reference systems (SR313) and second best results for multi-reference systems (MR54). It has outstanding performance for noncovalent interactions. It also has the best performance for the ten bond distances in the MS10 database. Perhaps most significantly of all, it improves the average ranking over 28 diverse energetic databases from 17 to 9, which means it is a more universal functional than any previous functional.

In order to further test the broad applicability of the new functional, we tested it on the following additional databases: S492, EE69, TMBH21, WCCR10, SBG31, TMBDL7, and SE47. This gives a total of 823 data that are not in our training set in addition to 481 pieces of data in Database 2015B that was used for training and broader testing. MN15 has good performance on the data not included in the training set as well as on the data used for training. It is especially noteworthy that it has good accuracy for both valence and Rydberg electronic excitations, which is not achieved by most other functionals; such simultaneous accuracy is important for applications such as photochemistry.

Many applications of density functional theory, such as understanding and designing new energy materials and catalysts, require more than one property to be accurately modeled. The broad accuracy of MN15 can be very useful for studying these applications.

## Competing financial interests

The authors declare no competing financial interests.

## Supplementary Material

Supplementary information
