# Correction: MN15: A Kohn–Sham global-hybrid exchange–correlation density functional with broad accuracy for multi-reference and single-reference systems and noncovalent interactions

**DOI:** 10.1039/c6sc90044e

**Published:** 2016-07-12

**Authors:** Haoyu S. Yu, Xiao He, Shaohong L. Li, Donald G. Truhlar

**Affiliations:** a Department of Chemistry , Chemical Theory Center , Inorganometallic Catalyst Design Center , Minnesota Supercomputing Institute , University of Minnesota , Minneapolis , Minnesota 55455-0431 , USA . Email: truhlar@umn.edu; b State Key Laboratory of Precision Spectroscopy , Department of Physics , East China Normal University , Shanghai , 200062 , China; c NYU-ECNU Center for Computational Chemistry at NYU Shanghai , Shanghai , 200062 , China

## Abstract

Correction for ‘MN15: A Kohn–Sham global-hybrid exchange–correlation density functional with broad accuracy for multi-reference and single-reference systems and noncovalent interactions’ by Haoyu S. Yu *et al.*, *Chem. Sci.*, 2016, DOI: ; 10.1039/c6sc00705h.



## 

There were errors in some of the rankings in Table 11 and a corrected table is provided here. This correction does not affect the conclusions of the paper.

**Table 11:** The rankings (out of 83 functionals) of 12 selected functionals for 28 atomic and molecular databases

Name	BP86	PBE	B3LYP	TPSS	HSE06	M06-L	τ-HCTHhyb	ωB97X-D	M06-2X	M06	MN15-L	MN15
SR-MGM-BE9	27	19	53	13	39	32	9	14	3	35	20	18
SR-MGN-BE107	75	69	48	46	33	29	20	11	2	8	12	1
SR-TM-BE17	52	49	20	22	11	37	38	3	58	18	2	6
MR-MGM-BE4	48	46	23	13	33	7	4	49	57	3	1	2
MR-MGN-BE17	73	74	30	13	34	3	15	44	37	10	1	2
MR-TM-BE13	61	62	16	43	23	18	2	7	69	5	8	9
IsoL6/11	51	44	57	74	10	61	32	5	20	11	12	31
IP23	75	63	55	38	32	29	27	7	15	52	2	4
EA13/03	72	27	30	31	45	67	6	9	19	8	22	1
PA8	21	17	2	66	8	44	47	61	34	40	55	9
πTC13	33	27	38	67	44	49	61	45	1	16	20	8
HTBH38/08	76	77	39	70	40	37	47	25	5	21	8	3
NHTBH38/08	72	70	43	75	37	39	42	38	3	21	15	13
NCCE30	65	57	46	56	33	19	39	8	2	10	22	3
AE17	57	73	60	59	70	22	17	16	1	5	21	23
ABDE13	51	38	57	69	36	41	34	10	9	19	33	14
HC7/11	48	11	67	49	37	7	35	19	1	6	12	9
3dAEE8	47	32	20	44	54	27	66	18	17	41	1	12
4dAEE5	23	12	35	27	24	51	63	58	70	62	1	30
pEE5	19	30	11	6	56	66	28	69	40	50	42	35
DC9/12	62	60	46	55	33	40	36	21	7	2	8	16
2pIsoE4	47	36	73	58	31	43	48	18	16	13	20	1
4pIsoE4	50	28	75	37	38	51	49	29	40	23	68	12
S6x6	69	38	60	55	28	8	45	1	5	13	20	2
NGDWI21	79	18	66	44	17	27	46	40	21	51	3	2
MR-TMD-BE3	16	29	50	12	59	1	19	56	75	46	25	31
SMAE3	67	65	62	45	49	21	15	24	35	2	6	1
MS10	54	46	50	29	9	2	18	33	66	35	7	1
Lowest	79	77	75	75	70	67	66	69	75	62	68	35
Average	53	43	44	43	34	31	32	26	26	22	17	11

The final sentences of Section 6.1 are corrected as follows: “The MN15 functional gives the best average ranking, which is 11, with MN15-L being second with an average ranking of 17. The average ranking of the other functionals in the table is in the range of 22–53. Furthermore, every other functional in the table has at least one ranking of 62 or lower, whereas MN15 ranks lower than 35th in none of the 28 categories and lower than 25th in only four. MN15-L also ranks lower than 25th in only four categories; M06, M06-2X, and ωB97X-D rank lower than 25th in 9–11 categories each; M06-L and τ-HCTHhyb rank lower than 25th in 18 categories; and the other functionals in the table rank lower than 25th in 21–24 categories”.

In the last sentence of the first paragraph of Section 7, “9” should be “11”.

The Royal Society of Chemistry apologises for these errors and any consequent inconvenience to authors and readers.

