# Supplementary material for: MN15: A Kohn–Sham global-hybrid exchange–correlation density functional with broad accuracy for multi-reference and single-reference systems and noncovalent interactions
Source: Chem Sci. 2016 Apr 6;7(8):5032–51. doi: 10.1039/c6sc00705h (PMC6018516; doi:10.1039/c6sc00705h)
Supplement: Supplementary file 1 [file SC-007-C6SC00705H-s001.pdf]

Electronic Supplementary Material (ESI) for Chemical Science.
This journal is © The Royal Society of Chemistry 2016

ELECTRONIC SUPPORTING INFORMATION

for a paper in *Chemical Science* entitled

MN15: A New Kohn-Sham Density Functional with Broad Accuracy for Multi-reference and Single-reference Systems and Noncovalent Interactions

Haoyu S. Yu,[a] Xiao He,[a,b,c] Shaohong L. Li,[a] and Donald G. Truhlar*[a]

[a]*Department of Chemistry, Chemical Theory Center, Inorganometallic Catalyst Design Center, and Supercomputing Institute, University of Minnesota, Minneapolis, Minnesota 55455-0431, USA. *E-mail: truhlar@umn.edu*

[b]*State Key Laboratory of Precision Spectroscopy and Department of Physics, East China Normal University, Shanghai, 200062, China*

[c]*NYU-ECNU Center for Computational Chemistry at NYU Shanghai, Shanghai, 200062, China*

March 31, 2016

Table of contents

Density functionals tested in this paper and SI. S-3

Grids, basis sets, and geometries S-4

Table S1. 83 density functionals tested in the paper and SI with the percentage of nonlocal Hartree-Fock exchange, the year that the functional was published, and the original reference or references S-5

Table S2. The mean unsigned errors on 27 databases of atomic and molecular energies as calculated by local spin density approximations (LSDAs) and generalized gradient approximations (GGAs) S-8

Table S3. The mean unsigned errors on 27 databases of atomic and molecular energies as calculated by GGAs, N12, GAM, and global hybrid GGAs S-9

Table S4. The mean unsigned errors on 27 databases of atomic and molecular energies as calculated by range-separated hybrid GGAs and global hybrid GGAs S-10

Table S5. The mean unsigned errors on 27 databases of atomic and molecular energies as calculated by range-separated hybrid GGAs, N12-SX, meta-GGAs, and MN15-L S-11

Table S6. The mean unsigned errors on 27 databases of atomic and molecular energies as calculated by hybrid meta-GGAs S-12

Table S7. The mean unsigned errors on 27 databases of atomic and molecular energies as calculated by hybrid meta-GGAs, M11, MN12-SX, and MN15 S-13

Table S8. The mean unsigned errors intermolecular charge transfer database (CT7) calculated by 83 density functionals in the order of increasing MUE of CT7 database S-14

Table S9. The mean unsigned errors for molecular energy database and its subdatabases calculated by 83 density functionals in the same order as Table S1 S-15

Table S10. The average and median mean unsigned errors for the molecular energy database and its subdatabases calculated by 83 density functionals S-17

Table S11. The mean unsigned errors for molecular energy databases and its subdatabases calculated by 83 density functionals in the order of increasing MUE of atomic and molecular database (AME471) S-18

Table S12. The mean unsigned errors on three molecular databases calculated by 83 density functionals in the same order as Table S1 S-20

Table S13. The mean unsigned errors on three molecular databases calculated by 83 density functionals in the order of increasing MUE of molecular structure database (MS10) S-21

Table S14. Geometries, charge, and multiplicities of alkyl bond dissociation energies (ABDE13), six dimers at six intermonomer distances (S6x6), semiconductor band gaps (SBG31), and excitation energies of selected organic molecules (EE69) S-22

References S-65

Density functionals tested in the paper and supporting information

In order to evaluate the performance of the MN15 functional, we tested a large number of previously developed density functionals for comparison; In particular, we tested 82 previously developed density functionals of 12 types plus the present MN15 functional, which is a 13th type. The full set of functionals tested, sorted by type and by year within a type, is as follows:

- LSDA: GKSVWN5,[1,2,3] GKSVWN3, [1,2,3]
- GGA: SOGGA,[4] PBEsol,[5] SOGGA11,[6] B86P86,[7,8] B86LYP,[7,10] BP86,[8,9] BLYP,[9,10] BR89LYP,[10,11] B86PW91,[7,12] PW91,[12] BPW91,[9,12] PBE,[13] mPWPW,[14] revPBE,[15] RPBE,[16] HCTH407,[17] OLYP,[10,18] MPWLYP1W,[19] PBE1W,[19] PBELYP1W,[19] MOHLYP,[20] B97-D,[21] MOHLYP2,[22] and OreLYP[10,18,23]
- NGA: N12[24] and GAM[25]
- meta-GGA: VSXC,[26] τ-HCTC,[27] TPSS,[28] TPSSLYP1W,[19] M06-L,[29] revTPSS,[30] M11-L,[31] MGGA_MS2[32]
- meta-NGA: MN12-L[33] and MN15-L[34]
- global hybrid GGA: HFLYP,[10,35] HFPW91,[12,35] B3PW91,[9,36] B3LYP,[9,10,37] PBE0,[38] mPW1PW,[39] B1LYP,[40] B98,[41] B97-1,[42] MPW1K,[43] O3LYP,[44] B97-2,[45] MPW3LYP,[46] MPWLYP1M,[20] B97-3,[47] and SOGGA11-X[48]
- range-separated hybrid-GGA: CAM-B3LYP,[49] LC-ωPBE,[50,51,52,53], HSE06,[54,55] ωB97,[56] ωB97X[56]
- range-separated hybrid-GGA plus molecular mechanics (also called empirical dispersion correction): ωB97X-D[57]
- range-separated-hybrid-NGA: N12-SX[58]
- global hybrid meta-GGA: TPSSh,[59] τ-HCTHhyb,[27] BB1K,[9,60,61] MPWB1K,[46] MPW1B95,[46] BMK,[62] TPSS1KCIS,[63] MPWKCIS1K,[22] MPW1KCIS,[22] PBE1KCIS,[64] PWB6K,[65] PW6B95,[65] M05,[66] M05-2X,[67] M06-HF,[68] M06,[69] M06-2X,[69] M08-HX,[70] M08-SO[70]
- range-separated-hybrid meta-GGA: M11 [71]
- range-separated-hybrid meta-NGA: MN12-SX[58]
- global-hybrid meta-NGA: MN15

For each of the functionals in the above list, Table S1 shows the percentage of nonlocal Hartree-Fock exchange, the year that the functional was published, and the original reference or references.

Grids, basis sets, and geometries

An ultrafine grid (99 radial shells with 590 angular points per shell) is used for integration of density functionals.

The *Gaussian 09* keyword stable=opt is used for finding the lowest-energy solution to the Kohn-Sham equations by breaking the symmetry of the Slater determinant when necessary.

The basis sets and geometries used for molecules that are also in a previous database called Database 2015A are the same as specified previously.[34] The def2-QZVP basis set[72] is used for the 36 weak interactions and S66x8 database. The ma-TZVP basis set[73] is used for the 13 alkyl bond dissociation energies. The 6-311(2+, 2+)G** basis set as proposed by Wiberg *et al.*[74] is used for calculations of excitation energies of selected organic molecules. For the transition metal barrier height test sets, the cc-pVQZ[75] basis set is used for H, B, C, N, and O; the cc-pV(Q+d)Z[76] basis set is used for P, S, and Cl; the cc-pVQZ-PP[77,78] basis set is for Mo, W, and Zr; the cc-pVTZ,[75] cc-pV(T+d)Z,[76] and cc-pVTZ-PP[78] basis sets are used for reactions that involves Re atoms. The aug-cc-pVTZ[75,79] basis set is used for testing the geometries of 47 organic molecules (SE47).

The geometries that are used for transition metal barrier heights database (TMBH21) are reported in the supporting information of the original papers.[80,81,82] The reference geometries for the SE47 test set are reported in reference 83. The geometries used for calculating semiconductor band gaps (database SBG31), alkyl bond dissociation energies (ABDE13), six dimers at six intermonomer distances (S6x6), and excitation energies of selected organic molecules (EE69) are reported in this supporting information.

Table S1. Exchange–correlation functionals tested in this study

Category	Type	*Abbrev.*	X^a	Year	Method	Ref.
local	LSDA	LSDA	0	1980	GKSVWN5[b]	1,2,3
			0	1980	GKSVWN3[b]	1,2,3
	GGA - exchange correct to second order	GGA	0	2008	SOGGA	4
			0	2008	PBEsol	5
			0	2011	SOGGA11	6
	GGA - other	GGA	0	1986	B86P86	7,8
			0	1987	B86LYP	7,10
			0	1988	BP86	8,9
			0	1988	BLYP	9,10
			0	1989	BR89LYP	10,11
			0	1991	B86PW91	7,12
			0	1991	PW91[c]	12
			0	1991	BPW91	9,12
			0	1996	PBE	13
			0	1997	mPWPW	14
			0	1997	revPBE	15
			0	1999	RPBE	16
			0	2001	HCTH407	17
			0	2001	OLYP	10,18
			0	2005	MPWLYP1	19
			0	2005	PBE1W	19
			0	2005	PBELYP1W	19
			0	2005	MOHLYP	20
			0	2006	B97-D	21
			0	2009	MOHLYP2	22
			0	2009	OreLYP	10,18,23
	NGA	NGA	0	2012	N12	24
			0	2015	GAM	25
	meta-GGA	mGGA	0	1998	VSXC	26
			0	2002	τ-HCTH	27
			0	2003	TPSS	28
			0	2005	TPSSLYP1	19
			0	2006	M06-L	29
			0	2009	revTPSS	30
			0	2011	M11-L	31
			0	2013	MGGA_MS2	32
	meta-NGA	mNGA	0	2012	MN12-L	33
			0	2015	MN15-L	34
nonlocal	global-hybrid GGA	GGAh	100	1987	HFLYP	10,35
			100	1991	HFPW91	12,35
			20	1992	B3PW91	9,36
			20	1994	B3LYP	9,10,37
			25	1996	PBE0	38
			25	1997	mPW1PW	39

		25	1997	B1LYP	40
		21.98	1998	B98	41
		21	1998	B97-1	42
		42.80	2000	MPW1K	43
		11.61	2001	O3LYP	44
		21	2001	B97-2	45
		21.8	2004	MPW3LYP	46
		5	2005	MPWLYP1	20
		26.93	2005	B97-3	47
		35.42	2011	SOGGA11-X	48
RS-hybrid GGA[d]	GGArsh	19-65	2004	CAM-	49
		0-100	2006	LC-ωPBE	50-53
		25-0	2006	HSE06	54,55
		0-100	2008	ωB97	56
		15.77-100	2008	ωB97X	56
RS-hybrid NGA	NGArsh	25-0	2012	N12-SX	58
RS-hybrid GGA+MM[e]	NGArsh-D	22.2-100	2008	ωB97X-D	57
global-hybrid meta-GGA	mGGAh	10	2002	TPSSh	59
		15	2002	τ-HCTHhyb	27
		42	2004	BB1K	9,60,61
		44	2004	MPWB1K	46
		31	2004	MPW1B95	46
		42	2004	BMK	62
		13	2005	TPSS1KCIS	63
		41	2005	MPWKCIS1	22
		15	2005	MPW1KCIS	22
		22	2005	PBE1KCIS	64
		46	2005	PWB6K	65
		28	2005	PW6B95	65
		28	2005	M05	66
		56	2005	M05-2X	67
		100	2006	M06-HF	68
		27	2008	M06	69
		54	2008	M06-2X	69
		52.23	2008	M08-HX	70
		56.79	2008	M08-SO	70
RS-hybrid meta-GGA[d]	mGGArsh	42.8-100	2011	M11	71
RS-hybrid meta-NGA	mNGArsh	25-0	2012	MN12-SX	58
global-hybrid meta-NGA	mNGAh	44	2015	MN15	present

[a] X is the percentage of nonlocal Hartree–Fock exchange. When a range is given, the first value is for small interelectronic distances, and the second value is for large interelectronic distances. Details of the functional form that joins these regions of interelectronic separation are given in the references.

[b] GVWN5 denotes the Gáspár approximation for exchange and the VWN5 fit to the correlation energy; GVWN3 denotes Gáspár approximation for exchange and the VWN fit to the correlation energy; this is an example of the local spin density approximation (LSDA), and it has the keyword SVWN5 and SVWN in the *Gaussian 09* program. Note that Kohn-Sham exchange is the same as Gáspár exchange, but Slater exchange (not tested here) is greater by a factor of 1.5.

[c] PW91 formally satisfies the gradient expansion for exchange to second order but only at such small values of the gradient that for practical purposes it should be grouped with functionals that do not satisfy the gradient expansion to second order.

[d] RS denotes range-separated.

[e] MM denotes molecular mechanics (also called empirical dispersion correction), which in this case corresponds to atom-atom pairwise damped dispersion terms added post-SCF to the calculated energy.

Table S2. MUEs (kcal/mol) for the AME471 database and subdatabases: LSDA, GGA

Type	——LSDA——		GGA														
Functional	SVWN5	SVWN3	B86P86	B86LYP	BP86	BLYP	BR89LYP	B86PW91	BPW91	PW91	PBE	HCTH407	mPWPW	revPBE	RPBE	OLYP	MPWLYP1W
SR-MGM-BE9	12.54	14.77	3.58	4.46	3.28	5.50	4.95	2.65	3.52	2.62	2.72	3.87	3.07	4.72	5.06	5.14	5.05
SR-MGN-BE107	16.21	17.88	3.94	2.87	4.06	2.78	2.72	2.51	2.49	3.51	3.40	2.55	2.80	2.99	3.35	2.32	2.60
SR-TM-BE17	20.34	22.71	6.46	6.40	6.84	6.20	5.90	5.60	6.79	8.22	7.20	8.05	6.18	5.67	5.76	9.01	8.03
MR-MGM-BE4	24.56	27.63	9.94	7.64	9.49	8.75	8.65	7.55	8.03	10.26	9.31	10.11	9.02	6.24	6.43	8.39	10.57
MR-MGN-BE17	36.89	40.23	6.59	8.20	13.87	6.67	5.79	12.15	10.74	14.80	14.80	5.24	12.45	5.94	5.51	5.15	10.88
MR-TM-BE13	26.60	29.02	22.93	20.62	9.68	9.80	6.48	7.85	8.82	10.18	12.73	19.53	9.22	7.39	6.80	5.58	11.93
IsoL6/11	2.05	2.19	1.35	2.63	2.28	3.73	3.59	1.34	2.38	1.92	1.98	3.02	2.16	2.82	2.99	3.44	3.67
IP23	9.59	19.89	8.97	5.84	8.44	6.52	8.68	6.69	6.30	7.29	6.19	6.81	6.85	5.00	4.92	3.12	7.16
EA13/03	5.70	16.41	2.57	4.18	4.21	2.68	5.50	3.98	2.26	2.60	2.27	3.70	2.31	2.40	2.37	3.60	3.20
PA8	5.07	4.55	3.59	2.68	1.41	1.58	4.28	4.89	1.88	1.30	1.34	2.84	1.52	2.00	1.98	2.40	1.61
πTC13	4.80	4.66	10.36	10.24	5.85	6.07	4.39	11.65	7.08	5.73	5.59	8.23	6.41	7.15	7.20	8.26	5.96
HTBH38/08	17.56	17.79	7.74	6.21	9.16	7.52	5.56	5.93	7.38	9.60	9.31	5.48	8.43	6.58	6.43	5.63	8.17
NHTBH38/08	12.42	12.36	7.97	7.83	8.72	8.53	8.08	6.53	7.26	8.80	8.42	6.29	8.03	6.82	6.82	5.25	9.04
NCCE30	3.29	3.44	2.54	2.50	1.73	1.84	1.57	2.48	2.02	1.48	1.42	1.28	1.51	1.90	1.72	2.81	1.50
AE17	421.13	309.99	29.64	24.85	16.92	8.68	28.42	24.67	11.95	4.63	47.24	16.80	12.55	10.88	9.39	10.13	35.60
HC7/11	21.45	23.50	6.33	19.48	9.95	27.39	31.82	5.02	10.77	4.55	3.97	14.97	8.08	13.65	14.96	17.01	26.30
3dEE8	12.79	14.04	10.58	12.82	11.14	11.92	9.77	10.82	13.84	11.12	11.88	14.53	11.22	10.56	10.31	12.00	12.08
4dAEE5	14.10	5.28	9.57	6.30	5.07	5.73	7.69	4.70	5.03	4.73	4.70	7.75	4.89	4.49	4.27	5.94	5.67
pEE5	4.36	5.56	3.11	8.20	3.46	5.10	8.14	3.62	6.33	4.14	3.96	4.27	5.22	4.37	3.51	2.09	6.24
DC9/12	17.35	20.43	5.78	7.32	15.11	17.88	12.20	6.60	16.21	13.94	14.99	19.74	14.76	20.35	21.48	21.71	16.24
2pIsoE4	2.05	2.05	3.38	5.57	3.21	5.45	6.23	3.63	3.43	2.87	2.73	4.59	3.20	3.59	3.70	3.95	5.47
4pIsoE4	3.05	2.97	3.01	4.20	2.87	4.00	5.25	2.56	2.41	2.58	2.43	3.29	2.50	2.16	2.16	2.15	4.03
NGDWI21	0.21	0.23	0.08	0.18	0.53	0.38	0.16	0.14	0.59	0.16	0.10	0.25	0.22	0.28	0.18	0.32	0.18
MR-TMD-BE3	46.72	51.26	162.16	153.88	18.80	30.80	10.56	15.80	24.36	22.17	23.71	16.16	22.10	19.94	18.68	17.38	13.11
SMAE3	99.54	109.72	13.93	8.17	16.39	6.39	9.84	5.46	4.58	17.49	17.50	7.45	13.66	7.89	11.17	5.16	4.80
S6x6	3.12	3.38	2.66	3.58	4.13	4.96	3.33	3.92	5.39	2.28	2.66	4.61	3.81	5.60	5.30	7.90	3.78
ABDE13	13.96	16.10	7.26	11.96	7.35	11.98	11.28	9.21	9.32	4.96	5.08	10.17	7.37	11.43	12.02	11.25	10.82

Table S3. MUEs (kcal/mol) for the AME471 database and subdatabases: GGA, NGA, GGAh

Type	GGA									NGA		GGAh				
Functional	PBE1W	PBELYP	MOHLYP	B97-D	SOGGA	PBEsol	MOHLYP2	SOGGA11	OreLYP	N12	GAM	HFLYP	HFPW91	B3PW91	B3LYP	PBE0
SR-MGM-BE9	3.51	5.43	8.21	6.56	4.73	4.74	22.29	9.00	4.44	6.06	2.00	11.00	13.99	3.83	4.58	3.65
SR-MGN-BE107	3.05	2.99	3.96	2.13	7.27	7.28	19.76	2.77	2.56	2.38	2.27	9.68	14.71	2.03	2.45	1.98
SR-TM-BE17	7.79	7.54	8.78	17.85	11.04	10.78	16.80	10.89	7.15	7.76	6.31	16.15	15.43	5.03	5.48	4.35
MR-MGM-BE4	10.47	10.60	75.40	11.30	14.48	15.81	12.63	7.44	8.35	9.10	7.76	40.94	42.75	9.10	7.76	8.94
MR-MGN-BE17	12.86	10.97	4.89	3.78	21.29	23.16	28.58	8.57	4.25	6.93	4.22	48.43	189.76	4.82	5.09	5.15
MR-TM-BE13	8.81	7.60	7.80	7.29	16.78	16.28	19.07	16.57	4.92	9.39	4.94	30.93	28.85	4.96	5.33	4.47
IsoL6/11	2.42	3.95	4.31	1.73	1.89	1.55	6.80	1.73	3.39	1.73	1.96	3.21	4.30	1.52	2.61	1.38
IP23	7.89	9.13	3.84	3.09	4.84	5.82	10.15	5.92	3.03	4.36	4.53	9.47	24.71	4.81	5.51	3.39
EA13/03	3.67	5.39	3.67	2.46	2.70	2.16	13.20	5.23	2.32	4.12	4.49	9.52	10.47	2.13	2.33	2.79
PA8	1.44	1.65	1.86	3.15	2.33	2.10	5.28	2.11	1.70	1.35	3.84	3.32	8.41	1.89	1.02	1.19
πTC13	6.14	6.28	6.67	8.82	4.06	4.20	10.81	7.41	7.27	8.61	8.59	9.65	16.74	7.00	6.03	6.11
HTBH38/08	8.45	7.85	5.49	7.17	12.88	12.69	4.14	6.57	6.28	6.94	5.35	7.22	14.98	4.03	4.23	4.22
NHTBH38/08	8.20	8.79	5.80	6.34	9.68	9.86	3.41	4.32	5.57	6.86	5.15	7.04	9.53	3.62	4.55	3.43
NCCE30	1.38	1.39	3.36	0.67	1.98	1.95	6.21	1.30	3.00	1.62	1.29	0.75	40.42	1.43	1.19	0.85
AE17	9.26	63.05	256.11	10.33	283.06	245.90	14.80	10.06	2.37	14.21	10.18	8.53	10.25	4.83	18.29	38.57
HC7/11	12.25	28.09	25.50	13.46	17.88	13.31	47.73	6.26	16.34	4.27	6.24	16.49	25.72	4.35	16.80	9.40
3dEE8	11.16	12.35	10.51	15.87	11.26	11.31	9.79	13.78	12.37	20.80	10.45	13.37	17.79	9.42	9.46	8.90
4dAEE5	4.65	5.74	5.36	10.01	4.77	8.48	3.17	7.60	6.42	10.24	5.23	6.78	5.04	4.79	5.67	4.69
pEE5	3.46	7.29	4.18	9.40	6.30	5.15	14.21	5.01	3.25	14.86	2.99	4.15	11.87	5.57	2.87	5.74
DC9/12	15.73	17.41	29.07	16.45	14.61	13.34	54.06	16.65	22.57	10.20	23.07	17.08	22.27	10.95	12.02	8.81
2pIsoE4	3.69	5.63	4.33	4.07	1.44	1.71	6.53	1.72	3.72	3.41	5.02	3.45	1.95	2.88	4.69	2.17
4pIsoE4	2.83	4.14	2.73	3.60	2.29	2.28	4.99	3.27	2.22	1.73	3.57	5.46	3.21	2.67	4.24	2.32
NGDWI21	0.11	0.15	0.41	0.07	0.08	0.08	0.41	0.65	0.39	0.39	0.02	0.09	0.19	0.45	0.28	0.11
MR-TMD-BE3	19.11	12.59	28.02	21.64	29.78	27.86	46.53	20.50	9.48	18.30	11.53	204.96	261.62	52.71	26.41	67.94
SMAE3	9.70	4.07	17.20	11.00	50.60	49.03	100.51	18.63	5.29	7.76	5.54	79.36	71.69	9.24	13.24	8.38
S6x6	3.56	2.61	9.11	0.52	2.19	2.26	14.39	4.06	8.23	3.97	1.68	2.63	2.39	4.26	3.78	2.44
ABDE13	7.53	11.03	15.23	9.21	4.21	3.25	33.05	3.75	10.77	3.84	6.28	7.39	5.36	7.00	8.65	4.27

Table S4. MUEs (kcal/mol) for AME471 and subdatabases: GGAh, GGArsh

Type	GGAh											GGArsh		
Functional	mPW1PW	B1LYP	B98	B97-1	MPW1K	O3LYP	MPW3LYP	MPWLYP1M	B97-2	B97-3	SOGGA11-X	CAM-B3LYP	LC-ωPBE	HSE
SR-MGM-BE9	4.09	6.44	2.42	2.17	6.06	5.18	5.72	4.86	2.09	3.08	4.27	4.04	3.28	3.77
SR-MGN-BE107	2.44	4.17	1.67	1.51	3.99	2.25	2.03	2.35	1.55	1.70	1.67	1.92	2.31	2.08
SR-TM-BE17	4.57	6.64	3.34	4.17	8.12	11.01	6.02	5.79	4.19	3.91	6.24	6.27	8.02	4.41
MR-MGM-BE4	8.64	8.55	6.60	6.05	11.00	8.31	21.77	8.84	6.63	6.96	10.50	10.45	14.00	8.52
MR-MGN-BE17	6.58	8.97	4.91	3.22	16.81	4.92	4.99	6.76	3.57	5.07	7.12	6.80	8.20	5.30
MR-TM-BE13	5.31	7.30	2.60	2.87	11.30	4.63	3.56	5.82	2.65	5.08	8.31	4.11	3.71	4.56
IsoL6/11	1.44	2.70	1.93	1.82	1.75	2.82	2.34	3.30	1.83	2.07	1.85	2.07	1.90	1.25
IP23	4.11	3.45	3.33	2.61	3.64	3.64	4.97	6.07	2.99	3.31	4.11	4.91	6.40	4.06
EA13/03	2.68	3.69	1.90	2.08	3.71	2.97	2.20	2.62	2.94	2.13	1.55	2.06	2.15	2.77
PA8	1.77	1.06	1.53	1.57	2.41	2.36	0.97	1.47	3.15	2.54	1.85	1.41	1.83	1.10
πTC13	6.85	6.03	7.13	7.04	7.28	8.00	5.46	5.60	8.16	7.37	6.08	3.69	4.27	6.20
HTBH38/08	3.55	3.19	4.16	4.39	1.34	4.06	4.71	7.48	3.24	2.28	1.79	3.18	1.18	4.23
NHTBH38/08	5.71	3.63	3.31	3.38	1.72	3.64	4.86	8.18	2.19	1.38	1.16	2.61	2.36	3.73
NCCE30	0.95	1.22	0.87	0.85	0.79	2.29	0.98	1.36	1.25	1.19	0.81	0.80	0.97	0.91
AE17	10.78	9.88	4.91	5.47	9.93	5.57	5.19	12.09	10.56	6.80	4.98	10.93	25.34	32.82
HC7/11	6.70	17.64	8.46	6.25	12.98	12.52	14.00	22.96	6.18	7.58	7.27	6.21	17.66	7.34
3dEE8	9.68	11.60	10.35	10.49	10.98	9.20	12.29	11.16	10.66	7.71	6.68	8.63	10.60	11.48
4dAEE5	4.81	5.89	6.84	7.27	4.73	5.46	5.63	5.70	8.06	6.96	4.34	5.67	5.72	5.07
pEE5	6.03	2.99	2.38	2.25	7.32	1.55	3.65	5.89	2.99	3.03	3.49	2.84	7.79	5.70
DC9/12	9.43	13.72	9.19	9.79	7.27	17.46	9.83	14.69	11.86	11.08	7.46	5.33	6.08	9.08
2pIsoE4	2.42	4.80	3.23	2.90	2.32	3.63	4.49	5.18	3.18	3.10	2.00	3.16	1.14	2.44
4pIsoE4	2.52	4.52	2.80	2.51	2.76	2.56	4.27	4.10	2.97	2.76	3.01	3.86	1.44	2.64
NGDWI21	0.21	0.28	0.10	0.08	0.20	0.27	0.15	0.17	0.24	0.40	0.43	0.15	0.23	0.10
MR-TMD-BE3	64.47	73.68	45.89	21.79	112.93	32.96	58.38	10.93	22.00	64.14	96.53	64.89	75.23	64.36
SMAE3	13.69	27.14	7.93	4.18	29.09	9.01	11.95	4.83	5.89	7.47	11.35	10.31	8.48	10.53
S6x6	3.22	3.89	2.87	2.45	2.85	6.67	2.58	3.28	4.19	3.85	2.70	2.52	2.83	2.36
ABDE13	6.09	10.04	7.41	4.00	5.30	9.36	7.08	9.76	3.78	3.92	0.98	4.94	3.67	4.94

Table S5. MUEs (kcal/mol) for the AME471 database and subdatabases: GGArsh, NGArsh, mGGA, mNGA

Type	—GGArsh—		GGArsh-D	NGArsh	——————————mGGA——————————									mNGA
Functional	ωB97	ωB97X	ωB97X-D	N12-SX	VSXC	τ-HCTH	TPSS	TPSSLYP1W	M06-L	revTPSS	M11-L	MN12-L	MGGA_MS2	MN15-L
SR-MGM-BE9	3.39	3.09	2.60	1.63	2.20	3.22	2.55	4.54	3.40	3.29	7.50	3.07	6.26	2.77
SR-MGN-BE107	1.52	1.43	1.37	1.55	2.06	2.15	2.43	4.05	2.03	2.24	1.75	1.37	2.62	1.39
SR-TM-BE17	6.91	6.30	3.74	5.89	6.78	8.98	6.11	7.17	6.24	5.97	5.66	8.64	6.63	3.65
MR-MGM-BE4	8.31	11.86	9.49	8.27	7.74	10.73	6.69	8.53	6.15	5.99	13.56	19.21	8.48	1.88
MR-MGN-BE17	7.74	7.11	6.33	6.09	3.79	4.77	4.25	4.79	3.11	4.61	4.05	4.27	7.86	2.08
MR-TM-BE13	4.86	4.44	3.29	3.18	5.92	3.79	8.87	5.33	4.40	6.70	4.54	14.09	5.30	3.63
IsoL6/11	1.48	1.55	1.15	1.78	4.69	2.87	3.66	5.73	2.76	3.96	1.57	1.07	2.55	1.32
IP23	4.53	3.94	3.06	4.31	3.86	4.36	4.29	5.89	3.91	4.13	4.71	3.56	6.04	2.32
EA13/03	2.58	2.01	1.86	2.99	2.84	2.23	2.35	2.99	3.83	2.59	5.54	2.65	3.60	2.17
PA8	1.80	1.51	2.36	1.97	2.02	3.19	2.66	2.54	1.88	2.79	2.17	1.91	4.62	2.17
πTC13	3.93	4.37	6.24	7.89	8.37	8.76	8.12	8.34	6.69	7.85	5.14	5.32	11.45	4.84
HTBH38/08	1.89	2.01	2.36	3.71	4.86	6.87	7.71	6.09	4.15	6.96	1.44	1.31	5.26	1.25
NHTBH38/08	2.41	2.89	3.74	2.83	4.96	5.90	8.91	8.95	3.81	9.07	2.86	2.24	7.18	2.06
NCCE30	0.54	0.54	0.39	0.92	2.71	1.33	1.40	1.41	0.63	1.35	0.61	0.52	0.99	0.75
AE17	6.23	5.64	5.67	10.22	49.90	17.14	18.04	86.16	7.04	23.81	21.81	9.73	16.23	6.85
HC7/11	11.51	6.77	4.63	11.05	14.63	14.32	10.48	30.14	3.35	6.42	2.42	2.58	8.39	3.98
3dEE8	15.90	10.73	9.01	19.13	11.13	18.41	12.41	12.87	8.55	10.46	19.09	21.07	13.81	4.83
4dAEE5	6.30	9.34	7.57	6.03	4.30	11.91	5.19	7.07	6.58	5.11	11.04	11.05	9.45	0.88
pEE5	9.71	5.69	7.90	10.53	5.35	9.09	2.25	3.86	7.50	2.31	10.39	22.39	7.21	4.75
DC9/12	8.95	6.43	6.48	3.18	12.01	15.03	14.20	17.59	10.67	14.91	5.98	9.01	14.20	4.25
2pIsoE4	0.85	1.52	1.92	2.58	4.45	4.35	3.54	6.47	3.16	2.53	3.32	2.79	2.46	1.95
4pIsoE4	1.90	2.42	2.47	1.95	3.45	3.68	2.60	4.90	2.88	3.27	5.03	3.19	3.33	3.75
NGDWI21	0.10	0.03	0.16	0.26	0.21	0.15	0.17	0.16	0.13	0.17	0.57	0.37	0.08	0.02
MR-TMD-BE3	53.15	61.35	60.24	19.78	21.41	18.87	18.94	9.45	5.59	18.09	25.52	18.54	26.61	20.31
SMAE3	8.68	6.97	6.42	2.48	9.31	6.58	9.04	23.40	6.02	11.47	9.28	3.16	12.94	3.33
S6x6	0.82	1.07	0.31	2.52	7.84	4.17	3.38	3.52	0.80	3.19	1.51	1.13	1.82	1.72
ABDE13	0.81	1.11	1.50	1.92	9.00	9.73	10.67	14.60	5.35	9.01	3.36	1.96	6.61	4.62

Table S6. MUEs (kcal/mol) for the AME471 database and subdatabases: hybrid meta-GGAs (H meta-GGAs)

Type	mGGAh									
Functional	TPSSh	τ-HCTHhyb	BB1K	MPWB1K	MPW1B95	BMK	TPSS1KCIS	MPWKCIS1K	MPW1KCIS	PBE1KCIS	PWB6K
SR-MGM-BE9	3.34	2.41	4.31	3.78	2.65	2.55	4.25	6.25	4.05	3.64	3.85
SR-MGN-BE107	2.61	1.62	2.35	1.93	1.22	1.20	2.10	2.70	2.65	2.65	2.45
SR-TM-BE17	4.48	6.13	5.53	5.14	4.38	5.73	5.91	6.97	6.42	5.58	5.32
MR-MGM-BE4	6.93	5.72	8.89	19.99	6.73	9.56	9.39	17.67	10.28	15.85	13.2
MR-MGN-BE17	5.78	4.38	11.56	11.42	5.06	6.08	3.76	12.54	5.50	4.94	13.07
MR-TM-BE13	4.44	2.08	8.81	8.84	4.04	4.94	4.1	10.52	5.02	3.89	9.90
IsoL6/11	3.09	1.80	1.84	1.79	1.57	1.81	2.91	1.18	1.49	1.07	1.86
IP23	3.6	3.77	3.35	3.42	2.10	4.44	3.29	3.20	4.16	3.22	3.51
EA13/03	2.84	1.82	4.38	4.13	2.93	1.61	2.86	3.66	2.07	2.22	3.62
PA8	2.76	1.91	1.45	1.17	1.03	1.05	2.26	1.40	1.37	1.30	1.23
πTC13	8.06	7.45	5.99	5.68	5.38	4.58	7.49	6.11	5.55	5.03	5.85
HTBH38/08	5.96	5.28	1.18	1.3	3.01	1.27	4.69	1.63	5.86	5.13	1.28
NHTBH38/08	6.81	4.48	1.41	1.44	2.19	1.15	5.43	2.15	4.85	3.89	1.42
NCCE30	1.16	1.04	1.04	0.49	0.63	1.13	1.08	0.86	1.16	0.84	0.33
AE17	15.26	6.03	15.48	15.99	16.76	16.73	20.99	5.03	6.13	31.36	65.5
HC7/11	6.89	6.89	9.52	11.21	6.57	6.05	8.48	9.72	5.71	4.30	9.74
3dEE8	9.56	12.93	6.47	6.66	6.66	9.24	8.64	6.64	10.84	7.03	8.42
4dAEE5	5.13	7.92	8.62	4.63	4.59	5.93	5.27	10.02	9.48	9.30	4.83
pEE5	2.83	3.79	4.71	4.19	3.26	3.55	4.78	5.56	4.36	3.54	3.50
DC9/12	12.52	9.69	6.48	5.06	6.81	3.58	12.88	8.93	13.03	11.28	4.50
2pIsoE4	3.20	3.22	1.43	1.35	1.49	1.28	3.42	3.25	3.59	3.35	1.59
4pIsoE4	2.72	2.83	1.7	1.72	1.56	1.41	2.83	2.43	2.15	2.18	2.05
NGDWI21	0.17	0.17	0.35	0.14	0.15	0.68	0.14	0.17	0.19	0.10	0.11
MR-TMD-BE3	29.95	18.08	108.73	113.1	73.82	89.36	29.84	104.12	28.37	53.29	119.86
SMAE3	16.01	4.83	16.32	14.96	5.42	3.81	11.94	18.79	3.97	2.09	18.79
S6x6	3.58	2.92	2.70	1.71	2.08	2.71	3.34	3.12	3.83	3.27	1.16
ABDE13	9.88	4.85	1.66	1.18	1.20	1.21	8.33	4.04	5.44	3.46	1.33

Table S7. MUEs (kcal/mol) for the AME471 database and subdatabases: hybrid meta-GGAs (H meta-GGAs), RSH meta-GGA. NGA, GH-NGA

Type	mGGAh								mGGArsh	mNGArsh	mNGAh
Functional	PW6B95	M05	M05-2X	M06-HF	M06	M06-2X	M08-HX	M08-SO	M11	MN12-SX	MN15
SR-MGM-BE9	12.93	3.24	2.18	6.06	3.57	2.01	2.52	2.29	5.04	7.03	2.72
SR-MGN-BE107	1.27	1.57	1.07	1.46	1.25	0.98	2.13	1.91	1.09	1.09	0.87
SR-TM-BE17	3.90	4.97	7.21	11.93	5.30	7.29	5.15	5.62	8.41	10.36	4.02
MR-MGM-BE4	7.62	6.36	7.26	14.38	5.00	10.45	7.77	10.05	11.16	9.15	3.92
MR-MGN-BE17	4.70	4.87	7.26	11.20	4.12	5.74	6.62	6.17	6.95	4.44	2.78
MR-TM-BE13	3.83	3.47	9.82	20.46	2.76	11.42	8.74	7.66	6.41	8.13	3.69
IsoL6/11	2.03	2.75	1.22	2.46	1.27	1.53	0.59	1.19	1.10	1.21	1.80
IP23	3.16	4.76	5.13	7.44	4.99	3.31	4.06	3.61	7.96	6.07	2.80
EA13/03	1.83	2.97	2.04	3.31	1.85	2.14	1.32	2.72	0.89	2.11	0.88
PA8	1.16	2.27	1.43	2.28	1.84	1.65	1.08	1.64	1.03	1.16	1.14
πTC13	5.82	5.69	3.06	2.05	4.40	1.49	1.87	1.84	2.24	3.24	3.52
HTBH38/08	3.13	1.94	1.35	2.07	1.98	1.14	0.72	1.07	1.30	0.95	0.97
NHTBH38/08	2.83	2.07	1.81	2.53	2.33	1.22	1.22	1.23	1.28	1.35	1.74
NCCE30	0.60	0.57	0.32	0.44	0.46	0.29	0.36	0.38	0.29	0.33	0.32
AE17	98.53	10.65	10.09	12.42	4.45	2.14	4.10	3.76	8.88	4.52	7.38
HC7/11	4.15	7.71	3.64	2.29	2.78	2.15	4.89	4.60	3.74	2.21	3.72
3dEE8	7.65	11.42	12.31	22.59	10.83	8.95	7.92	5.22	13.06	24.83	8.17
4dAEE5	4.92	9.65	8.81	12.06	7.86	9.44	6.61	6.20	6.43	16.76	5.28
pEE5	2.05	8.17	10.09	8.40	5.23	4.55	1.51	5.05	5.02	7.71	4.26
DC9/12	7.87	8.86	2.32	4.03	2.75	4.11	4.92	4.76	2.96	4.93	5.81
2pIsoE4	2.05	2.55	2.30	2.09	1.60	1.77	1.48	0.83	1.91	1.99	0.27
4pIsoE4	1.95	1.12	3.48	4.59	2.31	2.71	2.06	1.46	2.53	2.74	1.96
NGDWI21	0.12	0.08	0.07	0.13	0.19	0.11	0.09	0.08	0.14	0.27	0.02
MR-TMD-BE3	65.80	35.73	117.87	141.20	36.12	119.39	126.44	119.83	82.58	22.42	22.22
SMAE3	7.47	3.97	9.13	21.89	1.88	8.08	11.26	11.59	7.87	2.73	0.53
S6x6	1.93	2.16	0.90	0.83	1.12	0.54	0.53	0.60	0.63	1.29	0.32
ABDE13	2.73	6.48	1.69	5.11	2.29	1.38	1.27	1.81	2.25	1.59	1.80

Table S8. MUE (kcal/mol) for intermolecular charge transfer database (CT7)

Functionals	CT7	Functionals	CT7
SOGGA11-X	0.21	O3LYP	1.18
MPWB1K	0.23	TPSS1KCIS	1.23
MN15-L	0.25	revPBE	1.28
MN15	0.25	HCTH407	1.31
MGGA_MS2	0.26	HSE	1.31
PWB6K	0.26	RPBE	1.35
ωB97X-D	0.28	SOGGA11	1.35
M11	0.30	τ-HCTHhyb	1.38
M06-HF	0.35	MPW3LYP	1.38
M06-2X	0.37	BPW91	1.39
MN12-SX	0.40	TPSSh	1.44
BMK	0.43	OLYP	1.55
MPW1K	0.44	OreLYP	1.57
M05-2X	0.45	BLYP	1.67
MPW1B95	0.48	τ-HCTH	1.78
MPWKCIS1K	0.48	M06-L	1.78
CAM-B3LYP	0.48	MOHLYP	1.80
B97-3	0.49	N12	1.87
B1LYP	0.49	B97-D	1.89
B97-2	0.55	BP86	2.04
ωB97	0.56	TPSS	2.22
M08-HX	0.59	mPWPW	2.26
B3PW91	0.63	B86PW91	2.26
mPW1PW	0.66	MPWLYP1M	2.29
M08-SO	0.66	revTPSS	2.36
M05	0.66	TPSSLYP1W	2.36
BB1K	0.67	PBELYP1W	2.44
ωB97X	0.67	MPWLYP1W	2.45
PW6B95	0.70	PBE1W	2.51
MN12-L	0.70	B86LYP	2.83
B3LYP	0.71	VSXC	2.86
GAM	0.75	BR89LYP	2.87
HFLYP	0.87	PBE	2.97
LC-ωPBE	0.90	B86P86	3.11
B98	0.92	PW91	3.35
M11-L	0.94	HFPW91	3.84
MPW1KCIS	0.95	PBEsol	4.21
N12-SX	0.96	SOGGA	4.29
PBE1KCIS	0.99	MOHLYP2	5.29
PBE0	1.06	SVWN5	6.79
M06	1.07	SVWN3	7.00
B97-1	1.18		

Table S9. MUE (kcal/mol) for Molecular Energy Databases and its subdatabases

Functionals[a]	MGBE150	TMBE33	BH76	NC87	EE18	IsoE14	HCTC20	AME454	AME471	MR54	SR313
GSVWN5	18.36	25.20	14.99	2.48	10.81	2.34	10.63	13.59	28.30	35.04	13.03
GSVWN3	20.33	27.79	15.08	2.64	9.25	2.37	11.25	15.39	26.02	38.42	15.02
SOGGA	8.63	15.00	11.28	1.61	8.08	1.88	8.90	7.90	17.83	22.32	7.22
PBEsol	8.81	14.50	11.28	1.63	8.81	1.80	7.39	7.88	16.47	21.92	7.25
SOGGA11	4.01	14.00	5.45	2.29	9.63	2.17	7.01	5.39	5.56	13.51	4.96
B86P86	4.67	27.10	7.86	2.00	8.22	2.40	8.95	6.80	7.63	20.52	5.84
B86LYP	4.48	25.41	7.02	2.39	9.73	3.92	13.47	6.72	7.38	20.95	5.55
BP86	5.55	9.05	8.94	2.43	7.32	2.71	7.29	6.13	6.52	14.06	5.86
BLYP	4.34	9.85	8.03	2.78	8.31	4.30	13.53	5.92	6.02	14.08	5.46
BR89LYP	4.10	6.55	6.82	1.96	8.74	4.82	13.99	5.44	6.27	11.33	5.43
B86PW91	4.33	7.41	6.23	2.51	7.12	2.34	9.33	4.96	5.68	9.76	4.89
PW91	5.04	10.26	9.20	1.49	7.41	2.38	5.32	5.70	5.66	14.25	5.46
BPW91	4.23	9.19	7.32	3.07	9.31	2.69	8.37	5.47	5.70	12.66	5.03
PBE	4.95	10.88	8.87	1.61	7.69	2.32	5.02	5.63	7.14	14.15	5.15
mPWPW	4.47	8.82	8.23	2.15	7.80	2.55	6.99	5.43	5.68	13.32	5.05
revPBE	4.25	7.64	6.70	3.04	7.15	2.85	9.43	5.26	5.47	11.44	4.88
RPBE	4.53	7.34	6.63	2.83	6.74	2.96	9.92	5.33	5.48	11.58	5.01
HCTH407	3.80	13.31	5.89	2.41	9.80	3.55	10.59	5.57	5.98	14.01	5.06
OLYP	3.75	8.42	5.44	4.32	7.56	3.22	11.32	5.25	5.43	11.03	4.59
MPWLYP1W	4.61	10.03	8.61	2.12	8.68	4.29	13.08	5.99	7.06	14.71	5.62
PBE1W	4.78	9.22	8.33	1.98	7.21	2.90	8.28	5.67	5.80	13.70	5.36
PBELYP1W	4.94	8.02	8.32	1.60	9.11	4.48	13.91	6.04	8.10	13.98	5.98
MOHLYP	7.20	10.14	5.65	5.03	7.32	3.86	13.26	7.04	16.03	19.76	5.45
B97-D	3.44	14.03	6.76	0.46	12.45	2.93	10.44	5.10	5.29	10.69	5.50
MOHLYP2	21.87	20.40	3.78	8.19	9.18	6.21	23.73	15.23	15.22	35.69	13.65
OreLYP	3.73	6.48	5.93	4.53	8.18	3.15	10.44	5.18	5.08	10.22	4.56
N12	3.42	9.36	6.90	2.30	16.22	2.21	7.09	5.05	5.38	9.35	5.20
GAM	2.97	6.24	5.25	1.14	6.93	3.29	7.77	4.17	4.39	9.45	4.11
VSXC	3.02	7.77	4.91	4.23	7.63	4.27	10.56	4.71	6.34	8.66	4.23
τ-HCTH	3.40	7.83	6.39	2.22	14.02	3.52	10.71	5.00	5.44	9.37	5.10
TPSS	3.47	8.36	8.31	1.92	7.58	3.32	8.95	4.98	5.46	9.48	4.97
TPSSLYP1W	5.20	6.65	7.52	1.98	8.76	5.70	15.97	6.00	8.89	12.16	6.11
M06-L	2.63	5.46	3.98	0.58	7.71	2.91	5.52	3.27	3.41	5.91	3.62
revTPSS	3.26	7.36	8.02	1.83	6.71	3.35	7.35	4.70	5.39	9.39	4.74
M11-L	2.81	7.02	2.15	0.97	14.44	3.06	4.19	3.45	4.11	6.74	3.71
MGGA_MS2	3.93	7.92	6.22	1.11	10.77	2.75	10.38	4.96	5.36	10.76	5.07
MN12-L	2.33	11.69	1.78	0.74	18.65	2.17	4.36	3.54	3.77	8.93	3.48
MN15-L	1.84	5.16	1.66	0.98	3.71	2.19	4.54	2.19	2.36	4.35	2.15
HFLYP	14.79	39.14	7.13	1.37	8.98	3.92	12.04	11.87	11.75	47.31	8.65
HFPW91	34.44	43.10	12.26	14.97	12.60	3.32	19.88	23.53	23.05	96.55	13.39
B3PW91	3.07	9.34	3.83	2.36	7.06	2.24	6.07	4.02	4.05	9.34	3.59
B3LYP	3.56	7.32	4.39	2.04	6.58	3.67	9.80	4.33	4.83	10.67	4.09
PBE0	2.82	10.18	3.83	1.33	6.85	1.87	7.26	3.72	4.98	10.37	3.28
mPW1PW	3.49	10.31	4.63	1.71	7.31	2.03	6.80	4.25	4.49	10.72	3.87
B1LYP	5.48	12.99	3.41	2.10	7.62	3.82	10.09	5.34	5.51	14.87	4.69
B98	2.71	6.92	3.74	1.51	7.16	2.55	7.60	3.50	3.55	8.26	3.28
B97-1	2.08	5.26	3.89	1.33	7.31	2.33	6.76	3.07	3.16	6.09	3.08
MPW1K	5.87	18.90	1.53	1.50	8.23	2.20	9.28	5.33	5.49	19.03	4.09
O3LYP	3.51	10.49	3.85	3.61	6.04	2.98	9.58	4.73	4.76	10.19	4.13
MPW3LYP	3.55	9.81	4.79	1.44	8.04	3.51	8.45	4.37	4.40	11.38	4.05
MPWLYP1M	3.82	6.27	7.83	1.87	8.18	4.07	11.68	5.08	5.33	10.90	5.03
B97-2	2.14	5.20	2.72	2.22	7.81	2.54	7.47	3.25	3.51	6.56	2.99
B97-3	2.50	9.85	1.83	2.10	6.20	2.56	7.44	3.44	3.57	9.92	2.70
SOGGA11-X	2.62	15.26	1.48	1.50	5.14	2.22	6.50	3.58	3.63	12.84	2.56

CAM-B3LYP	3.09	10.75	2.90	1.35	6.20	2.89	4.57	3.59	3.85	9.64	3.20
LC-ωPBE	3.47	12.43	1.77	1.56	8.46	1.55	8.96	4.02	4.79	11.81	3.39
HSE	2.97	9.92	3.98	1.31	8.09	1.99	6.60	3.85	4.89	10.18	3.54
ωB97	2.46	10.31	2.15	0.55	11.51	1.42	6.58	3.39	3.49	10.26	3.05
ωB97X	2.42	10.57	2.45	0.64	8.94	1.79	5.21	3.20	3.29	9.72	2.84
N12-SX	2.28	6.09	3.27	1.42	13.10	2.06	9.00	3.41	3.66	6.38	3.58
ωB97X-D	2.23	8.70	3.05	0.30	8.30	1.75	5.68	2.99	3.09	8.89	2.77
TPSSh	3.76	6.78	6.39	1.92	6.46	3.02	7.65	4.52	4.91	9.41	4.43
τ-HCTHhyb	2.37	5.62	4.88	1.61	9.00	2.50	7.25	3.57	3.66	6.38	3.68
BB1K	3.63	16.20	1.30	1.56	6.58	1.68	7.23	4.08	4.49	15.20	2.85
MPWB1K	3.53	16.41	1.37	0.91	5.41	1.64	7.62	3.88	4.31	16.17	2.58
MPW1B95	1.89	10.56	2.60	1.11	5.14	1.54	5.80	2.93	3.42	9.32	2.35
BMK	2.06	13.02	1.21	1.68	6.74	1.54	5.09	3.07	3.57	10.03	2.30
TPSS1KCIS	3.15	7.37	5.06	1.79	6.63	3.03	7.84	4.09	4.70	8.82	3.92
MPWKCIS1K	4.54	17.20	1.89	1.63	7.28	2.13	7.37	4.65	4.67	16.93	3.37
MPW1KCIS	3.50	7.86	5.36	2.03	8.66	2.28	5.61	4.25	4.32	8.81	4.09
PBE1KCIS	3.39	9.25	4.51	1.67	6.69	2.04	4.77	3.89	4.88	9.38	3.58
PWB6K	3.93	17.54	1.35	0.62	6.06	1.84	7.21	4.05	6.27	16.82	2.82
PW6B95	2.65	9.50	2.98	1.03	5.34	2.01	5.24	3.21	6.65	8.95	2.86
M05	2.60	7.18	2.01	1.11	10.03	2.23	6.40	3.24	3.51	7.24	3.16
M05-2X	2.06	18.30	1.58	0.50	10.72	2.17	3.26	3.46	3.70	13.25	2.61
M06-HF	3.50	27.04	2.30	0.53	15.72	2.96	2.13	5.15	5.42	19.90	4.01
M06	1.90	7.60	2.16	0.67	8.45	1.66	3.83	2.63	2.70	5.43	2.70
M06-2X	1.87	19.11	1.18	0.35	7.86	1.94	1.72	3.11	3.08	13.33	2.13
M08-HX	2.74	17.59	0.97	0.37	5.78	1.26	2.93	3.25	3.28	13.62	2.29
M08-SO	2.62	16.81	1.15	0.40	5.45	1.16	2.81	3.19	3.21	12.96	2.28
M11	2.36	14.36	1.29	0.39	8.99	1.74	2.77	3.20	3.41	10.35	2.80
MN12-SX	2.08	10.58	1.15	0.71	17.83	1.87	2.88	3.18	3.23	6.37	3.35
MN15	1.36	5.54	1.36	0.25	6.28	1.41	3.59	1.88	2.08	4.75	1.85

[a]The functionals are in the same order as in Table S1.

Table S10. The average and median mean unsigned errors for the molecular energy database and its subdatabases calculated by 83 density functionals

	MGBE150	TMBE33	BH76	EE18	IsoE14	HCTC20	AME471
Average MUE	4.68	12.02	5.02	8.52	2.69	8.11	6.21
Median MUE	3.50	9.85	4.63	7.86	2.40	7.44	4.98

	NC87	MR54	SR313	AME454	AE17	AME471
Average MUE	1.96	13.89	4.59	5.16	34.28	6.21
Median MUE	1.63	10.76	4.09	4.52	10.88	4.98

Table S11. MUE (kcal/mol) for Molecular Energy Databases and its subdatabases

Functionals[a]	MGBE150	TMBE33	BH76	NC87	EE18	IsoE14	HCTC20	AME454	AME471	MR54	SR313
MN15	1.36	5.54	1.36	0.25	6.04	1.41	3.59	1.87	2.07	4.75	1.84
MN15-L	1.84	5.16	1.66	0.98	3.71	2.19	4.54	2.19	2.36	4.35	2.15
M06	1.90	7.60	2.16	0.67	8.45	1.66	3.83	2.63	2.70	5.43	2.70
M06-2X	1.87	19.11	1.18	0.35	7.86	1.94	1.72	3.11	3.08	13.33	2.13
ωB97X-D	2.23	8.70	3.05	0.30	8.30	1.75	5.68	2.99	3.09	8.89	2.77
B97-1	2.08	5.26	3.89	1.33	7.31	2.33	6.76	3.07	3.16	6.09	3.08
M08-SO	2.62	16.81	1.15	0.40	5.45	1.16	2.81	3.19	3.21	12.96	2.28
MN12-SX	2.08	10.58	1.15	0.71	17.83	1.87	2.88	3.18	3.23	6.37	3.35
M08-HX	2.74	17.59	0.97	0.37	5.78	1.26	2.93	3.25	3.28	13.62	2.29
ωB97X	2.42	10.57	2.45	0.64	8.94	1.79	5.21	3.20	3.29	9.72	2.84
M11	2.36	14.36	1.29	0.39	8.99	1.74	2.77	3.20	3.41	10.35	2.80
M06-L	2.63	5.46	3.98	0.58	7.71	2.91	5.52	3.27	3.41	5.91	3.62
MPW1B95	1.89	10.56	2.60	1.11	5.14	1.54	5.80	2.93	3.42	9.32	2.35
ωB97	2.46	10.31	2.15	0.55	11.51	1.42	6.58	3.39	3.49	10.26	3.05
M05	2.60	7.18	2.01	1.11	10.03	2.23	6.40	3.24	3.51	7.24	3.16
B97-2	2.14	5.20	2.72	2.22	7.81	2.54	7.47	3.25	3.51	6.56	2.99
B98	2.71	6.92	3.74	1.51	7.16	2.55	7.60	3.50	3.55	8.26	3.28
B97-3	2.50	9.85	1.83	2.10	6.20	2.56	7.44	3.44	3.57	9.92	2.70
BMK	2.06	13.02	1.21	1.68	6.74	1.54	5.09	3.07	3.57	10.03	2.30
SOGGA11-X	2.62	15.26	1.48	1.50	5.14	2.22	6.50	3.58	3.63	12.84	2.56
N12-SX	2.28	6.09	3.27	1.42	13.10	2.06	9.00	3.41	3.66	6.38	3.58
τ-HCTHhyb	2.37	5.62	4.88	1.61	9.00	2.50	7.25	3.57	3.66	6.38	3.68
M05-2X	2.06	18.30	1.58	0.50	10.72	2.17	3.26	3.46	3.70	13.25	2.61
MN12-L	2.33	11.69	1.78	0.74	18.65	2.17	4.36	3.54	3.77	8.93	3.48
CAM-B3LYP	3.09	10.75	2.90	1.35	6.20	2.89	4.57	3.59	3.85	9.64	3.20
B3PW91	3.07	9.34	3.83	2.36	7.06	2.24	6.07	4.02	4.05	9.34	3.59
M11-L	2.81	7.02	2.15	0.97	14.44	3.06	4.19	3.45	4.11	6.74	3.71
MPWB1K	3.53	16.41	1.37	0.91	5.41	1.64	7.62	3.88	4.31	16.17	2.58
MPW1KCIS	3.50	7.86	5.36	2.03	8.66	2.28	5.61	4.25	4.32	8.81	4.09
GAM	2.97	6.24	5.25	1.14	6.93	3.29	7.77	4.17	4.39	9.45	4.11
MPW3LYP	3.55	9.81	4.79	1.44	8.04	3.51	8.45	4.37	4.40	11.38	4.05
mPW1PW	3.49	10.31	4.63	1.71	7.31	2.03	6.80	4.25	4.49	10.72	3.87
BB1K	3.63	16.20	1.30	1.56	6.58	1.68	7.23	4.08	4.49	15.20	2.85
MPWKCIS1K	4.54	17.20	1.89	1.63	7.28	2.13	7.37	4.65	4.67	16.93	3.37
TPSS1KCIS	3.15	7.37	5.06	1.79	6.63	3.03	7.84	4.09	4.70	8.82	3.92
O3LYP	3.51	10.49	3.85	3.61	6.04	2.98	9.58	4.73	4.76	10.19	4.13
LC-ωPBE	3.47	12.43	1.77	1.56	8.46	1.55	8.96	4.02	4.79	11.81	3.39
PBE1KCIS	3.39	9.25	4.51	1.67	6.69	2.04	4.77	3.89	4.88	9.38	3.58
HSE	2.97	9.92	3.98	1.31	8.09	1.99	6.60	3.85	4.89	10.18	3.54
TPSSh	3.76	6.78	6.39	1.92	6.46	3.02	7.65	4.52	4.91	9.41	4.43
B3LYP	3.56	7.32	4.39	2.04	6.58	3.67	9.80	4.33	4.83	10.67	4.09
PBE0	2.82	10.18	3.83	1.33	6.85	1.87	7.26	3.72	4.98	10.37	3.28
OreLYP	3.73	6.48	5.93	4.53	8.18	3.15	10.44	5.18	5.08	10.22	4.56
B97-D	3.44	14.03	6.76	0.46	12.45	2.93	10.44	5.10	5.29	10.69	5.50
MPWLYP1M	3.82	6.27	7.83	1.87	8.18	4.07	11.68	5.08	5.33	10.90	5.03
TPSS	3.47	8.36	8.31	1.92	7.58	3.32	8.95	4.98	5.46	9.48	4.97
MGGA_MS2	3.93	7.92	6.22	1.11	10.77	2.75	10.38	4.96	5.36	10.76	5.07
N12	3.42	9.36	6.90	2.30	16.22	2.21	7.09	5.05	5.38	9.35	5.20
revTPSS	3.26	7.36	8.02	1.83	6.71	3.35	7.35	4.70	5.39	9.39	4.74
M06-HF	3.50	27.04	2.30	0.53	15.72	2.96	2.13	5.15	5.42	19.90	4.01
OLYP	3.75	8.42	5.44	4.32	7.56	3.22	11.32	5.25	5.43	11.03	4.59
τ-HCTH	3.40	7.83	6.39	2.22	14.02	3.52	10.71	5.00	5.44	9.37	5.10
revPBE	4.25	7.64	6.70	3.04	7.15	2.85	9.43	5.26	5.47	11.44	4.88
RPBE	4.53	7.34	6.63	2.83	6.74	2.96	9.92	5.33	5.48	11.58	5.01

MPW1K	5.87	18.90	1.53	1.50	8.23	2.20	9.28	5.33	5.49	19.03	4.09
B1LYP	5.48	12.99	3.41	2.10	7.62	3.82	10.09	5.34	5.51	14.87	4.69
SOGGA11	4.01	14.00	5.45	2.29	9.63	2.17	7.01	5.39	5.56	13.51	4.96
PW91	5.04	10.26	9.20	1.49	7.41	2.38	5.32	5.70	5.66	14.25	5.46
B86PW91	4.33	7.41	6.23	2.51	7.12	2.34	9.33	4.96	5.68	9.76	4.89
mPWPW	4.47	8.82	8.23	2.15	7.80	2.55	6.99	5.43	5.68	13.32	5.05
BPW91	4.23	9.19	7.32	3.07	9.31	2.69	8.37	5.47	5.70	12.66	5.03
PBE1W	4.78	9.22	8.33	1.98	7.21	2.90	8.28	5.67	5.80	13.70	5.36
HCTH407	3.80	13.31	5.89	2.41	9.80	3.55	10.59	5.57	5.98	14.01	5.06
BLYP	4.34	9.85	8.03	2.78	8.31	4.30	13.53	5.92	6.02	14.08	5.46
PWB6K	3.93	17.54	1.35	0.62	6.06	1.84	7.21	4.05	6.27	16.82	2.82
BR89LYP	4.10	6.55	6.82	1.96	8.74	4.82	13.99	5.44	6.27	11.33	5.43
VSXC	3.02	7.77	4.91	4.23	7.63	4.27	10.56	4.71	6.34	8.66	4.23
BP86	5.55	9.05	8.94	2.43	7.32	2.71	7.29	6.13	6.52	14.06	5.86
PW6B95	2.65	9.50	2.98	1.03	5.34	2.01	5.24	3.21	6.65	8.95	2.86
PBE	4.95	10.88	8.87	1.61	7.69	2.32	5.02	5.63	7.14	14.15	5.15
MPWLYP1W	4.61	10.03	8.61	2.12	8.68	4.29	13.08	5.99	7.06	14.71	5.62
B86LYP	4.48	25.41	7.02	2.39	9.73	3.92	13.47	6.72	7.38	20.95	5.55
B86P86	4.67	27.10	7.86	2.00	8.22	2.40	8.95	6.80	7.63	20.52	5.84
PBELYP1W	4.94	8.02	8.32	1.60	9.11	4.48	13.91	6.04	8.10	13.98	5.98
TPSSLYP1W	5.20	6.65	7.52	1.98	8.76	5.70	15.97	6.00	8.89	12.16	6.11
HFLYP	14.79	39.14	7.13	1.37	8.98	3.92	12.04	11.87	11.75	47.31	8.65
MOHLYP2	21.87	20.40	3.78	8.19	9.18	6.21	23.73	15.23	15.22	35.69	13.65
MOHLYP	7.20	10.14	5.65	5.03	7.32	3.86	13.26	7.04	16.03	19.76	5.45
PBEsol	8.81	14.50	11.28	1.63	8.81	1.80	7.39	7.88	16.47	21.92	7.25
SOGGA	8.63	15.00	11.28	1.61	8.08	1.88	8.90	7.90	17.83	22.32	7.22
HFPW91	34.44	43.10	12.26	14.97	12.60	3.32	19.88	23.53	23.05	96.55	13.39
GSVWN3	20.33	27.79	15.08	2.64	9.25	2.37	11.25	15.39	26.02	38.42	15.02
GSVWN5	18.36	25.20	14.99	2.48	10.81	2.34	10.63	13.59	28.30	35.04	13.03

[a]The functionals are in the order of increase MUE of AME471

Table S12. MUE (kcal/mol) for the Molecular Structure Database (MS10)

Functionals[a]	DG4H	DG6L	MS10	Functionals	DG4H	DG6L	MS10
GKSVWN5	0.031	0.011	0.019	PBE0	0.014	0.003	0.007
GKSVWN3	0.034	0.012	0.021	mPW1PW	0.015	0.003	0.008
SOGGA	0.013	0.009	0.011	B1LYP	0.032	0.009	0.018
PBEsol	0.011	0.010	0.010	B98	0.026	0.007	0.015
SOGGA11	0.054	0.008	0.026	B97-1	0.028	0.006	0.015
B86P86	0.029	0.015	0.021	MPW1K	0.012	0.011	0.011
B86LYP	0.045	0.024	0.032	O3LYP	0.030	0.004	0.014
BP86	0.021	0.015	0.017	B97-2	0.023	0.002	0.010
BLYP	0.037	0.019	0.026	MPW3LYP	0.026	0.009	0.016
BR89LYP	0.046	0.021	0.031	MPWLYP1M	0.031	0.018	0.023
B86PW91	0.031	0.014	0.021	B97-3	0.034	0.004	0.016
PW91	0.019	0.012	0.015	SOGGA11-X	0.021	0.004	0.011
BPW91	0.022	0.013	0.017	CAM-B3LYP	0.011	0.008	0.009
PBE	0.020	0.013	0.016	LC-wPBE	0.011	0.013	0.012
mPWPW	0.021	0.012	0.016	HSE	0.016	0.003	0.008
revPBE	0.034	0.015	0.023	ωB97	0.018	0.011	0.014
RPBE	0.038	0.016	0.025	ωB97X	0.017	0.008	0.012
HCTH407	0.033	0.004	0.016	N12-SX	0.012	0.005	0.008
OLYP	0.036	0.009	0.020	ωB97X-D	0.023	0.005	0.012
MPWLYP1W	0.037	0.020	0.027	TPSSh	0.013	0.006	0.009
PBE1W	0.028	0.014	0.020	τ-HCTHhyb	0.017	0.006	0.010
PBELYP1W	0.043	0.020	0.029	BB1K	0.011	0.009	0.010
MOHLYP	0.054	0.022	0.035	MPWB1K	0.010	0.012	0.011
B97-D	0.043	0.013	0.025	MPW1B95	0.011	0.005	0.007
MOHLYP2	0.092	0.022	0.050	BMK	0.028	0.007	0.015
OreLYP	0.034	0.011	0.020	TPSS1KCIS	0.019	0.005	0.011
N12	0.007	0.008	0.008	MPWKCIS1K	0.019	0.010	0.014
GAM	0.034	0.007	0.018	MPW1KCIS	0.025	0.006	0.014
VSXC	0.021	0.006	0.012	PBE1KCIS	0.024	0.003	0.011
τ-HCTH	0.019	0.006	0.011	PWB6K	0.012	0.013	0.013
TPSS	0.014	0.010	0.012	PW6B95	0.015	0.004	0.008
TPSSLYP1W	0.031	0.017	0.023	M05	0.031	0.007	0.017
M06-L	0.008	0.006	0.007	M05-2X	0.032	0.006	0.016
revTPSS	0.021	0.011	0.015	M06-HF	0.044	0.013	0.025
M11-L	0.022	0.012	0.016	M06	0.023	0.006	0.013
MGGA_MS2	0.008	0.007	0.007	M06-2X	0.049	0.004	0.022
MN12-L	0.022	0.005	0.012	M08-HX	0.047	0.005	0.022
MN15-L	0.014	0.004	0.008	M08-SO	0.027	0.007	0.015
HFLYP	0.031	0.046	0.040	M11	0.017	0.007	0.011
HFPW91	0.029	0.050	0.042	MN12-SX	0.017	0.003	0.009
B3PW91	0.017	0.004	0.009	MN15	0.008	0.005	0.006
B3LYP	0.027	0.009	0.016				

[a]The functionals listed are in the same order as in Table S1.

Table S13. MUE (kcal/mol) for the Molecular Structure Database (MS10)

Functionals[a]	DG4H	DG6L	MS10	Functionals	DG4H	DG6L	MS10
MN15	0.008	0.005	0.006	B97-1	0.028	0.006	0.015
M06-L	0.008	0.006	0.007	BMK	0.028	0.007	0.015
MGGA_MS2	0.008	0.007	0.007	M08-SO	0.027	0.007	0.015
PBE0	0.014	0.003	0.007	PBE	0.020	0.013	0.016
MPW1B95	0.011	0.005	0.007	HCTH407	0.033	0.004	0.016
N12	0.007	0.008	0.008	mPWPW	0.021	0.012	0.016
MN15-L	0.014	0.004	0.008	M11-L	0.022	0.012	0.016
mPW1PW	0.015	0.003	0.008	B3LYP	0.027	0.009	0.016
HSE	0.016	0.003	0.008	MPW3LYP	0.026	0.009	0.016
N12-SX	0.012	0.005	0.008	B97-3	0.034	0.004	0.016
PW6B95	0.015	0.004	0.008	M05-2X	0.032	0.006	0.016
B3PW91	0.017	0.004	0.009	BP86	0.021	0.015	0.017
CAM-B3LYP	0.011	0.008	0.009	BPW91	0.022	0.013	0.017
TPSSh	0.013	0.006	0.009	M05	0.031	0.007	0.017
MN12-SX	0.017	0.003	0.009	GAM	0.034	0.007	0.018
PBEsol	0.011	0.010	0.010	B1LYP	0.032	0.009	0.018
B97-2	0.023	0.002	0.010	GKSVWN5	0.031	0.011	0.019
τ-HCTHhyb	0.017	0.006	0.010	OLYP	0.036	0.009	0.020
BB1K	0.011	0.009	0.010	PBE1W	0.028	0.014	0.020
SOGGA	0.013	0.009	0.011	OreLYP	0.034	0.011	0.020
τ-HCTH	0.019	0.006	0.011	GKSVWN3	0.034	0.012	0.021
MPW1K	0.012	0.011	0.011	B86P86	0.029	0.015	0.021
SOGGA11-X	0.021	0.004	0.011	B86PW91	0.031	0.014	0.021
MPWB1K	0.010	0.012	0.011	M06-2X	0.049	0.004	0.022
TPSS1KCIS	0.019	0.005	0.011	M08-HX	0.047	0.005	0.022
PBE1KCIS	0.024	0.003	0.011	revPBE	0.034	0.015	0.023
M11	0.017	0.007	0.011	TPSSLYP1W	0.031	0.017	0.023
VSXC	0.021	0.006	0.012	MPWLYP1M	0.031	0.018	0.023
TPSS	0.014	0.010	0.012	RPBE	0.038	0.016	0.025
MN12-L	0.022	0.005	0.012	B97-D	0.043	0.013	0.025
LC-wPBE	0.011	0.013	0.012	M06-HF	0.044	0.013	0.025
ωB97X	0.017	0.008	0.012	SOGGA11	0.054	0.008	0.026
ωB97X-D	0.023	0.005	0.012	BLYP	0.037	0.019	0.026
PWB6K	0.012	0.013	0.013	MPWLYP1W	0.037	0.020	0.027
M06	0.023	0.006	0.013	PBELYP1W	0.043	0.020	0.029
O3LYP	0.030	0.004	0.014	BR89LYP	0.046	0.021	0.031
ωB97	0.018	0.011	0.014	B86LYP	0.045	0.024	0.032
MPWKCIS1K	0.019	0.010	0.014	MOHLYP	0.054	0.022	0.035
MPW1KCIS	0.025	0.006	0.014	HFLYP	0.031	0.046	0.040
PW91	0.019	0.012	0.015	HFPW91	0.029	0.050	0.042
revTPSS	0.021	0.011	0.015	MOHLYP2	0.092	0.022	0.050
B98	0.026	0.007	0.015				

[a]The functionals listed are in the order of increasing MUE of MS10

Table S14. Geometries (in Å), charge, and multiplicity of species in several databases: alkyl bond dissociation energies (ABDE13), six dimers at six intermonomeric distances (S6x6), semiconductor band gaps (SBG31), and excitation energies of selected organic molecules (EE69)

ABDE13

butane				
	0	1		
	6	-0.928482	1.410876	0.001213
	6	0.586226	1.393089	0.002419
	1	-1.325379	2.425099	0.001662
	1	-1.329631	0.903049	-0.876909
	1	-1.331099	0.901704	0.877875
	6	1.170559	-0.009541	-0.000211
	1	0.964525	1.94072	-0.866061
	1	0.963033	1.936803	0.874005
	6	2.68526	-0.027337	0.000687
	1	0.792474	-0.557052	0.868435
	1	0.793529	-0.553314	-0.871656
	1	3.082164	-1.041557	-0.001058
	1	3.087694	0.482886	-0.875454
	1	3.08662	0.479435	0.87932
c3h6				
	0	1		
	6	-2.213267	1.521683	0.026767
	1	-2.534248	2.309355	-0.657553
	1	-2.615002	0.576378	-0.334569
	1	-2.683762	1.743549	0.986643
	6	-0.733774	1.480744	0.147551
	6	0.031525	0.439071	-0.141552
	1	-0.259361	2.39089	0.505652
	1	1.106943	0.470927	-0.031228
	1	-0.389811	-0.492653	-0.501783

ch2chch2ch2

0	2		
6	2.513982	-0.250375	-0.295934
6	1.482785	0.336378	0.292951
1	3.480077	0.230366	-0.370971
1	2.432749	-1.24237	-0.725684
1	1.600633	1.335169	0.704616
6	0.106889	-0.252384	0.412276
6	-0.896292	0.558103	-0.321381
1	-0.168588	-0.320092	1.467981
1	0.130613	-1.276718	0.028186
1	-1.518495	1.284484	0.182929
1	-0.8872	0.579974	-1.402943

ch2chch2

0	2		
6	2.506597	-0.193407	-0.367482
6	1.434894	0.240846	0.377821
1	3.389989	0.413867	-0.503891
1	2.498586	-1.165757	-0.844876
1	1.506087	1.226516	0.82946
6	0.279877	-0.474051	0.59625
1	-0.530516	-0.079588	1.19245
1	0.145809	-1.462726	0.174454

ch2chchch2ch3

0	2		
6	-1.94513	0.93024	-0.034904
1	-1.297993	0.412352	-0.731786
1	-3.005829	0.889974	-0.237078
6	-1.446735	1.604426	1.04933
6	-0.106833	1.712848	1.391443
1	-2.159234	2.097973	1.703228
1	0.151386	2.273896	2.284018
6	1.012184	1.103577	0.621485
6	2.38335	1.447258	1.168938
1	0.942277	1.409191	-0.428893
1	0.885785	0.014298	0.594468
1	3.178114	0.985385	0.586139
1	2.552744	2.523978	1.161466
1	2.49256	1.109416	2.199568

ch2ch

0	2		
6	2.502688	-0.208688	-0.302027
6	1.498909	0.396916	0.259687
1	3.480137	0.259571	-0.421973
1	2.422208	-1.220921	-0.687327
1	1.308258	1.358232	0.708837

ch3

0	2		
6	-1.273369	0.742196	-0.217501
1	-0.765914	-0.156538	0.092307
1	-0.765894	1.459855	-0.840918
1	-2.28985	0.921076	0.092307

ch3oco

0	2		
6	-0.657339	0.161134	-0.000029
8	-0.703403	1.347083	-0.00013
8	-1.611924	-0.735153	0.000126
6	-2.966686	-0.202337	0.000154
1	-3.623447	-1.064623	0.001145
1	-3.123468	0.404176	0.889298
1	-3.124106	0.402576	-0.889968

chchch2ch3

0	2		
6	1.896141	-0.029947	-0.336025
6	0.791764	-0.14031	0.350007
1	2.881819	-0.464537	-0.304987
1	0.759347	-0.83962	1.19256
6	-0.48429	0.598168	0.08652
6	-1.655563	-0.336905	-0.165507
1	-0.344787	1.273237	-0.758664
1	-0.707708	1.231069	0.950709
1	-2.580419	0.217792	-0.315916
1	-1.486574	-0.950834	-1.049405
1	-1.809993	-1.013146	0.675738

hcoo

0	2		
6	-2.601901	1.269372	-0.016199
8	-1.353352	1.263053	-0.016452
8	-3.083478	2.421023	-0.015952
1	-3.214168	0.35471	-0.016149

hexene

0	1		
6	2.775589	-0.346686	-0.122933
6	1.452569	-0.32747	0.629207
1	3.34058	0.569663	0.049988
1	3.401163	-1.182486	0.18772
1	2.616239	-0.429717	-1.197689
1	0.914187	-1.260154	0.455665
1	1.658835	-0.298898	1.704048
6	0.607652	0.847686	0.26793
6	-0.60859	0.846601	-0.270736
1	1.067994	1.813688	0.461946
1	-1.069333	1.811807	-0.467736
6	-1.452999	-0.330019	-0.628417
6	-2.776046	-0.347456	0.123711
1	-0.914236	-1.261936	-0.45196
1	-1.65922	-0.304866	-1.703353
1	-3.401256	-1.184474	-0.184385
1	-2.61672	-0.427094	1.198727
1	-3.341411	0.568118	-0.052074

mb

0	1		
6	-5.65074	1.129488	-0.048426
6	-4.140196	1.036621	0.018568
1	-6.040176	0.628813	-0.939199
1	-6.118332	0.603684	0.788655
6	-3.648172	-0.3957	0.020929
1	-3.711582	1.585207	-0.821992
1	-3.789859	1.56121	0.909219
1	-2.561857	-0.446777	0.068792
1	-4.039005	-0.951324	0.874433
1	-3.960611	-0.927296	-0.878923
6	-6.161979	2.540342	-0.051048
8	-5.487943	3.535798	-0.005642
8	-7.51015	2.559874	-0.112332
6	-8.089029	3.863014	-0.119431
1	-9.163204	3.715922	-0.1692
1	-7.824578	4.410031	0.784498
1	-7.746327	4.435117	-0.980556

mf

0	1		
6	-2.501938	1.226371	-0.016195
8	-1.320957	1.034671	-0.016142
8	-3.102706	2.421087	-0.016236
1	-3.277874	0.445166	-0.016157
6	-2.208181	3.538038	-0.016264
1	-2.833242	4.424561	-0.01645
1	-1.573549	3.521174	0.86821
1	-1.573321	3.520952	-0.90057

pentene

0	1		
6	-4.74352	2.259537	0.437271
6	-3.660756	2.071534	-0.301942
1	-5.339084	3.159608	0.364659
1	-5.081759	1.513389	1.14754
1	-3.359835	2.852084	-0.99799
6	-2.779585	0.872125	-0.260052
6	-1.332277	1.200881	0.092731
1	-2.79296	0.377148	-1.238045
1	-3.178513	0.143641	0.451106
6	-0.433859	-0.018409	0.086861
1	-0.952972	1.946865	-0.611256
1	-1.305026	1.683218	1.072972
1	0.594074	0.234067	0.342604
1	-0.775325	-0.765739	0.804112
1	-0.420143	-0.496111	-0.893621

R1_oc=och2ch2ch3

0	2		
6	0.725948	-0.574955	0.000031
6	1.877482	0.416106	-0.000504
1	0.77151	-1.239782	-0.866937
1	0.771642	-1.238909	0.867672
6	3.225933	-0.274045	0.000267
1	1.788489	1.070383	-0.869647
1	1.788164	1.071697	0.867612
1	4.041532	0.446264	-0.000266
1	3.348599	-0.907986	0.878967
1	3.348817	-0.909531	-0.877285
6	-0.618419	0.061543	-0.000202
8	-0.816041	1.300429	0.000035
8	-1.70118	-0.575127	-0.000079

R3_ch3ch2ch2

0	2		
6	0.824777	-0.662196	-0.000008
6	1.901391	0.35202	-0.000535
1	0.49461	-1.124722	-0.92102
1	0.494497	-1.123611	0.921522
6	3.303116	-0.259952	0.000277
1	1.802231	1.001319	-0.874214
1	1.801818	1.002661	0.872102
1	4.07343	0.511295	-0.000107
1	3.457381	-0.885787	0.878801
1	3.457823	-0.887106	-0.877227

R4_ch3ch2

0	2		
6	2.062986	0.609261	-0.000591
6	3.251699	-0.260586	0.000335
1	1.661019	1.006973	-0.922101
1	1.66087	1.008741	0.920089
1	4.188885	0.311584	-0.000244
1	3.289952	-0.900677	0.882293
1	3.289987	-0.902504	-0.880294

R4_ch3oc=och2

0	2		
6	0.552927	-0.758415	-0.010219
1	0.571324	-1.776966	-0.368625
1	1.47441	-0.259591	0.245791
6	-0.682113	-0.038159	0.138083
8	-0.784949	1.104345	0.532865
8	-1.74748	-0.799312	-0.222572
6	-3.005185	-0.145109	-0.09957
1	-3.752917	-0.865008	-0.416773
1	-3.186157	0.158912	0.931069
1	-3.044559	0.743288	-0.729348

R5_ch3oc=och2ch2

0	2		
6	0.674387	-0.448321	-0.000125
6	1.789643	0.51663	-0.000663
1	0.710236	-1.11217	-0.867614
1	0.710472	-1.111453	0.86791
1	2.128108	0.966574	-0.92265
1	2.128028	0.967675	0.920814

6	-0.697488	0.197738	-0.000176
8	-0.925163	1.376763	-0.000472
8	-1.656165	-0.750354	0.000189
6	-2.991927	-0.248458	0.00023
1	-3.640881	-1.118349	0.000987
1	-3.176098	0.361262	0.883585
1	-3.176597	0.360059	-0.883854

S6x6

04_Water-Peptide_0.90				
	0	1		
	O	-0.392018453	-0.384718737	0.076071325
	H	-0.911460851	0.41381204	0.177648774
	H	0.52490382	-0.068484694	0.090511364
	C	2.05149617	-2.260917624	-0.227813599
	H	2.701459013	-3.122029501	-0.360728986
	H	1.3705202	-2.183445565	-1.071678874
	H	1.438479272	-2.399713611	0.659194764
	C	2.806228248	-0.962904747	-0.095220092
	O	2.229512801	0.112390109	0.06136865
	N	4.156861371	-1.060407431	-0.159838061
	H	4.557812999	-1.97094141	-0.289353159
	C	5.025103483	0.091563029	-0.050394979
	H	4.388609353	0.959863472	0.084389628
	H	5.69069299	0.000107831	0.805697902
	H	5.619569206	0.220983518	-0.952654166
04_Water-Peptide_0.95				
	0	1		
	O	-0.392018453	-0.384718737	0.076071325
	H	-0.911460851	0.41381204	0.177648774
	H	0.52490382	-0.068484694	0.090511364
	C	2.146191145	-2.25086961	-0.229432545
	H	2.796153988	-3.111981487	-0.362347932
	H	1.465215175	-2.173397551	-1.07329782
	H	1.533174247	-2.389665597	0.657575818
	C	2.900923223	-0.952856733	-0.096839038
	O	2.324207776	0.122438123	0.059749704
	N	4.251556346	-1.050359417	-0.161457007
	H	4.652507974	-1.960893396	-0.290972105
	C	5.119798458	0.101611043	-0.052013925
	H	4.483304328	0.969911486	0.082770682
	H	5.785387965	0.010155845	0.804078956
	H	5.714264181	0.231031532	-0.954273112
04_Water-Peptide_1.00				
	0	1		
	O	-0.392018453	-0.384718737	0.076071325
	H	-0.911460851	0.41381204	0.177648774
	H	0.52490382	-0.068484694	0.090511364

C	2.240786693	-2.240832146	-0.23104979
H	2.890749536	-3.101944023	-0.363965177
H	1.559810723	-2.163360087	-1.074915065
H	1.627769795	-2.379628133	0.655958573
C	2.995518771	-0.942819269	-0.098456283
O	2.418803324	0.132475587	0.058132459
N	4.346151894	-1.040321953	-0.163074252
H	4.747103522	-1.950855932	-0.29258935
C	5.214394006	0.111648507	-0.05363117
H	4.577899876	0.97994895	0.081153437
H	5.879983513	0.020193309	0.802461711
H	5.808859729	0.241068996	-0.955890357

04_Water-Peptide_1.05

0	1		
O	-0.392018453	-0.384718737	0.076071325
H	-0.911460851	0.41381204	0.177648774
H	0.52490382	-0.068484694	0.090511364
C	2.335581096	-2.230773582	-0.232670435
H	2.985543939	-3.091885459	-0.365585822
H	1.654605126	-2.153301523	-1.07653571
H	1.722564198	-2.369569569	0.654337928
C	3.090313174	-0.932760705	-0.100076928
O	2.513597727	0.142534151	0.056511814
N	4.440946297	-1.030263389	-0.164694897
H	4.841897925	-1.940797368	-0.294209995
C	5.309188409	0.121707071	-0.055251815
H	4.672694279	0.990007514	0.079532792
H	5.974777916	0.030251873	0.800841066
H	5.903654132	0.25112756	-0.957511002

04_Water-Peptide_1.10

0	1		
O	-0.392018453	-0.384718737	0.076071325
H	-0.911460851	0.41381204	0.177648774
H	0.52490382	-0.068484694	0.090511364
C	2.430229786	-2.220730479	-0.234288589
H	3.080192629	-3.081842356	-0.367203976
H	1.749253816	-2.14325842	-1.078153864
H	1.817212888	-2.359526466	0.652719774
C	3.184961864	-0.922717602	-0.101695082
O	2.608246417	0.152577254	0.05489366
N	4.535594987	-1.020220286	-0.166313051

H	4.936546615	-1.930754265	-0.295828149
C	5.403837099	0.131750174	-0.056869969
H	4.767342969	1.000050617	0.077914638
H	6.069426606	0.040294976	0.799222912
H	5.998302822	0.261170663	-0.959129156

04_Water-Peptide_1.25

0	1		
O	-0.392018453	-0.384718737	0.076071325
H	-0.911460851	0.41381204	0.177648774
H	0.52490382	-0.068484694	0.090511364
C	2.714360996	-2.190581526	-0.239146216
H	3.364323839	-3.051693403	-0.372061603
H	2.033385026	-2.113109467	-1.083011491
H	2.101344098	-2.329377513	0.647862147
C	3.469093074	-0.892568649	-0.106552709
O	2.892377627	0.182726207	0.050036033
N	4.819726197	-0.990071333	-0.171170678
H	5.220677825	-1.900605312	-0.300685776
C	5.687968309	0.161899127	-0.061727596
H	5.051474179	1.03019957	0.073057011
H	6.353557816	0.070443929	0.794365285
H	6.282434032	0.291319616	-0.963986783

04_Water-Peptide_A

0	1		
O	-0.392018453	-0.384718737	0.076071325
H	-0.911460851	0.41381204	0.177648774
H	0.52490382	-0.068484694	0.090511364

04_Water-Peptide_B

0	1		
C	2.05149617	-2.260917624	-0.227813599
H	2.701459013	-3.122029501	-0.360728986
H	1.3705202	-2.183445565	-1.071678874
H	1.438479272	-2.399713611	0.659194764
C	2.806228248	-0.962904747	-0.095220092
O	2.229512801	0.112390109	0.06136865
N	4.156861371	-1.060407431	-0.159838061
H	4.557812999	-1.97094141	-0.289353159
C	5.025103483	0.091563029	-0.050394979
H	4.388609353	0.959863472	0.084389628
H	5.69069299	0.000107831	0.805697902

H	5.619569206	0.220983518	-0.952654166

17_Uracil-Uracil_BP_0.90

0	1		
N	-0.729999134	0.022767626	0.000914648
H	0.298422555	0.074004465	0.001623038
C	-1.296824528	-1.24042682	0.001502336
O	-0.594098857	-2.253517508	0.002633705
C	-2.743622291	-1.262331701	0.000479378
H	-3.249590454	-2.211835169	0.000833106
C	-3.422019974	-0.095909207	-0.00092259
H	-4.500897091	-0.049216027	-0.001745463
N	-2.774836844	1.105408947	-0.001418075
H	-3.283838068	1.973877391	-0.002485743
C	-1.391478664	1.237019779	-0.000525382
O	-0.839843713	2.31703528	-0.001001251
N	3.975452157	-1.076929522	0.000498413
H	4.42269594	-0.170356322	0.000885229
C	4.831499927	-2.191547312	-0.001001464
O	6.04095195	-2.039842897	-0.001750663
C	4.117281497	-3.45372085	-0.001505858
H	4.683866046	-4.3687516	-0.002644493
C	2.767112521	-3.457538722	-0.000545762
H	2.190149284	-4.370503488	-0.000864439
N	2.029121111	-2.306657883	0.00090465
H	1.002784851	-2.328100682	0.001581719
C	2.601892049	-1.061992842	0.001455297
O	1.951571165	-0.020774537	0.00269169

17_Uracil-Uracil_BP_0.95

0	1		
N	-0.729999134	0.022767626	0.000914648
H	0.298422555	0.074004465	0.001623038
C	-1.296824528	-1.24042682	0.001502336
O	-0.594098857	-2.253517508	0.002633705
C	-2.743622291	-1.262331701	0.000479378
H	-3.249590454	-2.211835169	0.000833106
C	-3.422019974	-0.095909207	-0.00092259
H	-4.500897091	-0.049216027	-0.001745463
N	-2.774836844	1.105408947	-0.001418075
H	-3.283838068	1.973877391	-0.002485743
C	-1.391478664	1.237019779	-0.000525382
O	-0.839843713	2.31703528	-0.001001251

N	4.064139997	-1.081551127	0.000498868
H	4.51138378	-0.174977927	0.000885684
C	4.920187767	-2.196168917	-0.001001009
O	6.12963979	-2.044464502	-0.001750208
C	4.205969337	-3.458342455	-0.001505403
H	4.772553886	-4.373373205	-0.002644038
C	2.855800361	-3.462160327	-0.000545307
H	2.278837124	-4.375125093	-0.000863984
N	2.117808951	-2.311279488	0.000905105
H	1.091472691	-2.332722287	0.001582174
C	2.690579889	-1.066614447	0.001455752
O	2.040259005	-0.025396142	0.002692145

17_Uracil-Uracil_BP_1.00

0	1		
N	-0.729999134	0.022767626	0.000914648
H	0.298422555	0.074004465	0.001623038
C	-1.296824528	-1.24042682	0.001502336
O	-0.594098857	-2.253517508	0.002633705
C	-2.743622291	-1.262331701	0.000479378
H	-3.249590454	-2.211835169	0.000833106
C	-3.422019974	-0.095909207	-0.00092259
H	-4.500897091	-0.049216027	-0.001745463
N	-2.774836844	1.105408947	-0.001418075
H	-3.283838068	1.973877391	-0.002485743
C	-1.391478664	1.237019779	-0.000525382
O	-0.839843713	2.31703528	-0.001001251
N	4.152727849	-1.086167522	0.000499322
H	4.599971632	-0.179594322	0.000886138
C	5.008775619	-2.200785312	-0.001000555
O	6.218227642	-2.049080897	-0.001749754
C	4.294557189	-3.46295885	-0.001504949
H	4.861141738	-4.3779896	-0.002643584
C	2.944388213	-3.466776722	-0.000544853
H	2.367424976	-4.379741488	-0.00086353
N	2.206396803	-2.315895883	0.000905559
H	1.180060543	-2.337338682	0.001582628
C	2.779167741	-1.071230842	0.001456206
O	2.128846857	-0.030012537	0.002692599

17_Uracil-Uracil_BP_1.05

0	1		
N	-0.729999134	0.022767626	0.000914648

H	0.298422555	0.074004465	0.001623038
C	-1.296824528	-1.24042682	0.001502336
O	-0.594098857	-2.253517508	0.002633705
C	-2.743622291	-1.262331701	0.000479378
H	-3.249590454	-2.211835169	0.000833106
C	-3.422019974	-0.095909207	-0.00092259
H	-4.500897091	-0.049216027	-0.001745463
N	-2.774836844	1.105408947	-0.001418075
H	-3.283838068	1.973877391	-0.002485743
C	-1.391478664	1.237019779	-0.000525382
O	-0.839843713	2.31703528	-0.001001251
N	4.241515323	-1.090794319	0.000499777
H	4.688759106	-0.184221119	0.000886593
C	5.097563093	-2.205412109	-0.0010001
O	6.307015116	-2.053707694	-0.001749299
C	4.383344663	-3.467585647	-0.001504494
H	4.949929212	-4.382616397	-0.002643129
C	3.033175687	-3.471403519	-0.000544398
H	2.45621245	-4.384368285	-0.000863075
N	2.295184277	-2.32052268	0.000906014
H	1.268848017	-2.341965479	0.001583083
C	2.867955215	-1.075857639	0.001456661
O	2.217634331	-0.034639334	0.002693054

17_Uracil-Uracil_BP_1.10

0	1		
N	-0.729999134	0.022767626	0.000914648
H	0.298422555	0.074004465	0.001623038
C	-1.296824528	-1.24042682	0.001502336
O	-0.594098857	-2.253517508	0.002633705
C	-2.743622291	-1.262331701	0.000479378
H	-3.249590454	-2.211835169	0.000833106
C	-3.422019974	-0.095909207	-0.00092259
H	-4.500897091	-0.049216027	-0.001745463
N	-2.774836844	1.105408947	-0.001418075
H	-3.283838068	1.973877391	-0.002485743
C	-1.391478664	1.237019779	-0.000525382
O	-0.839843713	2.31703528	-0.001001251
N	4.330202842	-1.095415907	0.000500232
H	4.777446625	-0.188842707	0.000887048
C	5.186250612	-2.210033697	-0.000999645
O	6.395702635	-2.058329282	-0.001748844
C	4.472032182	-3.472207235	-0.001504039

H	5.038616731	-4.387237985	-0.002642674
C	3.121863206	-3.476025107	-0.000543943
H	2.544899969	-4.388989873	-0.00086262
N	2.383871796	-2.325144268	0.000906469
H	1.357535536	-2.346587067	0.001583538
C	2.956642734	-1.080479227	0.001457116
O	2.30632185	-0.039260922	0.002693509

17_Uracil-Uracil_BP_1.25

0	1		
N	-0.729999134	0.022767626	0.000914648
H	0.298422555	0.074004465	0.001623038
C	-1.296824528	-1.24042682	0.001502336
O	-0.594098857	-2.253517508	0.002633705
C	-2.743622291	-1.262331701	0.000479378
H	-3.249590454	-2.211835169	0.000833106
C	-3.422019974	-0.095909207	-0.00092259
H	-4.500897091	-0.049216027	-0.001745463
N	-2.774836844	1.105408947	-0.001418075
H	-3.283838068	1.973877391	-0.002485743
C	-1.391478664	1.237019779	-0.000525382
O	-0.839843713	2.31703528	-0.001001251
N	4.596091985	-1.109271635	0.000501596
H	5.043335768	-0.202698435	0.000888412
C	5.452139755	-2.223889425	-0.000998281
O	6.661591778	-2.07218501	-0.00174748
C	4.737921325	-3.486062963	-0.001502675
H	5.304505874	-4.401093713	-0.00264131
C	3.387752349	-3.489880835	-0.000542579
H	2.810789112	-4.402845601	-0.000861256
N	2.649760939	-2.338999996	0.000907833
H	1.623424679	-2.360442795	0.001584902
C	3.222531877	-1.094334955	0.00145848
O	2.572210993	-0.05311665	0.002694873

17_Uracil-Uracil_BP_A

0	1		
N	-0.729999134	0.022767626	0.000914648
H	0.298422555	0.074004465	0.001623038
C	-1.296824528	-1.24042682	0.001502336
O	-0.594098857	-2.253517508	0.002633705
C	-2.743622291	-1.262331701	0.000479378
H	-3.249590454	-2.211835169	0.000833106

C	-3.422019974	-0.095909207	-0.00092259
H	-4.500897091	-0.049216027	-0.001745463
N	-2.774836844	1.105408947	-0.001418075
H	-3.283838068	1.973877391	-0.002485743
C	-1.391478664	1.237019779	-0.000525382
O	-0.839843713	2.31703528	-0.001001251

17_Uracil-Uracil_BP_B

0	1		
N	3.975452157	-1.076929522	0.000498413
H	4.42269594	-0.170356322	0.000885229
C	4.831499927	-2.191547312	-0.001001464
O	6.04095195	-2.039842897	-0.001750663
C	4.117281497	-3.45372085	-0.001505858
H	4.683866046	-4.3687516	-0.002644493
C	2.767112521	-3.457538722	-0.000545762
H	2.190149284	-4.370503488	-0.000864439
N	2.029121111	-2.306657883	0.00090465
H	1.002784851	-2.328100682	0.001581719
C	2.601892049	-1.061992842	0.001455297
O	1.951571165	-0.020774537	0.00269169

20_AcOH-AcOH_0.90

0	1		
C	-1.061709204	1.297140572	0.292060003
O	-0.358161116	2.270458613	0.531812668
O	-0.589303516	0.094917758	0.003788813
H	0.404435659	0.127722621	0.018411838
C	-2.558427798	1.342549823	0.29625732
H	-2.895997978	2.347464002	0.51831634
H	-2.932889278	1.022390451	-0.672995551
H	-2.93721196	0.644910433	1.039557084
C	2.630513912	1.107716378	0.269968222
O	1.926895547	0.13440189	0.03018062
O	2.158087578	2.310151766	0.557746693
H	1.164299038	2.277253532	0.543346189
C	4.12722636	1.061813632	0.268003827
H	4.464805924	0.060494439	0.030478332
H	4.508724905	1.772166571	-0.461465071
H	4.498742671	1.364508149	1.244059188

20_AcOH-AcOH_0.95

0	1		
C	-1.061709204	1.297140572	0.292060003
O	-0.358161116	2.270458613	0.531812668
O	-0.589303516	0.094917758	0.003788813
H	0.404435659	0.127722621	0.018411838
C	-2.558427798	1.342549823	0.29625732
H	-2.895997978	2.347464002	0.51831634
H	-2.932889278	1.022390451	-0.672995551
H	-2.93721196	0.644910433	1.039557084
C	2.715089441	1.108090636	0.270615466
O	2.011471076	0.134776148	0.030827864
O	2.242663107	2.310526024	0.558393937
H	1.248874567	2.27762779	0.543993433
C	4.211801889	1.06218789	0.268651071
H	4.549381453	0.060868697	0.031125576
H	4.593300434	1.772540829	-0.460817827
H	4.5833182	1.364882407	1.244706432

20_AcOH-AcOH_1.00

0	1		
C	-1.061709204	1.297140572	0.292060003
O	-0.358161116	2.270458613	0.531812668
O	-0.589303516	0.094917758	0.003788813
H	0.404435659	0.127722621	0.018411838
C	-2.558427798	1.342549823	0.29625732
H	-2.895997978	2.347464002	0.51831634
H	-2.932889278	1.022390451	-0.672995551
H	-2.93721196	0.644910433	1.039557084
C	2.799564974	1.108464452	0.271261944
O	2.095946609	0.135149964	0.031474342
O	2.32713864	2.31089984	0.559040415
H	1.3333501	2.278001606	0.544639911
C	4.296277422	1.062561706	0.269297549
H	4.633856986	0.061242513	0.031772054
H	4.677775967	1.772914645	-0.460171349
H	4.667793733	1.365256223	1.24535291

20_AcOH-AcOH_1.05

0	1		
C	-1.061709204	1.297140572	0.292060003
O	-0.358161116	2.270458613	0.531812668
O	-0.589303516	0.094917758	0.003788813

H	0.404435659	0.127722621	0.018411838
C	-2.558427798	1.342549823	0.29625732
H	-2.895997978	2.347464002	0.51831634
H	-2.932889278	1.022390451	-0.672995551
H	-2.93721196	0.644910433	1.039557084
C	2.884240499	1.108839153	0.271909953
O	2.180622134	0.135524665	0.032122351
O	2.411814165	2.311274541	0.559688424
H	1.418025625	2.278376307	0.54528792
C	4.380952947	1.062936407	0.269945558
H	4.718532511	0.061617214	0.032420063
H	4.762451492	1.773289346	-0.45952334
H	4.752469258	1.365630924	1.246000919

20_AcOH-AcOH_1.10

0	1		
C	-1.061709204	1.297140572	0.292060003
O	-0.358161116	2.270458613	0.531812668
O	-0.589303516	0.094917758	0.003788813
H	0.404435659	0.127722621	0.018411838
C	-2.558427798	1.342549823	0.29625732
H	-2.895997978	2.347464002	0.51831634
H	-2.932889278	1.022390451	-0.672995551
H	-2.93721196	0.644910433	1.039557084
C	2.968816028	1.109213411	0.272557196
O	2.265197663	0.135898923	0.032769594
O	2.496389694	2.311648799	0.560335667
H	1.502601154	2.278750565	0.545935163
C	4.465528476	1.063310665	0.270592801
H	4.80310804	0.061991472	0.033067306
H	4.847027021	1.773663604	-0.458876097
H	4.837044787	1.366005182	1.246648162

20_AcOH-AcOH_1.25

0	1		
C	-1.061709204	1.297140572	0.292060003
O	-0.358161116	2.270458613	0.531812668
O	-0.589303516	0.094917758	0.003788813
H	0.404435659	0.127722621	0.018411838
C	-2.558427798	1.342549823	0.29625732
H	-2.895997978	2.347464002	0.51831634
H	-2.932889278	1.022390451	-0.672995551
H	-2.93721196	0.644910433	1.039557084

C	3.222542615	1.110336186	0.274498927
O	2.51892425	0.137021698	0.034711325
O	2.750116281	2.312771574	0.562277398
H	1.756327741	2.27987334	0.547876894
C	4.719255063	1.06443344	0.272534532
H	5.056834627	0.063114247	0.035009037
H	5.100753608	1.774786379	-0.456934366
H	5.090771374	1.367127957	1.248589893

20_AcOH-AcOH_A

0	1		
C	-1.061709204	1.297140572	0.292060003
O	-0.358161116	2.270458613	0.531812668
O	-0.589303516	0.094917758	0.003788813
H	0.404435659	0.127722621	0.018411838
C	-2.558427798	1.342549823	0.29625732
H	-2.895997978	2.347464002	0.51831634
H	-2.932889278	1.022390451	-0.672995551
H	-2.93721196	0.644910433	1.039557084

20_AcOH-AcOH_B

0	1		
C	2.630513912	1.107716378	0.269968222
O	1.926895547	0.13440189	0.03018062
O	2.158087578	2.310151766	0.557746693
H	1.164299038	2.277253532	0.543346189
C	4.12722636	1.061813632	0.268003827
H	4.464805924	0.060494439	0.030478332
H	4.508724905	1.772166571	-0.461465071
H	4.498742671	1.364508149	1.244059188

28_Benzene-Uracil_pi-pi_0.90

0	1		
C	0.825769105	1.236524837	-0.040250437
H	1.521013168	2.063125196	-0.082471448
C	1.300159924	-0.062940879	0.127256013
H	2.363657528	-0.242261131	0.207674197
C	0.403523117	-1.128552182	0.198244857
H	0.773753376	-2.137426768	0.324121091
C	-0.967809494	-0.895190489	0.103139941
H	-1.665209	-1.719983422	0.160427446
C	-1.443508379	0.404483279	-0.062441302
H	-2.507511245	0.585501124	-0.124150163

C	-0.545755493	1.468768748	-0.136247408
H	-0.914221904	2.477422201	-0.267855164
N	-0.287522703	0.707122355	2.884899047
H	-0.660830096	1.608883785	2.622006076
C	1.104623979	0.634135449	3.016912386
O	1.795534298	1.628559141	2.872078321
C	1.583524093	-0.69994226	3.335022588
H	2.640576187	-0.852158199	3.469143724
C	0.703814866	-1.714323436	3.461239113
H	1.009742387	-2.722744045	3.697763474
N	-0.641431021	-1.529291516	3.29114697
H	-1.290178852	-2.291850459	3.390016141
C	-1.21587933	-0.304490488	2.991732354
O	-2.413667742	-0.153667213	2.849620162

28_Benzene-Uracil_pi-pi_0.95

0	1		
C	0.825769105	1.236524837	-0.040250437
H	1.521013168	2.063125196	-0.082471448
C	1.300159924	-0.062940879	0.127256013
H	2.363657528	-0.242261131	0.207674197
C	0.403523117	-1.128552182	0.198244857
H	0.773753376	-2.137426768	0.324121091
C	-0.967809494	-0.895190489	0.103139941
H	-1.665209	-1.719983422	0.160427446
C	-1.443508379	0.404483279	-0.062441302
H	-2.507511245	0.585501124	-0.124150163
C	-0.545755493	1.468768748	-0.136247408
H	-0.914221904	2.477422201	-0.267855164
N	-0.281657184	0.690635265	3.03974698
H	-0.654964577	1.592396695	2.776854009
C	1.110489498	0.617648359	3.171760319
O	1.801399817	1.612072051	3.026926254
C	1.589389612	-0.71642935	3.489870521
H	2.646441706	-0.868645289	3.623991657
C	0.709680385	-1.730810526	3.616087046
H	1.015607906	-2.739231135	3.852611407
N	-0.635565502	-1.545778606	3.445994903
H	-1.284313333	-2.308337549	3.544864074
C	-1.210013811	-0.320977578	3.146580287
O	-2.407802223	-0.170154303	3.004468095

28_Benzene-Uracil_pi-pi_1.00

0	1		
C	0.825769105	1.236524837	-0.040250437
H	1.521013168	2.063125196	-0.082471448
C	1.300159924	-0.062940879	0.127256013
H	2.363657528	-0.242261131	0.207674197
C	0.403523117	-1.128552182	0.198244857
H	0.773753376	-2.137426768	0.324121091
C	-0.967809494	-0.895190489	0.103139941
H	-1.665209	-1.719983422	0.160427446
C	-1.443508379	0.404483279	-0.062441302
H	-2.507511245	0.585501124	-0.124150163
C	-0.545755493	1.468768748	-0.136247408
H	-0.914221904	2.477422201	-0.267855164
N	-0.275804152	0.674183275	3.194265248
H	-0.649111545	1.575944705	2.931372277
C	1.11634253	0.601196369	3.326278587
O	1.807252849	1.595620061	3.181444522
C	1.595242644	-0.73288134	3.644388789
H	2.652294738	-0.885097279	3.778509925
C	0.715533417	-1.747262516	3.770605314
H	1.021460938	-2.755683125	4.007129675
N	-0.62971247	-1.562230596	3.600513171
H	-1.278460301	-2.324789539	3.699382342
C	-1.204160779	-0.337429568	3.301098555
O	-2.401949191	-0.186606293	3.158986363

28_Benzene-Uracil_pi-pi_1.05

0	1		
C	0.825769105	1.236524837	-0.040250437
H	1.521013168	2.063125196	-0.082471448
C	1.300159924	-0.062940879	0.127256013
H	2.363657528	-0.242261131	0.207674197
C	0.403523117	-1.128552182	0.198244857
H	0.773753376	-2.137426768	0.324121091
C	-0.967809494	-0.895190489	0.103139941
H	-1.665209	-1.719983422	0.160427446
C	-1.443508379	0.404483279	-0.062441302
H	-2.507511245	0.585501124	-0.124150163
C	-0.545755493	1.468768748	-0.136247408
H	-0.914221904	2.477422201	-0.267855164
N	-0.269951039	0.657731056	3.348785671
H	-0.643258432	1.559492486	3.0858927

C	1.122195643	0.58474415	3.48079901
O	1.813105962	1.579167842	3.335964945
C	1.601095757	-0.749333559	3.798909212
H	2.658147851	-0.901549498	3.933030348
C	0.72138653	-1.763714735	3.925125737
H	1.027314051	-2.772135344	4.161650098
N	-0.623859357	-1.578682815	3.755033594
H	-1.272607188	-2.341241758	3.853902765
C	-1.198307666	-0.353881787	3.455618978
O	-2.396096078	-0.203058512	3.313506786

28_Benzene-Uracil_pi-pi_1.10

0	1		
C	0.825769105	1.236524837	-0.040250437
H	1.521013168	2.063125196	-0.082471448
C	1.300159924	-0.062940879	0.127256013
H	2.363657528	-0.242261131	0.207674197
C	0.403523117	-1.128552182	0.198244857
H	0.773753376	-2.137426768	0.324121091
C	-0.967809494	-0.895190489	0.103139941
H	-1.665209	-1.719983422	0.160427446
C	-1.443508379	0.404483279	-0.062441302
H	-2.507511245	0.585501124	-0.124150163
C	-0.545755493	1.468768748	-0.136247408
H	-0.914221904	2.477422201	-0.267855164
N	-0.264108097	0.641307428	3.503037557
H	-0.63741549	1.543068858	3.240144586
C	1.128038585	0.568320522	3.635050896
O	1.818948904	1.562744214	3.490216831
C	1.606938699	-0.765757187	3.953161098
H	2.663990793	-0.917973126	4.087282234
C	0.727229472	-1.780138363	4.079377623
H	1.033156993	-2.788558972	4.315901984
N	-0.618016415	-1.595106443	3.90928548
H	-1.266764246	-2.357665386	4.008154651
C	-1.192464724	-0.370305415	3.609870864
O	-2.390253136	-0.21948214	3.467758672

28_Benzene-Uracil_pi-pi_1.25

0	1		
C	0.825769105	1.236524837	-0.040250437
H	1.521013168	2.063125196	-0.082471448
C	1.300159924	-0.062940879	0.127256013

H	2.363657528	-0.242261131	0.207674197
C	0.403523117	-1.128552182	0.198244857
H	0.773753376	-2.137426768	0.324121091
C	-0.967809494	-0.895190489	0.103139941
H	-1.665209	-1.719983422	0.160427446
C	-1.443508379	0.404483279	-0.062441302
H	-2.507511245	0.585501124	-0.124150163
C	-0.545755493	1.468768748	-0.136247408
H	-0.914221904	2.477422201	-0.267855164
N	-0.24660989	0.592122605	3.964984948
H	-0.619917283	1.493884035	3.702091977
C	1.145536792	0.519135699	4.096998287
O	1.836447111	1.513559391	3.952164222
C	1.624436906	-0.81494201	4.415108489
H	2.681489	-0.967157949	4.549229625
C	0.744727679	-1.829323186	4.541325014
H	1.0506552	-2.837743795	4.777849375
N	-0.600518208	-1.644291266	4.371232871
H	-1.249266039	-2.406850209	4.470102042
C	-1.174966517	-0.419490238	4.071818255
O	-2.372754929	-0.268666963	3.929706063

28_Benzene-Uracil_pi-pi_A

0	1		
C	0.825769105	1.236524837	-0.040250437
H	1.521013168	2.063125196	-0.082471448
C	1.300159924	-0.062940879	0.127256013
H	2.363657528	-0.242261131	0.207674197
C	0.403523117	-1.128552182	0.198244857
H	0.773753376	-2.137426768	0.324121091
C	-0.967809494	-0.895190489	0.103139941
H	-1.665209	-1.719983422	0.160427446
C	-1.443508379	0.404483279	-0.062441302
H	-2.507511245	0.585501124	-0.124150163
C	-0.545755493	1.468768748	-0.136247408
H	-0.914221904	2.477422201	-0.267855164

28_Benzene-Uracil_pi-pi_B

0	1		
N	-0.287522703	0.707122355	2.884899047
H	-0.660830096	1.608883785	2.622006076
C	1.104623979	0.634135449	3.016912386
O	1.795534298	1.628559141	2.872078321

C	1.583524093	-0.69994226	3.335022588
H	2.640576187	-0.852158199	3.469143724
C	0.703814866	-1.714323436	3.461239113
H	1.009742387	-2.722744045	3.697763474
N	-0.641431021	-1.529291516	3.29114697
H	-1.290178852	-2.291850459	3.390016141
C	-1.21587933	-0.304490488	2.991732354
O	-2.413667742	-0.153667213	2.849620162

29_Pyridine-Uracil_pi-pi_0.90

0	1		
N	1.210755335	0.028675778	0.329711113
C	0.61193497	-1.158449011	0.153451764
H	1.251477905	-2.029523404	0.219292947
C	-0.75131399	-1.308649559	-0.088834071
H	-1.170415774	-2.296869318	-0.213383204
C	-1.54786767	-0.169940268	-0.156466912
H	-2.611012747	-0.245954694	-0.338755744
C	-0.943622374	1.070636117	0.019823098
H	-1.518814305	1.984500285	-0.011644028
C	0.42771857	1.116108629	0.257348792
H	0.924694513	2.068051734	0.397547985
N	-0.746112695	-0.302816882	2.92822204
H	-1.641001714	-0.734680373	2.743618552
C	-0.745857809	1.09499291	3.021233041
O	-1.785740886	1.723192713	2.906382911
C	0.573636944	1.654074255	3.248796108
H	0.67495331	2.721296425	3.344664289
C	1.643510535	0.835381953	3.320316161
H	2.647389575	1.204046651	3.468742694
N	1.525449394	-0.521911311	3.207761497
H	2.338885388	-1.114098661	3.199593858
C	0.325002457	-1.169143739	2.9813798
O	0.23286521	-2.374561815	2.847800521

29_Pyridine-Uracil_pi-pi_0.95

0	1		
N	1.210755335	0.028675778	0.329711113
C	0.61193497	-1.158449011	0.153451764
H	1.251477905	-2.029523404	0.219292947
C	-0.75131399	-1.308649559	-0.088834071
H	-1.170415774	-2.296869318	-0.213383204
C	-1.54786767	-0.169940268	-0.156466912

H	-2.611012747	-0.245954694	-0.338755744
C	-0.943622374	1.070636117	0.019823098
H	-1.518814305	1.984500285	-0.011644028
C	0.42771857	1.116108629	0.257348792
H	0.924694513	2.068051734	0.397547985
N	-0.73036703	-0.293799387	3.104724539
H	-1.625256049	-0.725662878	2.920121051
C	-0.730112144	1.104010405	3.19773554
O	-1.769995221	1.732210208	3.08288541
C	0.589382609	1.66309175	3.425298607
H	0.690698975	2.73031392	3.521166788
C	1.6592562	0.844399448	3.49681866
H	2.66313524	1.213064146	3.645245193
N	1.541195059	-0.512893816	3.384263996
H	2.354631053	-1.105081166	3.376096357
C	0.340748122	-1.160126244	3.157882299
O	0.248610875	-2.36554432	3.02430302

29_Pyridine-Uracil_pi-pi_1.00

0	1		
N	1.210755335	0.028675778	0.329711113
C	0.61193497	-1.158449011	0.153451764
H	1.251477905	-2.029523404	0.219292947
C	-0.75131399	-1.308649559	-0.088834071
H	-1.170415774	-2.296869318	-0.213383204
C	-1.54786767	-0.169940268	-0.156466912
H	-2.611012747	-0.245954694	-0.338755744
C	-0.943622374	1.070636117	0.019823098
H	-1.518814305	1.984500285	-0.011644028
C	0.42771857	1.116108629	0.257348792
H	0.924694513	2.068051734	0.397547985
N	-0.714834682	-0.284904059	3.278835825
H	-1.609723701	-0.71676755	3.094232337
C	-0.714579796	1.112905733	3.371846826
O	-1.754462873	1.741105536	3.256996696
C	0.604914957	1.671987078	3.599409893
H	0.706231323	2.739209248	3.695278074
C	1.674788548	0.853294776	3.670929946
H	2.678667588	1.221959474	3.819356479
N	1.556727407	-0.503998488	3.558375282
H	2.370163401	-1.096185838	3.550207643
C	0.35628047	-1.151230916	3.331993585
O	0.264143223	-2.356648992	3.198414306

29_Pyridine-Uracil_pi-pi_1.05

0	1		
N	1.210755335	0.028675778	0.329711113
C	0.61193497	-1.158449011	0.153451764
H	1.251477905	-2.029523404	0.219292947
C	-0.75131399	-1.308649559	-0.088834071
H	-1.170415774	-2.296869318	-0.213383204
C	-1.54786767	-0.169940268	-0.156466912
H	-2.611012747	-0.245954694	-0.338755744
C	-0.943622374	1.070636117	0.019823098
H	-1.518814305	1.984500285	-0.011644028
C	0.42771857	1.116108629	0.257348792
H	0.924694513	2.068051734	0.397547985
N	-0.699453608	-0.276095365	3.451251401
H	-1.594342627	-0.707958856	3.266647913
C	-0.699198722	1.121714427	3.544262402
O	-1.739081799	1.74991423	3.429412272
C	0.620296031	1.680795772	3.771825469
H	0.721612397	2.748017942	3.86769365
C	1.690169622	0.86210347	3.843345522
H	2.694048662	1.230768168	3.991772055
N	1.572108481	-0.495189794	3.730790858
H	2.385544475	-1.087377144	3.722623219
C	0.371661544	-1.142422222	3.504409161
O	0.279524297	-2.347840298	3.370829882

29_Pyridine-Uracil_pi-pi_1.10

0	1		
N	1.210755335	0.028675778	0.329711113
C	0.61193497	-1.158449011	0.153451764
H	1.251477905	-2.029523404	0.219292947
C	-0.75131399	-1.308649559	-0.088834071
H	-1.170415774	-2.296869318	-0.213383204
C	-1.54786767	-0.169940268	-0.156466912
H	-2.611012747	-0.245954694	-0.338755744
C	-0.943622374	1.070636117	0.019823098
H	-1.518814305	1.984500285	-0.011644028
C	0.42771857	1.116108629	0.257348792
H	0.924694513	2.068051734	0.397547985
N	-0.684223014	-0.26737285	3.621980149
H	-1.579112033	-0.699236341	3.437376661
C	-0.683968128	1.130436942	3.71499115

O	-1.723851205	1.758636745	3.60014102
C	0.635526625	1.689518287	3.942554217
H	0.736842991	2.756740457	4.038422398
C	1.705400216	0.870825985	4.01407427
H	2.709279256	1.239490683	4.162500803
N	1.587339075	-0.486467279	3.901519606
H	2.400775069	-1.078654629	3.893351967
C	0.386892138	-1.133699707	3.675137909
O	0.294754891	-2.339117783	3.54155863

29_Pyridine-Uracil_pi-pi_1.25

0	1		
N	1.210755335	0.028675778	0.329711113
C	0.61193497	-1.158449011	0.153451764
H	1.251477905	-2.029523404	0.219292947
C	-0.75131399	-1.308649559	-0.088834071
H	-1.170415774	-2.296869318	-0.213383204
C	-1.54786767	-0.169940268	-0.156466912
H	-2.611012747	-0.245954694	-0.338755744
C	-0.943622374	1.070636117	0.019823098
H	-1.518814305	1.984500285	-0.011644028
C	0.42771857	1.116108629	0.257348792
H	0.924694513	2.068051734	0.397547985
N	-0.639185288	-0.241579883	4.126834707
H	-1.534074307	-0.673443374	3.942231219
C	-0.638930402	1.156229909	4.219845708
O	-1.678813479	1.784429712	4.104995578
C	0.680564351	1.715311254	4.447408775
H	0.781880717	2.782533424	4.543276956
C	1.750437942	0.896618952	4.518928828
H	2.754316982	1.26528365	4.667355361
N	1.632376801	-0.460674312	4.406374164
H	2.445812795	-1.052861662	4.398206525
C	0.431929864	-1.10790674	4.179992467
O	0.339792617	-2.313324816	4.046413188

29_Pyridine-Uracil_pi-pi_A

0	1		
N	1.210755335	0.028675778	0.329711113
C	0.61193497	-1.158449011	0.153451764
H	1.251477905	-2.029523404	0.219292947
C	-0.75131399	-1.308649559	-0.088834071
H	-1.170415774	-2.296869318	-0.213383204

C	-1.54786767	-0.169940268	-0.156466912
H	-2.611012747	-0.245954694	-0.338755744
C	-0.943622374	1.070636117	0.019823098
H	-1.518814305	1.984500285	-0.011644028
C	0.42771857	1.116108629	0.257348792
H	0.924694513	2.068051734	0.397547985

29_Pyridine-Uracil_pi-pi_B

0	1		
N	-0.746112695	-0.302816882	2.92822204
H	-1.641001714	-0.734680373	2.743618552
C	-0.745857809	1.09499291	3.021233041
O	-1.785740886	1.723192713	2.906382911
C	0.573636944	1.654074255	3.248796108
H	0.67495331	2.721296425	3.344664289
C	1.643510535	0.835381953	3.320316161
H	2.647389575	1.204046651	3.468742694
N	1.525449394	-0.521911311	3.207761497
H	2.338885388	-1.114098661	3.199593858
C	0.325002457	-1.169143739	2.9813798
O	0.23286521	-2.374561815	2.847800521

47_Benzene-Benzene_TS_0.90

0	1		
C	0.729188666	1.113101217	0.326728253
H	1.303215897	2.014222336	0.15916027
C	1.375087369	-0.119366352	0.412776946
H	2.450514736	-0.174623998	0.313307203
C	0.635039807	-1.280553386	0.629385409
H	1.136334479	-2.236017467	0.700217157
C	-0.750985629	-1.209654296	0.757890338
H	-1.324525898	-2.111412827	0.924198912
C	-1.39703443	0.022670814	0.673089633
H	-2.472425369	0.07848826	0.773997991
C	-0.656897314	1.184296216	0.458338585
H	-1.157828445	2.140587131	0.395096082
C	0.115547135	0.090530573	3.721571936
H	0.237673542	0.316014029	2.671145502
C	-0.975534433	-0.664368038	4.148908205
H	-1.696038962	-1.020882295	3.425213784
C	-1.136234414	-0.958493361	5.501858272
H	-1.983342001	-1.544461925	5.832105813
C	-0.204351853	-0.497439981	6.43035235

H	-0.328245349	-0.725406142	7.480359849
C	0.887233246	0.257662073	6.005112066
H	1.610352331	0.615495253	6.72549472
C	1.04603714	0.551147092	4.651620749
H	1.893295058	1.137221887	4.321573424

47_Benzene-Benzene_TS_0.95

0	1		
C	0.729188666	1.113101217	0.326728253
H	1.303215897	2.014222336	0.15916027
C	1.375087369	-0.119366352	0.412776946
H	2.450514736	-0.174623998	0.313307203
C	0.635039807	-1.280553386	0.629385409
H	1.136334479	-2.236017467	0.700217157
C	-0.750985629	-1.209654296	0.757890338
H	-1.324525898	-2.111412827	0.924198912
C	-1.39703443	0.022670814	0.673089633
H	-2.472425369	0.07848826	0.773997991
C	-0.656897314	1.184296216	0.458338585
H	-1.157828445	2.140587131	0.395096082
C	0.134572645	0.11840774	3.884432701
H	0.256699052	0.343891196	2.834006267
C	-0.956508923	-0.636490871	4.31176897
H	-1.677013452	-0.993005128	3.588074549
C	-1.117208904	-0.930616194	5.664719037
H	-1.964316491	-1.516584758	5.994966578
C	-0.185326343	-0.469562814	6.593213115
H	-0.309219839	-0.697528975	7.643220614
C	0.906258756	0.28553924	6.167972831
H	1.629377841	0.64337242	6.888355485
C	1.06506265	0.579024259	4.814481514
H	1.912320568	1.165099054	4.484434189

47_Benzene-Benzene_TS_1.00

0	1		
C	0.729188666	1.113101217	0.326728253
H	1.303215897	2.014222336	0.15916027
C	1.375087369	-0.119366352	0.412776946
H	2.450514736	-0.174623998	0.313307203
C	0.635039807	-1.280553386	0.629385409
H	1.136334479	-2.236017467	0.700217157
C	-0.750985629	-1.209654296	0.757890338
H	-1.324525898	-2.111412827	0.924198912

C	-1.39703443	0.022670814	0.673089633
H	-2.472425369	0.07848826	0.773997991
C	-0.656897314	1.184296216	0.458338585
H	-1.157828445	2.140587131	0.395096082
C	0.153204042	0.145707432	4.043919807
H	0.275330449	0.371190888	2.993493373
C	-0.937877526	-0.609191179	4.471256076
H	-1.658382055	-0.965705436	3.747561655
C	-1.098577507	-0.903316502	5.824206143
H	-1.945685094	-1.489285066	6.154453684
C	-0.166694946	-0.442263122	6.752700221
H	-0.290588442	-0.670229283	7.80270772
C	0.924890153	0.312838932	6.327459937
H	1.648009238	0.670672112	7.047842591
C	1.083694047	0.606323951	4.97396862
H	1.930951965	1.192398746	4.643921295

47_Benzene-Benzene_TS_1.05

0	1		
C	0.729188666	1.113101217	0.326728253
H	1.303215897	2.014222336	0.15916027
C	1.375087369	-0.119366352	0.412776946
H	2.450514736	-0.174623998	0.313307203
C	0.635039807	-1.280553386	0.629385409
H	1.136334479	-2.236017467	0.700217157
C	-0.750985629	-1.209654296	0.757890338
H	-1.324525898	-2.111412827	0.924198912
C	-1.39703443	0.022670814	0.673089633
H	-2.472425369	0.07848826	0.773997991
C	-0.656897314	1.184296216	0.458338585
H	-1.157828445	2.140587131	0.395096082
C	0.17155475	0.172595843	4.201004177
H	0.293681157	0.398079299	3.150577743
C	-0.919526818	-0.582302768	4.628340446
H	-1.640031347	-0.938817025	3.904646025
C	-1.080226799	-0.876428091	5.981290513
H	-1.927334386	-1.462396655	6.311538054
C	-0.148344238	-0.415374711	6.909784591
H	-0.272237734	-0.643340872	7.95979209
C	0.943240861	0.339727343	6.484544307
H	1.666359946	0.697560523	7.204926961
C	1.102044755	0.633212362	5.13105299
H	1.949302673	1.219287157	4.801005665

47_Benzene-Benzene_TS_1.10

0	1		
C	0.729188666	1.113101217	0.326728253
H	1.303215897	2.014222336	0.15916027
C	1.375087369	-0.119366352	0.412776946
H	2.450514736	-0.174623998	0.313307203
C	0.635039807	-1.280553386	0.629385409
H	1.136334479	-2.236017467	0.700217157
C	-0.750985629	-1.209654296	0.757890338
H	-1.324525898	-2.111412827	0.924198912
C	-1.39703443	0.022670814	0.673089633
H	-2.472425369	0.07848826	0.773997991
C	-0.656897314	1.184296216	0.458338585
H	-1.157828445	2.140587131	0.395096082
C	0.189643482	0.199100394	4.355846002
H	0.311769889	0.42458385	3.305419568
C	-0.901438086	-0.555798217	4.783182271
H	-1.621942615	-0.912312474	4.05948785
C	-1.062138067	-0.84992354	6.136132338
H	-1.909245654	-1.435892104	6.466379879
C	-0.130255506	-0.38887016	7.064626416
H	-0.254149002	-0.616836321	8.114633915
C	0.961329593	0.366231894	6.639386132
H	1.684448678	0.724065074	7.359768786
C	1.120133487	0.659716913	5.285894815
H	1.967391405	1.245791708	4.95584749

47_Benzene-Benzene_TS_1.25

0	1		
C	0.729188666	1.113101217	0.326728253
H	1.303215897	2.014222336	0.15916027
C	1.375087369	-0.119366352	0.412776946
H	2.450514736	-0.174623998	0.313307203
C	0.635039807	-1.280553386	0.629385409
H	1.136334479	-2.236017467	0.700217157
C	-0.750985629	-1.209654296	0.757890338
H	-1.324525898	-2.111412827	0.924198912
C	-1.39703443	0.022670814	0.673089633
H	-2.472425369	0.07848826	0.773997991
C	-0.656897314	1.184296216	0.458338585
H	-1.157828445	2.140587131	0.395096082
C	0.24277343	0.276949158	4.810645059

H	0.364899837	0.502432614	3.760218625
C	-0.848308138	-0.477949453	5.237981328
H	-1.568812667	-0.83446371	4.514286907
C	-1.009008119	-0.772074776	6.590931395
H	-1.856115706	-1.35804334	6.921178936
C	-0.077125558	-0.311021396	7.519425473
H	-0.201019054	-0.538987557	8.569432972
C	1.014459541	0.444080658	7.094185189
H	1.737578626	0.801913838	7.814567843
C	1.173263435	0.737565677	5.740693872
H	2.020521353	1.323640472	5.410646547

47_Benzene-Benzene_TS_A

0	1		
C	0.729188666	1.113101217	0.326728253
H	1.303215897	2.014222336	0.15916027
C	1.375087369	-0.119366352	0.412776946
H	2.450514736	-0.174623998	0.313307203
C	0.635039807	-1.280553386	0.629385409
H	1.136334479	-2.236017467	0.700217157
C	-0.750985629	-1.209654296	0.757890338
H	-1.324525898	-2.111412827	0.924198912
C	-1.39703443	0.022670814	0.673089633
H	-2.472425369	0.07848826	0.773997991
C	-0.656897314	1.184296216	0.458338585
H	-1.157828445	2.140587131	0.395096082

47_Benzene-Benzene_TS_B

0	1		
C	0.115547135	0.090530573	3.721571936
H	0.237673542	0.316014029	2.671145502
C	-0.975534433	-0.664368038	4.148908205
H	-1.696038962	-1.020882295	3.425213784
C	-1.136234414	-0.958493361	5.501858272
H	-1.983342001	-1.544461925	5.832105813
C	-0.204351853	-0.497439981	6.43035235
H	-0.328245349	-0.725406142	7.480359849
C	0.887233246	0.257662073	6.005112066
H	1.610352331	0.615495253	6.72549472
C	1.04603714	0.551147092	4.651620749
H	1.893295058	1.137221887	4.321573424

SBG31

AlAs			
0	1		
Al	0.00000	0.00000	0.00000
As	1.41520	1.41520	1.41520
-2	0.00000	2.83050	2.83050
-2	2.83050	0.00000	2.83050
-2	2.83050	2.83050	0.00000
AlP			
0	1		
Al	0.00000	0.00000	0.00000
P	1.36580	1.36580	1.36580
-2	0.00000	2.73150	2.73150
-2	2.73150	0.00000	2.73150
-2	2.73150	2.73150	0.00000
AlSb			
0	1		
Al	0.00000	0.00000	0.00000
Sb	1.53400	1.53400	1.53400
-2	0.00000	3.06800	3.06800
-2	3.06800	0.00000	3.06800
-2	3.06800	3.06800	0.00000
BaS			
0	1		
Ba	0.00000	0.00000	0.00000
S	3.19500	3.19500	3.19500
-2	0.00000	3.19500	3.19500
-2	3.19500	0.00000	3.19500
-2	3.19500	3.19500	0.00000
B-As			
0	1		
B	0.00000	0.00000	0.00000
As	1.19430	1.19430	1.19430
-2	0.00000	2.38850	2.38850
-2	2.38850	0.00000	2.38850
-2	2.38850	2.38850	0.00000

BaSe			
0	1		
Ba	0.00000	0.00000	0.00000
Se	3.29700	3.29700	3.29700
-2	0.00000	3.29700	3.29700
-2	3.29700	0.00000	3.29700
-2	3.29700	3.29700	0.00000
BaTe			
0	1		
Ba	0.00000	0.00000	0.00000
Te	3.50300	3.50300	3.50300
-2	0.00000	3.50300	3.50300
-2	3.50300	0.00000	3.50300
-2	3.50300	3.50300	0.00000
BP			
0	1		
B	0.00000	0.00000	0.00000
P	1.13730	1.13730	1.13730
-2	0.00000	2.27450	2.27450
-2	2.27450	0.00000	2.27450
-2	2.27450	2.27450	0.00000
C			
0	1		
C	0.00000	0.00000	0.00000
C	0.88750	0.88750	0.88750
-2	0.00000	1.77500	1.77500
-2	1.77500	0.00000	1.77500
-2	1.77500	1.77500	0.00000
CdS			
0	1		
Cd	0.00000	0.00000	0.00000
S	1.45450	1.45450	1.45450
-2	0.00000	2.90900	2.90900
-2	2.90900	0.00000	2.90900
-2	2.90900	2.90900	0.00000
CdSe			
0	1		
Cd	0.00000	0.00000	0.00000

Se	1.51300	1.51300	1.51300
-2	0.00000	3.02600	3.02600
-2	3.02600	0.00000	3.02600
-2	3.02600	3.02600	0.00000
CdTe			
0	1		
Cd	0.00000	0.00000	0.00000
Te	1.62000	1.62000	1.62000
-2	0.00000	3.24000	3.24000
-2	3.24000	0.00000	3.24000
-2	3.24000	3.24000	0.00000
GaAs			
0	1		
Ga	0.00000	0.00000	0.00000
As	1.43150	1.43150	1.43150
-2	0.00000	2.86300	2.86300
-2	2.86300	0.00000	2.86300
-2	2.86300	2.86300	0.00000
GaN			
0	1		
Ga	0.00000	0.00000	0.00000
N	1.12450	1.12450	1.12450
-2	0.00000	2.24900	2.24900
-2	2.24900	0.00000	2.24900
-2	2.24900	2.24900	0.00000
GaNwu			
0	1		
Ga	1.58000	0.91220	0.00000
Ga	1.58000	-0.91220	2.56250
N	1.58000	0.91220	1.92190
N	1.58000	-0.91220	4.48440
-2	1.58000	-2.73660	0.00000
-2	1.58000	2.73660	0.00000
-2	0.00000	0.00000	5.12500

GaP			
0	1		
Ga	0.00000	0.00000	0.00000
P	1.36380	1.36380	1.36380
-2	0.00000	2.72750	2.72750
-2	2.72750	0.00000	2.72750
-2	2.72750	2.72750	0.00000
GaSb			
0	1		
Ga	0.00000	0.00000	0.00000
Sb	1.52400	1.52400	1.52400
-2	0.00000	3.04800	3.04800
-2	3.04800	0.00000	3.04800
-2	3.04800	3.04800	0.00000
Ge			
0	1		
Ge	0.00000	0.00000	0.00000
Ge	1.41250	1.41250	1.41250
-2	0.00000	2.82500	2.82500
-2	2.82500	0.00000	2.82500
-2	2.82500	2.82500	0.00000
InAs			
0	1		
In	0.00000	0.00000	0.00000
As	1.51450	1.51450	1.51450
-2	0.00000	3.02900	3.02900
-2	3.02900	0.00000	3.02900
-2	3.02900	3.02900	0.00000
InN			
0	1		
In	1.76850	1.02100	0.00000
In	1.76850	-1.02100	2.85200
N	1.76850	1.02100	2.13900
N	1.76850	-1.02100	4.99100
-2	1.76850	-3.06310	0.00000
-2	1.76850	3.06310	0.00000
-2	0.00000	0.00000	5.70400

InP			
0	1		
In	0.00000	0.00000	0.00000
P	1.46720	1.46720	1.46720
-2	0.00000	2.93450	2.93450
-2	2.93450	0.00000	2.93450
-2	2.93450	2.93450	0.00000
InSb			
0	1		
In	0.00000	0.00000	0.00000
Sb	1.61980	1.61980	1.61980
-2	0.00000	3.23950	3.23950
-2	3.23950	0.00000	3.23950
-2	3.23950	3.23950	0.00000
MgS			
0	1		
Mg	0.00000	0.00000	0.00000
S	1.40550	1.40550	1.40550
-2	0.00000	2.81100	2.81100
-2	2.81100	0.00000	2.81100
-2	2.81100	2.81100	0.00000
MgSe			
0	1		
Mg	0.00000	0.00000	0.00000
Se	2.70000	2.70000	2.70000
-2	0.00000	2.70000	2.70000
-2	2.70000	0.00000	2.70000
-2	2.70000	2.70000	0.00000
MgTe			
0	1		
Mg	0.00000	0.00000	0.00000
Te	1.61130	1.61130	1.61130
-2	0.00000	3.22250	3.22250
-2	3.22250	0.00000	3.22250
-2	3.22250	3.22250	0.00000
SiC			
0	1		
Si	0.00000	0.00000	0.00000

C	1.09250	1.09250	1.09250
-2	0.00000	2.18500	2.18500
-2	2.18500	0.00000	2.18500
-2	2.18500	2.18500	0.00000
Si			
0	1		
Si	0.00000	0.00000	0.00000
Si	1.35750	1.35750	1.35750
-2	0.00000	2.71500	2.71500
-2	2.71500	0.00000	2.71500
-2	2.71500	2.71500	0.00000
ZnO			
0	1		
Zn	-0.82273	0.47197	-2.33109
Zn	0.82071	-0.47267	0.32441
O	-0.82068	0.48610	-0.32441
O	0.82270	-0.48540	2.33109
-2	3.29094	-0.01613	-0.00320
-2	-1.631387	2.850389	0.00054
-2	0.005344	0.001885	5.313519
ZnS			
0,1			
Zn	0.00000	0.00000	0.00000
S	1.35220	1.35220	1.35220
-2	0.00000	2.70450	2.70450
-2	2.70450	0.00000	2.70450
-2	2.70450	2.70450	0.00000
ZnSe			
0,1			
Zn	0.00000	0.00000	0.00000
Se	1.41700	1.41700	1.41700
-2	0.00000	2.83400	2.83400
-2	2.83400	0.00000	2.83400
-2	2.83400	2.83400	0.00000
ZnTe			
0,1			
Zn	0.00000	0.00000	0.00000
Te	1.52230	1.52230	1.52230

-2	0.00000	3.04450	3.04450
-2	3.04450	0.00000	3.04450
-2	3.04450	3.04450	0.00000

EE69

acetaldehyde			
0	1		
O	1.212008	0.374458	0.000000
C	0.000000	0.462805	0.000000
H	-0.486928	1.460337	0.000000
C	-0.941279	-0.711815	0.000000
H	-0.384684	-1.649523	0.000000
H	-1.588387	-0.656210	0.881703
H	-1.588387	-0.656210	-0.881703
acetone			
0	1		
O	0.000000	0.000000	1.404559
C	0.000000	0.000000	0.184831
C	0.000000	1.286642	-0.616357
C	0.000000	-1.286642	-0.616357
H	0.000000	2.145130	0.055418
H	0.000000	-2.145130	0.055418
H	-0.881514	1.320931	-1.265003
H	0.881514	1.320931	-1.265003
H	0.881514	-1.320931	-1.265003
H	-0.881514	-1.320931	-1.265003
ethylene			
0	1		
C	0.000000	0.000000	0.669575
C	0.000000	0.000000	-0.669575
H	0.000000	0.926294	1.235309
H	0.000000	-0.926294	1.235309
H	0.000000	0.926294	-1.235309
H	0.000000	-0.926294	-1.235309
formaldehyde			
0	1		
C	0.000000	0.000000	-0.533319
O	0.000000	0.000000	0.679545
H	0.000000	0.937366	-1.118221
H	0.000000	-0.937366	-1.118221

isobutene

0	1		
C	0.000000	0.000000	1.463400
C	0.000000	0.000000	0.119614
H	0.000000	0.928447	2.027281
H	0.000000	-0.928447	2.027281
C	0.000000	1.276163	-0.680400
H	0.000000	2.156688	-0.032951
H	0.881626	1.321381	-1.330486
H	-0.881626	1.321381	-1.330486
C	0.000000	-1.276163	-0.680400
H	0.000000	-2.156688	-0.032951
H	-0.881626	-1.321381	-1.330486
H	0.881626	-1.321381	-1.330486

pyrazine

0	1		
C	0.000000	1.133849	0.699678
C	0.000000	1.133849	-0.699678
N	0.000000	0.000000	-1.420331
C	0.000000	-1.133849	-0.699678
C	0.000000	-1.133849	0.699678
N	0.000000	0.000000	1.420331
H	0.000000	2.068862	1.254885
H	0.000000	2.068862	-1.254885
H	0.000000	-2.068862	-1.254885
H	0.000000	-2.068862	1.254885

pyridazine

0	1		
N	0.000000	0.670782	-1.238620
C	0.000000	1.325351	-0.064846
C	0.000000	0.694555	1.184924
C	0.000000	-0.694555	1.184924
C	0.000000	-1.325351	-0.064846
N	0.000000	-0.670782	-1.238620
H	0.000000	1.274441	2.102771
H	0.000000	2.408352	-0.152902
H	0.000000	-1.274441	2.102771
H	0.000000	-2.408352	-0.152902

pyridine

0	1		
N	0.000000	0.000000	1.428332
C	0.000000	0.000000	-1.391651
C	0.000000	1.144923	0.723091
C	0.000000	-1.144923	0.723091
C	0.000000	-1.199476	-0.674912
C	0.000000	1.199476	-0.674912
H	0.000000	0.000000	-2.478143
H	0.000000	2.061946	1.308643
H	0.000000	-2.061946	1.308643
H	0.000000	-2.159346	-1.182856
H	0.000000	2.159346	-1.182856

pyrimidine

0	1		
C	0.000000	0.000000	-1.312458
N	0.000000	1.203128	-0.717845
C	0.000000	1.188178	0.625296
C	0.000000	0.000000	1.356430
C	0.000000	-1.188178	0.625296
N	0.000000	-1.203128	-0.717845
H	0.000000	2.156668	1.120301
H	0.000000	0.000000	-2.399286
H	0.000000	0.000000	2.441125
H	0.000000	-2.156668	1.120301

stetrazine

0	1		
C	0.000000	0.000000	1.265707
N	0.000000	1.203065	-0.667089
C	0.000000	0.000000	-1.265707
N	0.000000	-1.203065	0.667089
H	0.000000	0.000000	2.350926
H	0.000000	0.000000	-2.350926
N	0.000000	-1.203065	-0.667089
N	0.000000	1.203065	0.667089

tbutadiene

0	1		
C	0.608308	1.751027	0.000000
C	0.608308	0.403889	0.000000
C	-0.608308	-0.403889	0.000000

C	-0.608308	-1.751027	0.000000
H	1.533770	2.317122	0.000000
H	-0.322818	2.311810	0.000000
H	1.556392	-0.133780	0.000000
H	-1.556392	0.133780	0.000000
H	0.322818	-2.311810	0.000000
H	-1.533770	-2.317122	0.000000

REFERENCES

1 Kohn, W.; Sham, L. Self-consistent equations including exchange and correlation effects. J. *Phys. Rev.* **1965**, *140*, A1133-A1138.

2 (a) Gáspár, R. Uber eine approximation des Hartree-Fock schen potentials durch eine universelle potential function. Acta Phys. Hung. 1954, 3, 263-286. (b) Gáspár, R. Statistical exchange for electron in shell and the Xα method. *Acta Phys. Hung.* **1974**, *35*, 213-218.

3 Vosko, S. `H.; Wilk, L.; Nusair, M. Accurate spin-dependent electron liquid correlation energies for local spin density calculations: a critical analysis. *Can. J. Phys.* **1980**, *58*, 1200-1211.

4 Zhao, Y.; Truhlar, D. G. Construction of a generalized gradient approximation by restoring the density-gradient expansion and enforcing a tight Lieb–Oxford bound. *J. Chem. Phys.* **2008**, *128*, article no. 184109.

5 Perdew, J. P.; Ruzsinsky, A.; Csonka, G. I.; Vydrov, O. A.; Scuseria, G. E.; Constantin, L. A.; Zhou, X.; Burke, K. Restoring the density-gradient expansion for exchange in solids and surfaces. *Phys. Rev. Lett.* **2008**, *100*, article no. 136406.

6 Peverati, R.; Zhao, Y.; Truhlar, D. G. Generalized gradient approximation that recovers the second-order density-gradient expansion with optimized across-the-board performance. *J. Phys. Chem. Lett.* **2011**, *2*, 1991-1997.

7 Becke, A. D. Density functional calculations of molecular bond energies. *J. Chem. Phys.* **1986**, *84*, 4524-4529.

8 Perdew, J. P. Density-functional approximation for the correlation energy of the inhomogeneous electron gas. *Phys. Rev. B: Condens. Matter. Phys.* **1986**, *33*, 8822-8824.

9 Becke, A. D. Density-functional exchange-energy approximation with correct asymptotic behavior. *Phys. Rev. A* **1988**, *38*, 3098-3100.

10 Lee, C.; Yang, W.; Parr, R. G. Development of the Colle-Salvetti correlation-energy formula into a functional of the electron density. *Phys. Rev. B* **1988**, *37*, 785-789.

11 Becke, A. D.; Roussel, M. R. Exchange holes in inhomogeneous systems: A coordinate-space model. *Phys. Rev. A*, **1989**, *39*, 3761-3767.

12 Perdew, J. P. Unified theory of exchange and correlation beyond the local density approximation. In *Electronic Structure of Solids' 91*, Ziesche, P., Eschrig, H., Eds.; Akademie Verlag, Berlin, 1991, pp. 11-20.

13 Perdew, J. P.; Burke, K.; Ernzerhof, M. Generalized gradient approximation made simple. *Phys. Rev. Lett.* **1996**, *77*, 3865-3868.

14 Adamo, C.; Barone, V. Exchange functionals with improved long-range behavior and adiabatic connection methods without adjustable parameters: The *m*PW and *m*PW1PW models. *J. Chem. Phys.* **1998**, *108*, 664-675.

15 Zhang, Y.; Yang, W. Comment on "Generalized gradient approximation made simple" *Phys. Rev. Lett*. **1997**, *80*, 890.

16 Hammer, B.; Hansen, L.; Norskov, J. K. Improved adsorption energetics within density-functional theory using revised Perdew-Burke-Ernzerhof functionals. *Phys. Rev. B: Condens. Matter Mater. Phys.* **1999**, *59*, 7413-7421.

17 Boese, A. D.; Handy, N. C. A new parametrization of exchange–correlation generalized gradient approximation functionals. *J. Chem. Phys.* **2000**, *114*, 5497-5503.

18 Handy, N. C.; Cohen, A. Left-right Correlation energy. *J. Mol. Phys.* **2001**, *99*, 403-412.

19 Dahlke, E. E.; Truhlar, D. G.; Improved density functionals for water. *J. Phys. Chem. B* **2005**, *109*, 15677-15683.

20 Schultz, N.E.; Zhao, Y. Truhlar, D. G.; Density functionals for inorganometallic and organometallic chemistry. *J. Phys. Chem. A* **2005**, *109*, 11127-11143.

21 Grimme, S. Semiempirical GGA-type density functional constructed with a long-range dispersion correction. *J. Comput. Chem*. **2006**, *27*, 1787-1799.

22 Zhao, Y.; González-García, N.; Truhlar, D. G. Benchmark database of barrier heights for heavy atom transfer, nucleophilic substitution, association, and unimolecular reactions and its use to test theoretical methods. *J. Phys. Chem. A*, **2005**, *109*, 2012-2018.

23 Thakkar, A. J.; McCarthy, S. P. Toward improved density functionals for the correlation energy. *J. Chem. Phys.* **2009**, *131*, article no. 134109.

24 Peverati, R.; Truhlar, D. G. Exchange–correlation functional with good accuracy for both structural and energetic properties while depending only on the density and its gradient. *J. Chem. Theory Comput.* **2012**, *8*, 2310-2319.

25 Yu, H. S; Zhang, W.; Verma, P.; He, X.; Truhlar, D. G. Nonseparable exchange–correlation functional for molecules, including homogeneous catalysis involving transition metals. *Phys. Chem. Chem. Phys.* **2015**, *17*, 12146-12160.

26 Voorhis, T. V.; Scuseria, G. E. A novel form for the exchange-correlation energy functional. *J. Chem. Phys*. **1998**, *109*, 400-410.

27 Boese, A. D.; Handy, N. C. New exchange-correlation density functionals: The role of the kinetic-energy density. *J. Chem. Phys.* **2002**, *116*, 9559-9569.

28 Tao, J. M.; Perdew, J. P.; Staroverov, V. N.; Scuseria, G. E. Climbing the density functional ladder: nonempirical meta–generalized gradient approximation designed for molecules and solids. *Phys. Rev. Lett.,* **2003**, *91*, article no. 146401.

29 Zhao, Y.; Truhlar, D. G. A new local density functional for main-group thermochemistry, transition metal bonding, thermochemical kinetics, and noncovalent interactions. *J. Chem. Phys.,* **2006**, *125*, article no. 194101.

30 Perdew, J. P.; Ruzsinszky, A.; Csonka, G. I.; Constantin, L. A.; Sun J. Workhorse semilocal density functional for condensed matter physics and quantum chemistry. *Phys. Rev. Lett.,* **2009**, *103*, article no. 026403.

31 Peverati, R. Truhlar, D. G. M11-L: A local density functional that provides improved accuracy for electronic structure calculations in chemistry and physics. *J. Phys. Chem. Lett.* **2011**, *3*, 117-124.

32 Sun, J.; Haunschild, R.; Xiao, B.; Bulik, I. W.; Scuseria, G. E.; Perdew, J. P. Semilocal and hybrid meta-generalized gradient approximations based on the understanding of the kinetic-energy-density dependence. *J. Chem. Phys.* **2013**, *138*, article no. 044113.

33 Peverati, R.; Truhlar, D. G. An improved and broadly accurate local approximation to the exchange–correlation density functional: The MN12-L functional for electronic structure calculations in chemistry and physics. *Phys. Chem. Chem. Phys.* **2012**, *14*, 13171-13174.

34 Yu. H. S.; He, X.; Truhlar, D. G. MN15-L: A New Local Exchange-Correlation Functional for Kohn-Sham Density Functional Theory with Broad Accuracy for Atoms, Molecules, and Solids. *J. Chem. Theory Comput*. 2016, available online as Article ASAP. DOI: 10.1021/acs.jctc.5b01082

35 Roothaan, C. C. J. New developments in molecular orbital theory. *Rev. Mod. Phys.* **1951**, *23*, 69-89.

36 Becke, A. D. Density-functional thermochemistry. 3. The role of exact exchange. *J. Chem. Phys.* **1993**, *98*, 5648-5652.

37 Stephens, P. J.; Devlin, F. J.; Chabalowski, C. F.; Frisch, M. J. *Ab initio* calculation of vibrational absorption and circular dichroism spectra using density functional force fields. *J. Phys. Chem*. **1994**, *98*, 11623-11627.

38 Adamo, C.; Barone, V. Toward reliable density functional methods without adjustable parameters: The PBE0 model. *J. Chem. Phys.* **1999**, *110*, 6158-6169.

39 Adamo, C.; Barone, V. Exchange functionals with improved long-range behavior and adiabatic connection methods without adjustable parameters: the mPW and mPW1PW models. *J. Chem. Phys.* **2004**, *108*, 6908-6918.

40 Adamo, C.; Barone, V. Toward reliable adiabatic connection models free from adjustable parameters. *Chem. Phys. Lett.* **1997**, *274*, 242-250.

41 Schmider, H. L.; Becke, A. D. Optimized density functionals from the extended G2 test set. *J. Chem. Phys*. **1998**, *108*, 9624-9631.

42 Hamprecht, F. A.; Cohen, A. J.; Tozer, D. J.; Handy, N. C. Development and assessment of new exchange-correlation functionals. *J. Chem. Phys.* **1988**, *109*, 6264-6271.

43 Lynch, B. J.; Fast, P. L.; Harris, M.; Truhlar, D. G. Adiabatic connection for kinetics. J. Phys. Chem. A **2000**, *104*, 4811-4815.

44 Hoe, W.-M.; Cohen, A. J.; Handy, N. C. Assessment of a new local exchange functional OPTX. *Chem. Phys. Lett.* **2001**, *341*, 319-328.

45 Wilson, P. J.; Bradley, T. J.; Tozer, D. J. Hybrid exchange-correlation functional determined from thermochemical data and ab initio potentials. *J. Chem. Phys.* **2001**, *115*, 9233-9242.

46 Zhao, Y.; Truhlar, D. G. Hybrid meta density functional theory methods for thermochemistry, thermochemical kinetics, and noncovalent interactions: the MPW1B95 and MPWB1K models and comparative assessments for hydrogen bonding and vander Waals interactions. *J. Phys. Chem. A* **2004**, *108*, 6908-6918.

47 Keal, T. W.; Tozer, D. J. Semiempirical hybrid functional with improved performance in an extensive chemical assessment. *J. Chem. Phys*. **2005**, *123*, article no. 121103.

48 Peverati, R.; Truhlar, D. G. Communication: A global hybrid generalized gradient approximation to the exchange-correlation functional that satisfies the second-order density-gradient constraint and has broad applicability in chemistry. *J. Chem. Phys*. **2011**, 135, article no. 191102.

49 Yanai, T.; Tew, D.; Handy, N. A new hybrid exchange–correlation functional using the Coulomb-attenuating method (CAM-B3LYP). *Chem. Phys. Lett.* **2004**, *393*, 51-57.

50 Tawada, Y.; Tsuneda, T.; Yanagisawa, S.; Yanai, T.; Hirao, K. A long-range-corrected time-dependent density functional theory. *J. Chem. Phys*. **2004**, *120*, 8425-8433.

51 Vydrov, O. A.; Scuseria, G. E. Assessment of a long-range corrected hybrid functional. *J. Chem. Phys.* **2006**, *125*, article no. 234109.

52 Vydrov, O. A.; Heyd, J.; Krukau, A. V.; Scuseria, G. E. Importance of short-range versus long-range Hartree-Fock exchange for the performance of hybrid density functionals. *J. Chem. Phys.* **2006**, *125*, article no. 074106.

53 Vydrov, O. A.; Scuseria, G. E.; Perdew, J. P. Tests of functionals for systems with fractional electron number. *J. Chem. Phys.* **2007**, *126*, 154109.

54 Heyd, J.; Scuseria, G. E.; Ernzerhof, M. Hybrid functionals based on a screened Coulomb potential. *J. Chem. Phys.* **2003**, *118*, 8207-8215.

55 Henderson, T. M.; Izmaylov, A. F.; Scalmani, G.; Scuseria, G. E. Can short-range hybrids describe long-range-dependent properties? *J. Chem. Phys.* **2009**, *131*, article no. 044108.

56 Chai, J.-D.; Head-Gordon, M. Systematic optimization of long-range corrected hybrid density functionals. *J. Chem. Phys.* **2008**, *128*, 084106.

57 Chai, J.-D.; Head-Gordon, M. Long-range corrected hybrid density functionals with damped atom–atom dispersion corrections. *Phys. Chem. Chem. Phys.* **2008**, *10*, 6615-6620.

58 Peverati, R.; Truhlar, D. G. Screened-exchange density functionals with broad accuracy for chemistry and solid-state physics. *Phys. Chem. Chem. Phys.* **2012**, *14*, 16187-16191.

59 Staroverov, V. N.; Scuseria, G. E.; Tao, J.; Perdew, J. P. Comparative assessment of a new nonempirical density functional: Molecules and hydrogen-bonded complexes. *J. Chem. Phys*. **2003**, *119*, 12129-12137.

60 Becke, A. D. Density-functional thermochemistry. IV. A new dynamical correlation functional and implications for exact-exchange mixing. *J. Chem. Phys.* **1996**, *104*, 1040-1046.

61 Zhao, Y.; Lynch, B. J.; Truhlar, D. G. Development and assessment of a new hybrid density functional model for thermochemical kinetics. *J. Phys. Chem*. A **2004**, *108*, 2715-2719.

62 Boese, A. D.; Martin, M. L. Development of density functionals for thermochemical kinetics. *J. Chem. Phys.* **2004**, *121*, 3405-3416.

63 Zhao, Y.; Lynch, B. J. Truhlar, D. G. Multi-coefficient extrapolated density functional theory for thermochemistry and thermochemical kinetics. *Phys. Chem. Chem. Phys.* **2005**, *7*, 43-52.

64 Zhao, Y.; Truhlar, D. G. Benchmark databases for nonbonded interactions and their use to test density functional theory. *J. Chem. Theory Comput.* **2005**, *1*, 145-432.

65 Zhao, Y.; Truhlar, D. G. Design of density functionals that are broadly accurate for thermochemistry, thermochemical kinetics, and nonbonded interactions. *J. Phys. Chem. A* **2005**, *109*, 5656-5667.

66 Zhao, Y.; Schultz, N. E.; Truhlar, D. G. Exchange-correlation functional with broad accuracy for metallic and nonmetallic compounds, kinetics, and noncovalent interactions. *J. Chem. Phys.* **2005**, *123*, 161103.

67 Zhao, Y.; Schultz, N. E.; Truhlar, D. G. Design of density functionals by combining the method of constraint satisfaction with parametrization for thermochemistry, thermochemical kinetics, and noncovalent interactions. *J. Chem. Theory Comput.* **2005**, *2*, 364-382.

68 Zhao, Y.; Truhlar, D. G. Density functional for spectroscopy: no long-range self-interaction error, good performance for Rydberg and charge-transfer states, and better performance on average than B3LYP for ground states. *J. Phys. Chem. A.* **2006**, *110*, 13126-13130.

69 Zhao, Y.; Truhlar, D. G. The M06 suite of density functionals for main group thermochemistry, thermochemical kinetics, noncovalent interactions, excited states, and transition elements: two new functionals and systematic testing of four M06-class functionals and 12 other functionals. *Theor. Chem. Acc.* **2008**, *120*, 215-241.

70 Zhao, Y.; Truhlar, D. G. Exploring the limit of accuracy of the global hybrid meta density functional for main-group thermochemistry, kinetics, and noncovalent interactions. *J. Chem. Theory Comput.* **2008**, *4*, 1849-1868.

71 Peverati, R.; Truhlar, D. G. Improving the accuracy of hybrid meta-gga density functionals by range separation. *J. Phys. Chem. Lett.* **2011**, *2*, 2810-2817.

72 F. Weigend, R. Ahlrichs, *Phys. Chem. Chem. Phys*. 2005, **7**, 3297-3305.

73 J. Zheng, X. Xu, D. G. Truhlar, *Theor. Chem. Acc.* 2010, **128**, 295-305

74 K. B. Wiberg, A. E. de Oliveira, G. Trucks, Effect of Basis sets. *J. Phys. Chem. A* 2002, **106**, 4192-4199.

75 T. H. Dunning, Jr. *J. Chem. Phys.* 1989, **90**, 1007-1023.

76 T. H. Dunning, Jr. K. A. Peterson, A. K. Wilson, *J. Chem. Phys.* 2001, **114**, 9244-9253.

77 K. A. Peterson, D. Figgen, M. Dolg, H. Stoll, *J. Chem. Phys.* 2007, **126**, 124101.

78 D. Figgen, K. A. Peterson, M. Dolg, H. Stoll, *J. Chem. Phys.* 2009, **130**, 164108.

79 D. E. Woon, T. H. Dunning, Jr. *J. Chem. Phys*. 1993, **98**, 1358-1371.

80 L. Hu, H. Chen, *J. Chem. Theory Comput.* 2015, **11**, 4601-4614.

81 Y. Sun, H. Chen, *J. Chem. Theory Comput.* 2014, **10**, 579-588.

82 Y. Sun, H. Chen, *J. Chem. Theory Comput.* 2013, **9**, 4735-4743.

83 M. Piccardo, E. Penocchio, C. Puzzarini, M. Biczysko, V. Barone, *J. Phys. Chem. A* 2015, **119**, 2058-2082.
